# Recent Advances in Ferrite‐Based Materials for Biomedical Applications: A Comprehensive Review

**DOI:** 10.1002/adma.74149

**Published:** 2026-07-21

**Authors:** Pramod D. Mhase, Varsha C. Pujari, Akash V. Fulari, Sunil M. Patange, Sean Li, Danyang Wang, Sagar E. Shirsath

**Affiliations:** ^1^ Materials Science Research Laboratory Shri Krishna Mahavidyalaya Dharashiv Maharashtra India; ^2^ Symbosis Centre for Nanoscience and Nanotechnology Symbiosis International (Deemed University) Pune India; ^3^ School of Materials Science and Engineering University of New South Wales Sydney New South Wales Australia; ^4^ UNSW Materials and Manufacturing Futures Institute University of New South Wales Sydney New South Wales Australia

**Keywords:** ferrite nanoparticles, magnetic hyperthermia, magnetotheranostics, nanozymes, surface engineering, targeted drug delivery

## Abstract

Ferrite‐based nanomaterials have evolved from simple magnetic contrast agents into sophisticated, multifunctional platforms capable of actively regulating biological microenvironments. This comprehensive review critically examines the recent advances in the rational design of spinel and hexagonal ferrites, elucidating the fundamental structure–property–bioactivity relationships that govern their biomedical performance. We explore how atomic‐level engineering, specifically cation distribution, defect modulation, and morphological anisotropy, can be manipulated to tailor magnetic susceptibility, catalytic activity (nanozymes), and specific absorption rates (SAR) for hyperthermia. Beyond traditional applications in MRI and drug delivery, we highlight emerging frontiers including viscosity‐independent magnetic hyperthermia, ROS‐mediated antimicrobial therapy, and magnetic tissue engineering (Mag‐TE). Crucially, this review addresses the widening translational gap between high‐performance laboratory prototypes and clinical reality. We critically analyze the barriers impeding commercialization, such as the discrepancy between colloidal and intracellular heating efficiency, the complexity of protein corona formation, and the challenges of scalable, GMP‐compliant synthesis. Finally, we propose a future roadmap integrating AI‐driven material discovery and green chemistry to develop next‐generation magnetotheranostic systems, positioning ferrite nanoplatforms as central components in the future of precision medicine.

## Graphical abstract

Ferrite nanoplatforms are presented as tunable biomedical materials in which synthesis control, cation engineering, defect/morphology regulation, and surface functionalization govern structure–property–bioactivity relationships. These design strategies enable multifunctional applications including MRI contrast, magnetic hyperthermia, targeted drug delivery, nanozyme/antimicrobial action, tissue engineering, and clinical translation toward safer precision nanomedicine.

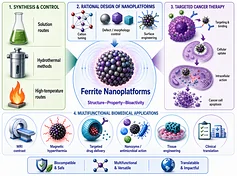

## 1 Introduction

Ferrites are a class of ceramic compounds with the general chemical formula MFe_2_O_4_, where M is typically a divalent metal cation such as iron (Fe^2+^), cobalt (Co^2+^), manganese (Mn^2+^), nickel (Ni^2+^), or zinc (Zn^2+^). These materials inherently possess a spinel crystal structure, which dictates their ferrimagnetic or superparamagnetic behavior when synthesized at the nanoscale [1]. This magnetic tunability, combined with their low toxicity and chemical stability, forms the fundamental basis for their extensive utility in the biomedical field. The various ferrite materials such as, zinc ferrite (ZnFe_2_O_4_) and copper ferrite (CuFe_2_O_4_) display intrinsic antimicrobial activity [2, 3], while manganese ferrite (MnFe_2_O_4_) nanoparticles have shown promise as MRI contrast agents and magnetic hyperthermia mediators [4]. Ferrite materials, particularly magnetic ferrite nanoparticles, have emerged as remarkably versatile and promising nanostructures within the field of biomedicine [5]. Nanoparticles, due to their exceptionally small size combined with distinctive physical and chemical properties, have unlocked new possibilities in the diagnosis, treatment, and continuous monitoring of various diseases. Their nanoscale dimensions enable them to interact intimately with biological systems at the molecular level, allowing for targeted and highly specific interventions. In biomedical applications, this translates to enhanced clinical outcomes by improving the precision of diagnostics and therapies, while simultaneously reducing unwanted side effects commonly associated with conventional treatments. These advancements pave the way for more personalized and efficient healthcare approaches aimed at mitigating risks and maximizing therapeutic benefits [6, 7]. Magnetic ferrites intrinsic magnetic properties allow them to respond predictably to external magnetic fields, an ability that has been ingeniously harnessed in biomedical technologies ranging from improved imaging to advanced treatment modalities. These nanoparticles possess an exceptional surface‐to‐volume ratio, which enhances their interaction with biological systems and makes them adaptable to functionalization with targeting molecules, drugs, or imaging agents [8, 9]. This tunability opens up powerful avenues for their use, such as magnetic resonance imaging (MRI), where ferrite nanoparticles serve as effective contrast agents to enhance diagnostic accuracy [10]. Their capacity to generate localized heat under an alternating magnetic field has been exploited in magnetic hyperthermia for cancer therapy offering a minimally invasive option that selectively destroys malignant cells while sparing healthy tissue [11, 12].

Beyond diagnostics and thermal therapy, ferrite nanoparticles have gained traction as vehicles for targeted drug delivery. Their magnetic responsiveness allows for precise navigation and concentration of therapeutic agents to specific tissue sites, crucial for minimizing systemic side effects and maximizing therapeutic efficacy [13]. Moreover, ferrites exhibit inherent antimicrobial properties that are proving valuable in tackling resistant bacterial infections, extending their potential to infection control alongside other biomedical uses [14]. Recent advancements have been fueled by remarkable progress in their synthesis, enabling precise control over size, shape, and surface chemistry factors critical to optimizing their functionality and safety in biomedical contexts [15]. Various approaches such as sol–gel synthesis, hydrothermal methods, and green chemistry protocols have improved particle uniformity and biocompatibility, facilitating enhanced bio‐distribution and cellular uptake.

**FIGURE 1:**
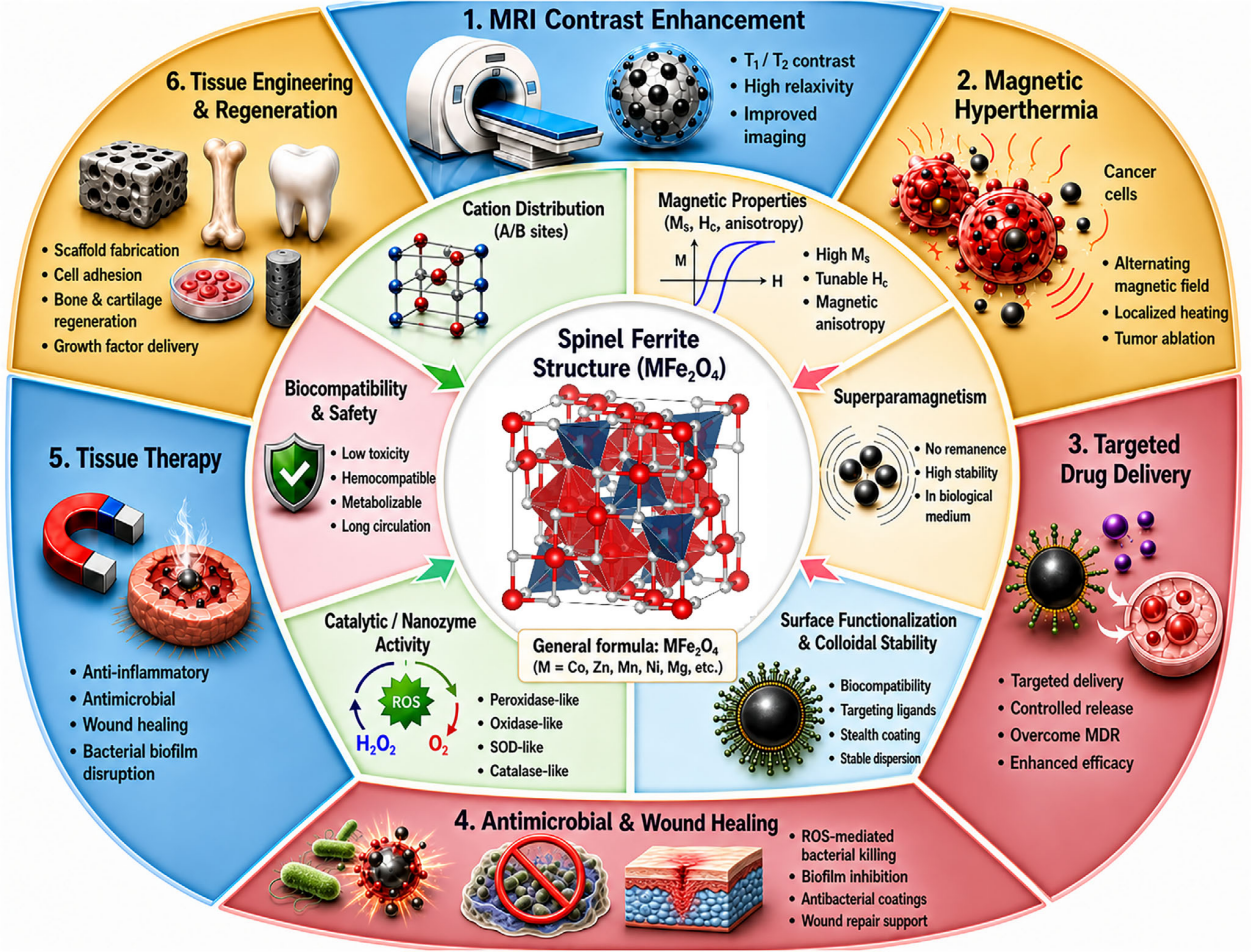
Structure–property–function relationship of ferrite nanoparticles, illustrating how spinel ferrite structure (MFe_2_O_4_) governs physicochemical properties and enables diverse biomedical applications.

The significance of ferrite materials in biomedicine is further reinforced by their multifunctionality. Beyond their magnetic attributes, ferrites exhibit semiconducting, catalytic, and antibacterial properties, which broaden their applicability to biosensing, bioelectronics, and regenerative medicine. The ability to combine magnetic, electronic, and biological functions in a single material platform represents a paradigm shift toward integrated, multifunctional biomedical materials. To provide a comprehensive understanding of ferrite‐based nanomaterials, it is essential to correlate their intrinsic crystal structure with resultant physicochemical properties and biomedical functionality. As illustrated in Figure 1, the spinel ferrite structure (MFe_2_O_4_), governed by cation distribution between tetrahedral (A) and octahedral (B) sites, plays a decisive role in defining magnetic behavior, surface reactivity, and biocompatibility. These fundamental properties, including saturation magnetization, anisotropy, and surface functionalization capability, directly influence their performance in biomedical applications such as magnetic hyperthermia, MRI contrast enhancement, and targeted drug delivery.

To emphasize the practical and industrial importance of magnetic nanoparticles, it is valuable to include current market projections that contextualize their global economic impact. Recent market research indicates that the global magnetic nanoparticles market is experiencing significant growth driven by demand in biomedical applications (e.g., imaging, drug delivery, and hyperthermia), environmental remediation, and electronics. For example, one industry forecast reports that the market for magnetite nanoparticles, one of the most widely studied magnetic nanomaterials, was valued at approximately USD 89.8 million in 2024 and is expected to grow to USD 204.8 million by 2033, exhibiting a compound annual growth rate (CAGR) of about 9.1 % over the forecast period (https://www.imarcgroup.com/magnetite‐nanoparticles‐market). In a broader assessment of the magnetic nanoparticles sector, several market analyses project continued expansion, with estimates suggesting that the magnetic nanoparticles market could exceed USD 189.8 million by 2032, driven by rising adoption in healthcare, diagnostics, and advanced material applications (https://www.acumenresearchandconsulting.com/magnetic‐nanoparticles‐market).

From a regulatory perspective, iron oxide‐based ferrite nanoparticles represent the most clinically advanced magnetic nanomaterials. Notably, Fe_3_O_4_ (magnetite) nanoparticles have previously received U.S. FDA approval for use as MRI contrast agents, establishing a critical safety and translational benchmark [16]. These approvals confirmed acceptable biocompatibility, metabolic iron clearance, and manageable toxicity profiles under clinically relevant doses. Importantly, the subsequent market withdrawal of several iron oxide formulations was largely attributed to commercial and logistical factors rather than regulatory safety concerns. This regulatory precedent underscores the translational potential of ferrite‐based powders, particularly spinel iron oxides, for advanced biomedical applications [17]. However, extending FDA‐approved success to doped ferrites and hybrid systems requires stringent control over powder synthesis, phase purity, surface functionalization, and batch‐to‐batch reproducibility. Addressing these powder‐processing challenges is essential to ensure consistent biological performance and regulatory compliance, thereby bridging the gap between laboratory‐scale innovation and clinical deployment.

**FIGURE 2:**
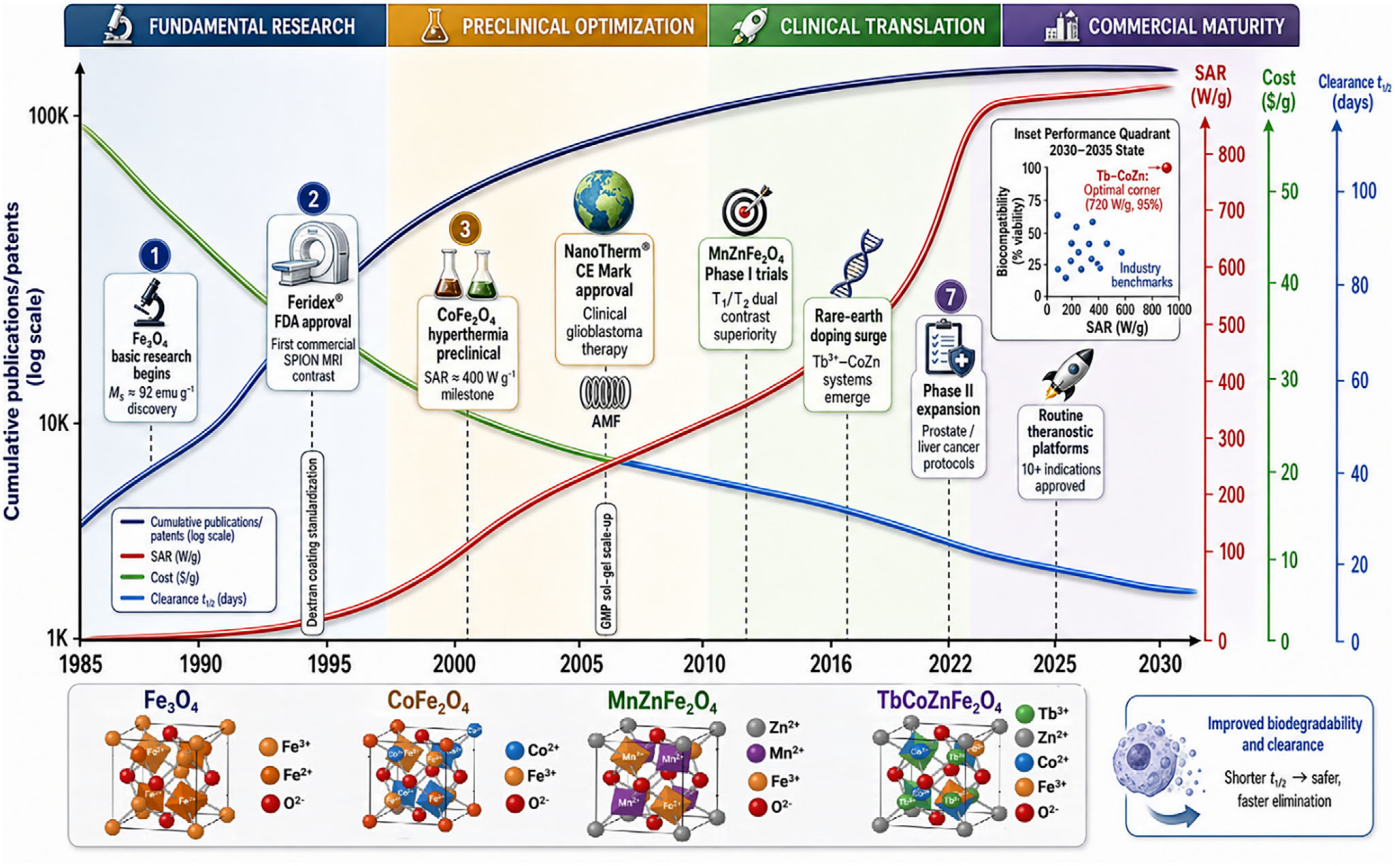
Chronological roadmap of spinel ferrite nanoparticle evolution (1985–2030). The bottom‐panel spinel structures are schematic illustrations for visual representation only and do not correspond to exact crystallographic structures.

Figure 2 demonstrates a chronological roadmap of spinel ferrite nanoparticle evolution (1985–2030). The timeline delineates the transition from fundamental characterization to clinical translation, highlighted by key regulatory milestones (Feridex approval, NanoTherm CE Mark). The overlay curves demonstrate the convergence of exponentially growing academic output (blue fill) with optimizing performance metrics: increasing specific absorption rate (SAR, red), reducing production costs (green), and faster physiological clearance rates (blue). The inset (top right) positions novel Tb^3+^‐doped CoZn ferrites as a leading candidate in the current Clinical Translation era, exhibiting a superior balance of heating efficiency and biocompatibility relative to legacy formulations. To contextualize the current advances in rare‐earth doping, Figure 2 summarizes the three‐decade trajectory of spinel ferrite technology. The field has progressed through distinct phases: initial fundamental discovery (1985–2000), preclinical optimization of heating efficiency (2000–2015), and the current phase of clinical translation and defect engineering (2015–present). While early‐generation particles (Feridex) established regulatory precedents, modern formulations like the Tb^3+^‐doped system highlighted here address critical barriers in hyperthermia therapy by maximizing SAR (>700 W/g) while ensuring rapid physiological clearance, positioning them for Phase II trials by 2025. The trends illustrated in Figure 2 highlight that the performance of ferrite nanomaterials is strongly governed by the interplay between composition, particle size, and cation distribution. In particular, increased magnetic anisotropy enhances hyperthermia efficiency but may compromise biocompatibility and colloidal stability. This suggests that optimal material design requires balancing magnetic performance with biological compatibility, rather than maximizing a single parameter.

To address various challenges, a rational design roadmap for ferrite nanomaterials can be proposed. First, compositional engineering (like Co, Mn, Zn substitution) should be employed to tailor magnetic anisotropy and enhance functional performance such as SAR and relaxivity. Second, particle size and morphology control in the range of 5–20 nm is critical to achieve superparamagnetic behavior while maintaining high magnetic response. Third, surface functionalization strategies, including polymer coatings and bio‐ligand conjugation, are essential to improve colloidal stability, reduce cytotoxicity, and enable targeted delivery. Fourth, the development of core–shell and hybrid nanostructures can provide synergistic enhancement in multifunctional applications. Finally, standardization of experimental conditions and reporting metrics is necessary to enable reliable comparison across studies and accelerate clinical translation.

Overall, ferrite‐based materials represent a versatile and rapidly evolving class of magnetic oxides with enormous potential for biomedical applications. Their unique combination of tunable magnetism, structural robustness, and biocompatibility, coupled with powder‐based scalability and eco‐friendly synthesis, has positioned them at the forefront of next‐generation biomedical technologies [18, –, 20]. The convergence of advanced materials design, nanotechnology, and bioengineering has accelerated innovations across imaging, therapy, sensing, and regenerative medicine. The following sections of this review will systematically explore the fundamental aspects, recent trends, synthesis strategies, biomedical functionalities, and translational challenges of ferrite‐based materials, providing an integrated perspective on their current status and future prospects in biomedical science.

## 2 Overview of Recent Trends

Over the past decade, ferrite materials have garnered substantial attention in biomedical research due to their intriguing structural, magnetic, and chemical properties at the nanoscale. Advances in controlled synthesis, surface functionalization, and composite formation have driven the diversification of their biomedical applications, evolving ferrites from fundamental nanomaterials into sophisticated multifunctional platforms. Ferrite nanoparticles exhibit magnetic anisotropy and tunable Curie temperatures that allow them to generate heat when subjected to an alternating magnetic field. This phenomenon, termed magnetic hyperthermia, enables localized thermal ablation of cancerous tissues without affecting surrounding healthy cells [21]. Recent studies have also demonstrated that magnetic nanoparticles (MNPs) can be effectively cleared from the body through blood circulation or phagocytic uptake in organs such as the liver, spleen, and lymph nodes after treatment, thereby minimizing residual accumulation and reducing damage to healthy tissues [22]. Magnetic hyperthermia therapy (MHT) using these nanoparticles generates localized heating at the subcellular level, specifically targeting organelles like lysosomes to induce cell death through lysosomal pathways [23]. Additionally, MHT promotes the generation of reactive oxygen species (ROS) at these targeted sites [24], which further enhances therapeutic efficacy by invoking specific biological responses [25]. Significant advancements have also been made in the targeted delivery and controlled release of therapeutic agents using MNP‐based nanomaterials. Importantly, ongoing clinical trials and translational studies underscore the potential of MNP‐mediated MHT as a promising modality for cancer treatment [26, 27]. The specific absorption rate (SAR), which quantifies heating efficiency, is critically dependent on intrinsic parameters like particle size, crystallinity, and cation distribution within the spinel lattice [28, 29]. Hybrid composites combining ferrites with carbonaceous or noble metal components have demonstrated enhanced heat generation through synergistic photothermal effects, offering dual therapeutic modalities that improve treatment precision and efficacy [30].

Importantly, recent progress in magnetic hyperthermia should not be evaluated solely from the viewpoint of ferrite nanoparticle composition, particle size, anisotropy, or SAR. The therapeutic efficiency of magnetic hyperthermia is also constrained by the design of the magnetic‐field generation system, including coil geometry, field homogeneity, frequency–amplitude window, power‐transfer efficiency, heat dissipation in the applicator, and induced electric‐field distribution in the biological target region. Lei et al. [31] recently demonstrated a parallel resonant magnetic‐field generator for biomedical applications, showing that system‐level parameters such as circuit quality factor, coil resistance, switching behavior, and spatial–temporal magnetic‐field distribution strongly influence the delivered field environment. This work provides an important systems‐engineering perspective for interpreting nanoparticle heating performance because even highly optimized ferrite nanoparticles may show limited therapeutic efficiency if the external field delivery system cannot generate sufficiently uniform, safe, and reproducible magnetic exposure. Therefore, magnetic hyperthermia should be considered as an integrated nanoparticle–field‐generator–biological microenvironment problem rather than as a property determined exclusively by intrinsic nanoparticle SAR.

Ferrite nanoparticles have emerged as promising alternatives to conventional gadolinium‐based agents used in MRI, addressing concerns such as nephrotoxicity and unstable chelation. Their superparamagnetic behavior enhances proton relaxivity, thereby increasing image contrast [32, 33]. Fine‐tuning magnetic moment and hydrodynamic particle size, coupled with biocompatible surface coatings like polyethylene glycol (PEG), dextran, or silica, prolong circulation time and reduce immunogenicity. By modifying these parameters, the nanoparticles can be engineered to act as T1 or T2 contrast agents, optimizing diagnostic capabilities [34, 35, 36]. The intrinsic magnetic responsiveness of ferrite nanoparticles facilitates magnetic targeting wherein an external magnetic field directs drug‐loaded carriers to specific biological sites, enhancing therapeutic concentration and reducing systemic toxicity [37, 38]. Drugs can be loaded onto ferrite nanoparticles via adsorption, encapsulation, or covalent bonding, enabling controlled release [39, 40]. Integration of diagnostic imaging with therapeutic functions, termed theranostics, allows simultaneous disease detection, treatment monitoring, and drug delivery, creating personalized and adaptive therapeutic platforms [41, 42, 43].

Ferrites possess magnetic and electronic properties conducive to biosensing. Their integration into magneto‐immunoassays, magnetic relaxation sensors, and electrochemical biosensors amplifies sensitivity and specificity for biomolecule, pathogen, and toxin detection [44, 45]. The catalytic activity inherent to some ferrites underpins the development of enzyme‐mimicking nanomaterials (nanozymes), which offer robust, rapid, and cost‐effective detection methods amendable to point‐of‐care diagnostics [46]. Certain ferrite compositions exhibit intrinsic antibacterial activity by generating reactive oxygen species (ROS) and disrupting bacterial membranes, making them suitable for antimicrobial coatings on implants and wound dressings [47, 48]. Incorporation of ferrite nanoparticles into polymeric or ceramic scaffolds leverages magnetic stimulation and mechanical reinforcement to promote osteogenic differentiation and tissue regeneration [49, 50]. Magnetically responsive scaffolds enable controlled mechanical cues to cells, advancing regenerative medicine strategies [51]. Environmental concerns have spurred efforts toward sustainable synthesis of ferrite nanoparticles employing biological extracts, amino acids, or microbial cultures [52, 53]. These biogenic methods afford precise control over nanoparticle size and morphology while enhancing surface biocompatibility, aligning nanomaterial fabrication with eco‐friendly principles and scalable production [54].

Recent trends emphasize the fabrication of hybrid composites, wherein ferrites are combined with graphene, perovskites, or metal oxides to realize synergistic effects. These multifunctional nanostructures integrate magnetic, optical, and electronic traits, enabling multimodal imaging, photothermal therapy, and advanced biosensing [55, 56]. Emerging additive manufacturing technologies further facilitate the incorporation of ferrite powders into complex biomedical devices and implants, expanding their functional applications [57, 58, 59]. Collectively, these theoretical insights underscore the transformation of ferrite materials into versatile platforms with broad biomedical utility. Realizing their full potential necessitates addressing challenges including long‐term biocompatibility, biodistribution, and reproducibility of synthesis processes, which remain central to clinical translation efforts.

A persistent controversy in ferrite‐mediated hyperthermia is the mismatch between high SAR values measured in well‐dispersed colloidal suspensions and the often‐reduced or mechanistically altered heating response observed after cellular internalization. In colloids, both Brownian and Néel relaxation may contribute to heat generation, whereas intracellular confinement in endosomes/lysosomes suppresses Brownian rotation and shifts the heating mechanism toward Néel relaxation, hysteresis losses, magneto‐mechanical effects, or ROS‐mediated biological stress. Consequently, SAR measured in water or buffer should be treated as a screening metric rather than a direct predictor of intracellular therapeutic efficacy.

## 3 Classification of Ferrites in Biomedical Applications

Ferrites constitute a broad class of mixed metal oxides containing iron as a principal component, typically exhibiting ferrimagnetic or superparamagnetic behavior. Their diverse structural types and tunable magnetic characteristics make them ideal candidates for biomedical applications ranging from targeted drug delivery to magnetic resonance imaging (MRI), hyperthermia, and tissue engineering.

**FIGURE 3:**
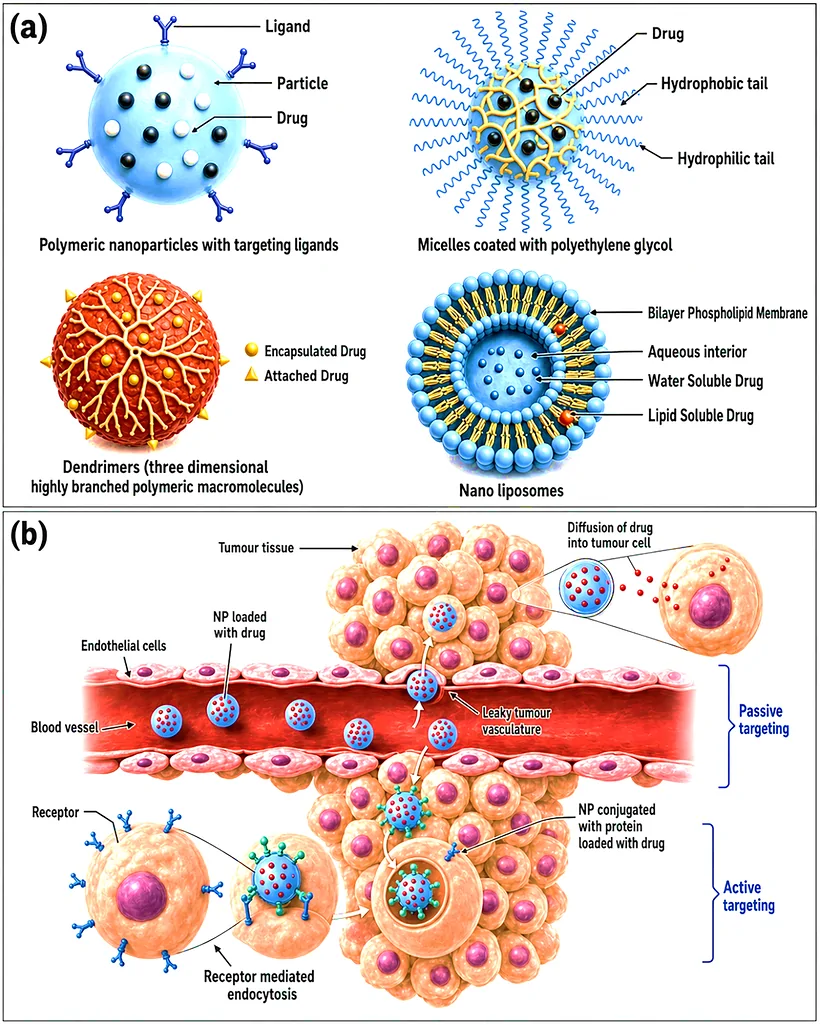
(a) Representative nanocarrier systems for drug delivery, including polymeric nanoparticles, polyethylene glycol‐coated micelles, dendrimers, and nanoliposomes. (b) Schematic illustration of passive targeting via enhanced vascular permeability and active targeting via receptor‐mediated endocytosis for tumor‐directed drug delivery. (a) Reproduced under the terms of the CC‐BY Creative Commons Attribution 4.0 International license (http://creativecommons.org/licenses/by/4.0/) [60]. Copyright 2020, The Authors, published by Frontiers. (b) Reproduced under the terms of the CC‐BY Creative Commons Attribution 4.0 International license (http://creativecommons.org/licenses/by/4.0/) [61]. Copyright 2023, The Authors, published by Frontiers.

To achieve site‐specific delivery and minimize systemic toxicity, ferrite nanoparticles are frequently surface‐engineered with targeting ligands. Figure 3a schematically illustrates the diverse classes of ligand‐based nanocarriers, categorizing them into macromolecular targeting agents (such as monoclonal antibodies and proteins) and small‐molecule ligands (including peptides, folic acid, and carbohydrates). These surface moieties are critical for ‘active targeting,’ as they specifically bind to receptors overexpressed on tumor cell surfaces, thereby facilitating cellular uptake via receptor‐mediated endocytosis [60]. Passive targeting exploits the unique pathophysiological characteristics of the tumor microenvironment, specifically the enhanced permeability and retention (EPR) effect. Characterized by hyperpermeable vasculature and defective lymphatic drainage, the EPR effect facilitates the preferential extravasation and prolonged retention of nanocarriers within the tumor interstitium. This mechanism significantly enhances the intratumoral bioavailability of therapeutic agents while minimizing systemic exposure and toxicity associated with free drug administration, as illustrated in Figure 3b [61].

The classification of ferrites can be approached from multiple perspectives, such as crystallographic structure, magnetic ordering, chemical composition, and functional applicability. Each category provides distinct insights into the design rationale and suitability of specific ferrite systems for biomedical deployment.

### 3.1 Structural Classification of Ferrites

The ferrites are mainly classified into three families: spinel ferrites, hexagonal ferrites, and garnet ferrites. Each structure determines cation distribution, magnetic behavior, and physicochemical properties, which in turn dictate their biomedical functionalities.

#### 3.1.1 Spinel Ferrites (MFe_2_O_4_)

Spinel ferrites, having the general formula MFe_2_O_4_, represent the most extensively studied class of ferrites in biomedicine. They crystallize in the cubic close‐packed oxygen lattice with cations occupying both tetrahedral (A) and octahedral (B) interstitial sites. Depending on the cation distribution, they can exist as normal spinels like ZnFe_2_O_4_ [62] or inverse spinels like CoFe_2_O_4_, NiFe_2_O_4_ [63, 64].

In biomedical contexts, spinel ferrites dominate due to their nanoscale synthesizability, chemical stability, high magnetization, and easy surface functionalization. Among these: (i) Magnetite (Fe_3_O_4_) and maghemite (γ‐Fe_2_O_3_) are the most biocompatible and have already been approved for limited clinical use as MRI contrast agents and drug delivery carriers [65, 66]. (ii) Cobalt ferrite (CoFe_2_O_4_) exhibits high magnetic anisotropy and coercivity, suitable for magnetic hyperthermia and magneto‐mechanical therapy [67]. (iii) Nickel ferrite (NiFe_2_O_4_) and manganese ferrite (MnFe_2_O_4_) display intermediate magnetic properties with enhanced stability, ideal for controlled heating and targeted imaging [68, 69, 70]. (iv) Zinc ferrite (ZnFe_2_O_4_), being a normal spinel with low anisotropy, offers soft magnetic characteristics and excellent biocompatibility, making it attractive for biosensing and photothermal therapy [71, 72, 73]. The ability to tailor magnetic properties through cation substitution such as Co–Zn [74], Ni–Zn [75], Mn–Zn [76], or rare‐earth‐doped spinels [77, 78, 79] provides a versatile toolkit to engineer ferrite powders for specific biomedical roles.

#### 3.1.2 Hexagonal Ferrites (MFe_12_O_19_)

Hexagonal ferrites, commonly known as M‐type ferrites, possess a magnetoplumbite structure characterized by alternating spinel (S) and hexagonal (R) blocks. The general formula is MFe_12_O_19_, where M is a divalent ion such as Ba^2+^ or Sr^2+^. These materials exhibit high coercivity, chemical robustness, and strong magnetocrystalline anisotropy [80, 81].

In recent years, hexaferrites have garnered attention in biomedicine due to their stable magnetic response, low eddy current losses, and compositional tunability [59]. Although traditionally used in permanent magnets and microwave devices, nanosized hexaferrite powders have shown potential in magnetic hyperthermia, biosensing, and antibacterial coatings. Substitutions with rare‐earth elements like La^3+^, Nd^3+^, Sm^3+^, and Gd^3+^, which can significantly reduce particle size, improve saturation magnetization, and enhance biocompatibility, are attributes favorable for biomedical integration [18, 82]. Moreover, their resistance to corrosion and oxidative degradation ensures safety under physiological conditions.

#### 3.1.3 Garnet Ferrites (R_3_Fe_5_O_12_)

Garnet ferrites, or yttrium iron garnets (YIG) and their rare‐earth analogues (R_3_Fe_5_O_12_), feature a complex cubic structure with multiple Fe^3+^ sublattices contributing to their ferrimagnetic ordering [83]. They exhibit low dielectric loss, moderate saturation magnetization, and optical transparency in the infrared region, which make them candidates for bio‐opto‐magnetic applications. In the biomedical domain, garnet ferrites have recently been explored for multimodal imaging and magneto‐optical biosensors, as their magneto‐optical coupling can enable real‐time monitoring of biological interactions [84]. Moreover, their compositional flexibility allows tuning of magnetic and optical responses simultaneously. For instance, Eu^3+^ or Gd^3+^ substitutions improve luminescence and MRI contrast performance, thus supporting the emerging field of theranostics (therapy + diagnostics) [85].

**FIGURE 4:**
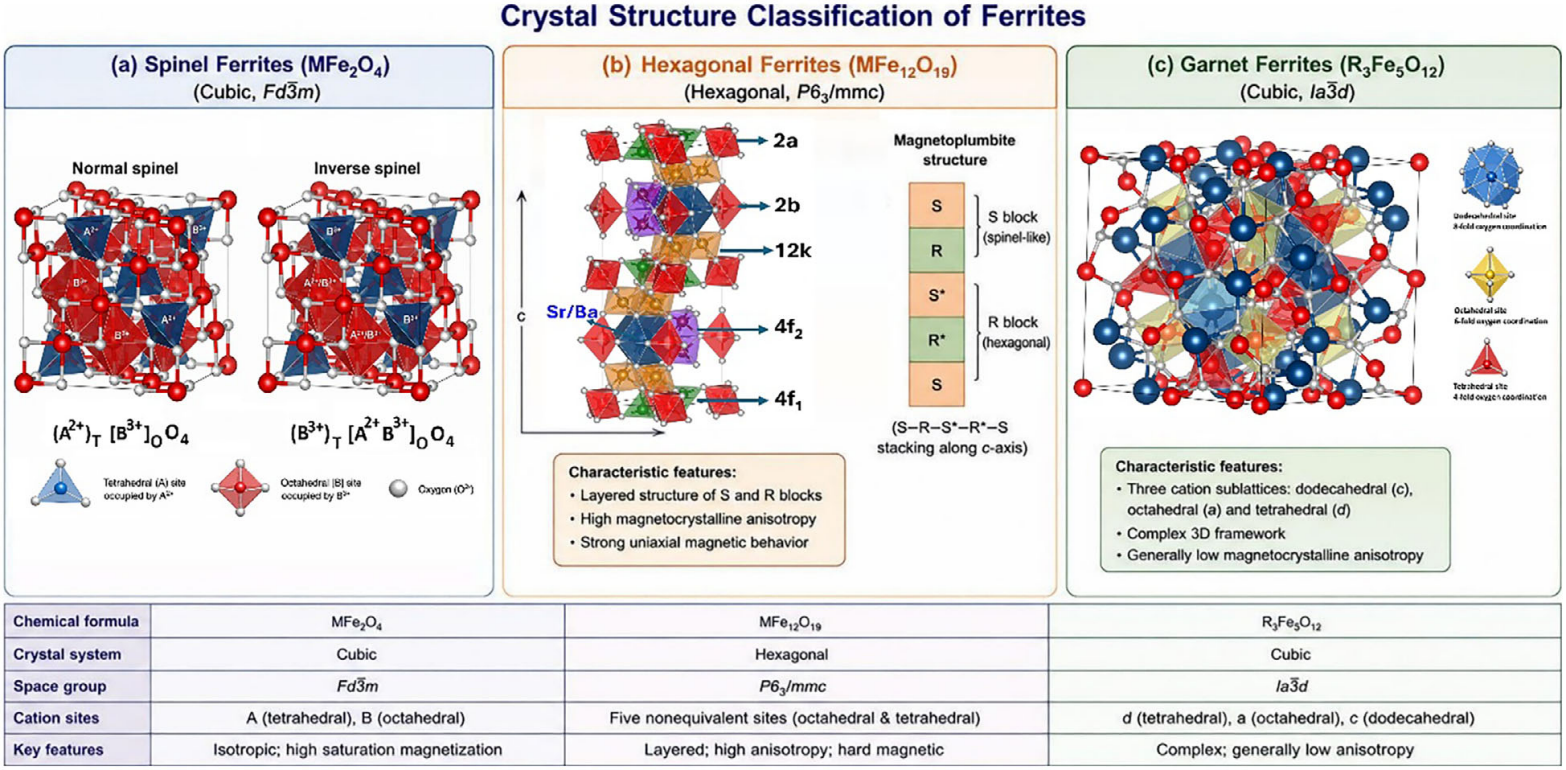
Schematic representation of the crystal structures of major ferrite families: (a) spinel ferrites (MFe_2_O_4_) with tetrahedral (A) and octahedral (B) cation sites, (b) hexagonal ferrites (MFe_12_O_19_) exhibiting magnetoplumbite layered structure with S and R blocks, and (c) garnet ferrites (R_3_Fe_5_O_12_) with complex cubic structure consisting of dodecahedral, octahedral, and tetrahedral sublattices.

To facilitate a clear understanding of the structural diversity among ferrite materials, a schematic comparison of the principal crystallographic frameworks is presented in Figure 4. The figure highlights the distinct atomic arrangements and cation distributions in spinel, hexagonal, and garnet ferrites, which fundamentally govern their magnetic and physicochemical properties.

### 3.2 Magnetic Classification of Ferrites

Ferrite materials can also be classified according to their magnetic behavior, which governs their interaction with external magnetic fields and biological systems.

#### 3.2.1 Hard Ferrites

Hard ferrites possess high coercivity and remanent magnetization, maintaining a permanent magnetic moment even after the external field is removed. Hexagonal ferrites such as BaFe_12_O_19_ and SrFe_12_O_19_ belong to this category [86, 87]. In biomedical applications, their strong magnetism enables remote magnetic actuation, magnetic bioseparation, and localized field stimulation, although care must be taken to prevent agglomeration and ensure biocompatibility through surface modification.

#### 3.2.2 Soft Ferrites

Soft ferrites exhibit low coercivity and can easily magnetize and demagnetize under alternating magnetic fields. Spinel ferrites such as MnFe_2_O_4_, ZnFe_2_O_4_, and NiFe_2_O_4_ are typical soft magnetic materials. These are particularly valuable in magnetic hyperthermia and MRI contrast enhancement, where rapid magnetic response and low hysteresis losses are required [88, 89]. Their biocompatibility and ease of synthesis at the nanoscale make them preferred candidates for in vivo applications.

#### 3.2.3 Superparamagnetic Ferrites

When the particle size of ferrites is reduced below a critical dimension (typically <20 nm), they exhibit superparamagnetism, where each nanoparticle behaves as a single magnetic domain that can randomly flip under thermal agitation in the absence of a magnetic field. This property eliminates remanence and coercivity, preventing agglomeration and magnetic clustering in biological systems [90]. Superparamagnetic ferrites, such as Fe_3_O_4_ or MnFe_2_O_4_ nanoparticles, are ideal for drug delivery, biosensing, and MRI since they respond strongly to magnetic fields while remaining nonmagnetic when the field is removed. Their stability in colloidal suspension and ability to generate localized heat under alternating magnetic fields make them crucial in targeted hyperthermia therapy [4, 91, 92].

### 3.3 Chemical and Compositional Classification

**FIGURE 5:**
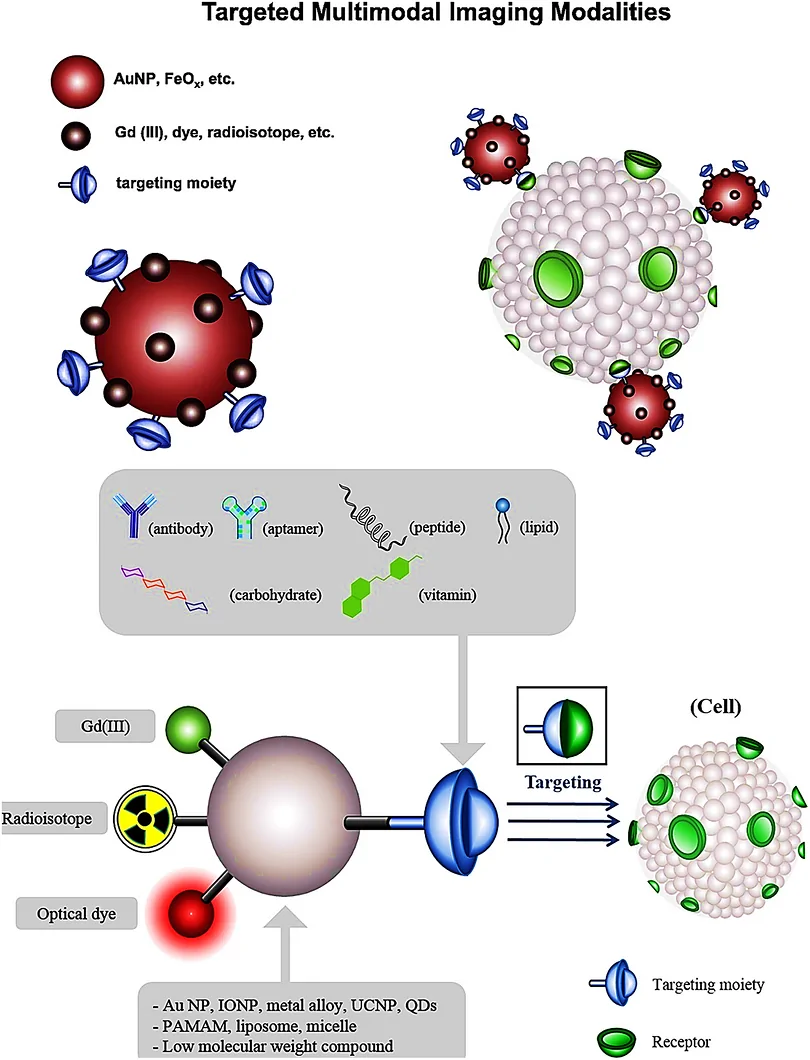
Ferrite‐based targeted multimodal imaging modalities. Reproduced with permission [93]. Copyright 2014, published by Elsevier.

The performance of ferrite materials in biomedical systems is intimately linked to their chemical composition and cationic configuration. Figure 5 is a schematic illustration of targeted multimodal imaging probes. Figure 5 demonstrates the design strategy for creating multifunctional imaging agents. It shows how various imaging modalities, such as magnetic resonance imaging (using Gd(III)), nuclear imaging (using radioisotopes), and optical imaging (using dyes), can be integrated onto a single nanoparticle carrier platform (e.g., AuNPs, liposomes, and micelles). To achieve site‐specific delivery, these multimodal probes are functionalized with targeting moieties like antibodies, peptides, or aptamers. The bottom section depicts the mechanism where the targeting moiety binds to specific receptors on a target cell, enabling the precise accumulation and simultaneous imaging of the target tissue using multiple complementary techniques [93].

The following compositional classes provide insight into how substitutional chemistry influences functionality:

#### 3.3.1 Simple Ferrites

These include stoichiometric ferrites like Fe_3_O_4_, CoFe_2_O_4_, NiFe_2_O_4_, and ZnFe_2_O_4_. Each displays distinct magnetic and biological behavior. For example, Fe_3_O_4_ is the most widely used ferrite in clinical diagnostics due to its strong magnetization and low toxicity. ZnFe_2_O_4_ exhibits soft magnetism and superior biocompatibility, while CoFe_2_O_4_ and NiFe_2_O_4_ provide higher magnetic anisotropy suitable for hyperthermia applications.

#### 3.3.2 Mixed and Substituted Ferrites

Substitutional doping of ferrites offers an effective strategy to modulate their properties. Partial replacement of Fe^3+^ or the divalent metal ion with other cations such as Mn^2+^, Cu^2+^, Mg^2+^, or rare‐earth ions can alter saturation magnetization, Curie temperature, and surface reactivity [94, 95, 96]. The Co–Zn ferrites (Co_1‐x_Zn_x_Fe_2_O_4_) combine the strong magnetism of cobalt ferrite with the softness and biocompatibility of zinc ferrite. Ni–Zn ferrites (Ni_1‐x_Zn_x_Fe_2_O_4_) exhibit controlled coercivity and enhanced heating efficiency for magnetic hyperthermia [97, 98]. Rare‐earth‐doped ferrites (such as GdFe_2_O_4_, La_0.5_Nd_0.5_FeO_3_ composites) show improved magnetic response, luminescence, and potential for multimodal imaging [99, 100, 101]. Such compositional flexibility enables the fine‐tuning of ferrite powders for specific biomedical functions, aligning magnetic behavior, toxicity, and degradation profiles with therapeutic requirements.

#### 3.3.3 Composite and Hybrid Ferrite Systems

Beyond single‐phase ferrites, hybrid ferrite systems have gained increasing attention due to their ability to integrate magnetic functionality with complementary physicochemical properties. These systems include ferrite–polymer, ferrite–carbon, ferrite–silica, and ferrite–metal hybrids, engineered either as composites or core–shell architectures [100, 101, 102, 103]. Core–shell ferrites offer distinct advantages, such as enhanced chemical stability, reduced ion leaching, and improved biocompatibility, while preserving magnetic performance [104, 105]. For instance, silica or polymer shells suppress surface oxidation and mitigate protein corona formation, whereas carbon shells enhance photothermal synergy. However, core–shell engineering introduces trade‐offs, including reduced saturation magnetization due to magnetic dilution and potential interfacial strain effects. From a powder‐processing standpoint, controlling shell thickness, interfacial bonding, and phase compatibility is critical to balancing magnetic efficiency and biological functionality. Consequently, hybrid ferrite systems represent a versatile platform for tailoring multifunctional performance toward translational biomedical applications.

In pursuit of multifunctionality, ferrites are increasingly integrated with other materials, forming hybrid or core–shell architectures. The composite and hybrid ferrites include (i) Ferrite@Silica or Ferrite@Polymer: Enhances colloidal stability, prevents oxidation, and enables biomolecule attachment [103]. (ii) Ferrite@Gold (Au): Combines magnetic manipulation with optical plasmonic properties for dual‐mode imaging [106, 107]. (iii) Ferrite@Carbon or Ferrite–Graphene hybrids: Improves electrical conductivity and biocompatibility, expanding use in biosensing and tissue stimulation [108, 109]. These hybrid designs merge magnetic responsiveness with optical, catalytic, or biological functionalities, that critical for theranostic and regenerative applications.

While ferrite‐based nanomaterials have demonstrated significant potential in biomedical applications due to their unique magnetic properties, it is important to benchmark their performance against other emerging material systems. Layered double hydroxides (LDHs), as discussed by Luo et al. [110], have gained attention in regenerative nanomedicine owing to their excellent biocompatibility, tunable ion release, and layered structure that facilitates drug intercalation. In contrast to ferrites, LDHs exhibit superior biodegradability and ion‐mediated therapeutic effects; however, they lack intrinsic magnetic functionality, limiting their applicability in magnetically guided therapies and hyperthermia. Similarly, zinc‐based biomaterials, highlighted by Yuan et al. [111], have shown promising results in additive manufacturing and bone tissue engineering due to their biodegradability, osteogenic potential, and controlled Zn^2+^ ion release. Compared to ferrites, zinc‐based systems offer enhanced bioactivity and scaffold integration but do not provide magnetic responsiveness, which is critical for applications such as targeted drug delivery and MRI contrast enhancement. These comparisons indicate that while ferrites uniquely enable magnetically driven biomedical functions, their relatively limited biodegradability and ion‐release‐based bioactivity remain challenges. Therefore, hybrid material strategies combining ferrites with LDHs or zinc‐based systems may offer a promising pathway toward multifunctional biomedical platforms

### 3.4 Functional Classification Based on Biomedical Applications

The functional versatility of ferrite materials enables their integration across multiple domains of biomedicine, ranging from diagnostic imaging to therapeutic and regenerative applications. Their physicochemical tunability, biocompatibility, and surface modification potential make them ideal for developing next‐generation multifunctional nanoplatforms [112, 113, 114]. Depending on the targeted biomedical function, ferrites can be categorized into distinct classes based on their primary roles, as summarized in Table 1.

**TABLE 1:** Functional classification of ferrite‐based materials in biomedical applications.

Sr. No.	Category	Representative ferrite system	Key functional role	Refs.
1.	Diagnostic agents (MRI, biosensing)	Fe_3_O_4_, MnFe_2_O_4_, ZnFe_2_O_4_	High magnetic moment, T_1_/T_2_ contrast enhancement, sensitive magnetic biosensing	[4, 36]
2.	Therapeutic agents (hyperthermia, drug delivery)	CoFe_2_O_4_, Ni–ZnFe_2_O_4_, rare‐earth doped ferrites	Controlled heat generation, magnetic guidance, triggered drug release	[115, 116]
3.	Antimicrobial coatings & wound healing	CuFe_2_O_4_, ZnFe_2_O_4_	ROS generation, bacterial membrane disruption, biofilm inhibition	[47, 117]
4.	Regenerative medicine & tissue engineering	BaFe_12_O_19_, SrFe_12_O_19_, MnFe_2_O_4_ composites	Magnetic stimulation of cells, scaffold reinforcement, osteogenic promotion	[118, 119, 120]
5.	Theranostic systems (combined therapy and imaging)	Gd‐doped or Eu‐doped spinel ferrites, Ferrite–Au hybrids	Dual‐mode imaging and targeted therapy	[121, 122]

Recent advances in magnetic tissue engineering have increasingly focused on the development of 3D‐printed composite scaffolds incorporating ferrite nanoparticles to achieve both structural support and functional activity. In this context, Kanwar et al. [123] demonstrated the fabrication of magnetic polymer–ferrite composite scaffolds using additive manufacturing techniques, enabling precise control over scaffold architecture, porosity, and mechanical integrity. These scaffolds exhibited enhanced mechanical properties suitable for load‐bearing applications, while the incorporation of ferrite nanoparticles imparted magnetic responsiveness, enabling external field stimulation. Importantly, such multifunctional scaffolds can simultaneously support tissue regeneration and localized magnetic hyperthermia, offering a dual therapeutic approach for applications such as bone cancer treatment. The study highlights that scaffold performance is governed not only by material composition but also by fabrication parameters, including printing resolution, layer orientation, and nanoparticle dispersion [124, 125]. This integrated approach underscores the potential of ferrite‐based composites in bridging structural and functional requirements for next‐generation biomedical implants. Beyond primary tumor therapy, ferrite‐based nanomaterials are increasingly being explored for the treatment of bone metastasis, where both tumor suppression and bone regeneration are required. As reported by Hao et al. [126], multifunctional nanoplatforms integrating magnetic nanoparticles with osteotropic components can enable targeted delivery to bone tissue, combined with localized hyperthermia and therapeutic agent release. Such systems are particularly advantageous in addressing the dual challenge of inhibiting metastatic tumor growth while promoting bone repair. The magnetic responsiveness of ferrites further enables site‐specific accumulation under external fields, enhancing therapeutic precision [127]. These findings highlight the potential of ferrite‐based materials in bone‐targeted theranostics, extending their application beyond conventional cancer treatments.

The above classification highlights how compositional and structural modifications in ferrites directly dictate their biomedical functionality. For instance, Fe_3_O_4_ and MnFe_2_O_4_ nanoparticles exhibit superior soft magnetic behavior suitable for MRI and biosensing [4, 36], whereas CoFe_2_O_4_ and Ni–ZnFe_2_O_4_ with higher coercivity and anisotropy are advantageous for magnetic hyperthermia and drug delivery [115, 116]. Similarly, Cu‐ and Zn‐containing ferrites impart inherent antimicrobial activity through reactive oxygen species (ROS) generation [47, 117], while hexaferrites such as BaFe_12_O_19_ and SrFe_12_O_19_ have recently gained attention for magnetic stimulation in tissue engineering [118, 119, 120]. The development of Gd‐ and Eu‐doped ferrites further bridges the gap between imaging and therapy, enabling efficient theranostic applications [121, 122]. Overall, this functional classification demonstrates that rational design and compositional tuning of ferrite nanostructures can yield application‐specific materials that combine multiple therapeutic, diagnostic, and regenerative functions, a crucial step toward personalized and multimodal biomedical technologies.

## 4 Structural and Compositional Tailoring of Ferrites for Biomedical Performance

The multifunctional behavior of ferrite materials in biomedical applications stems from their remarkable structural tunability and compositional versatility. By modifying cation distribution, crystal morphology, particle size, surface chemistry, and doping composition, the physicochemical and magnetic properties of ferrites can be precisely engineered to suit specific biomedical requirements. Such tailoring strategies are essential for optimizing critical parameters including magnetic response, biocompatibility, colloidal stability, drug loading, and targeting efficiency.

**FIGURE 6:**
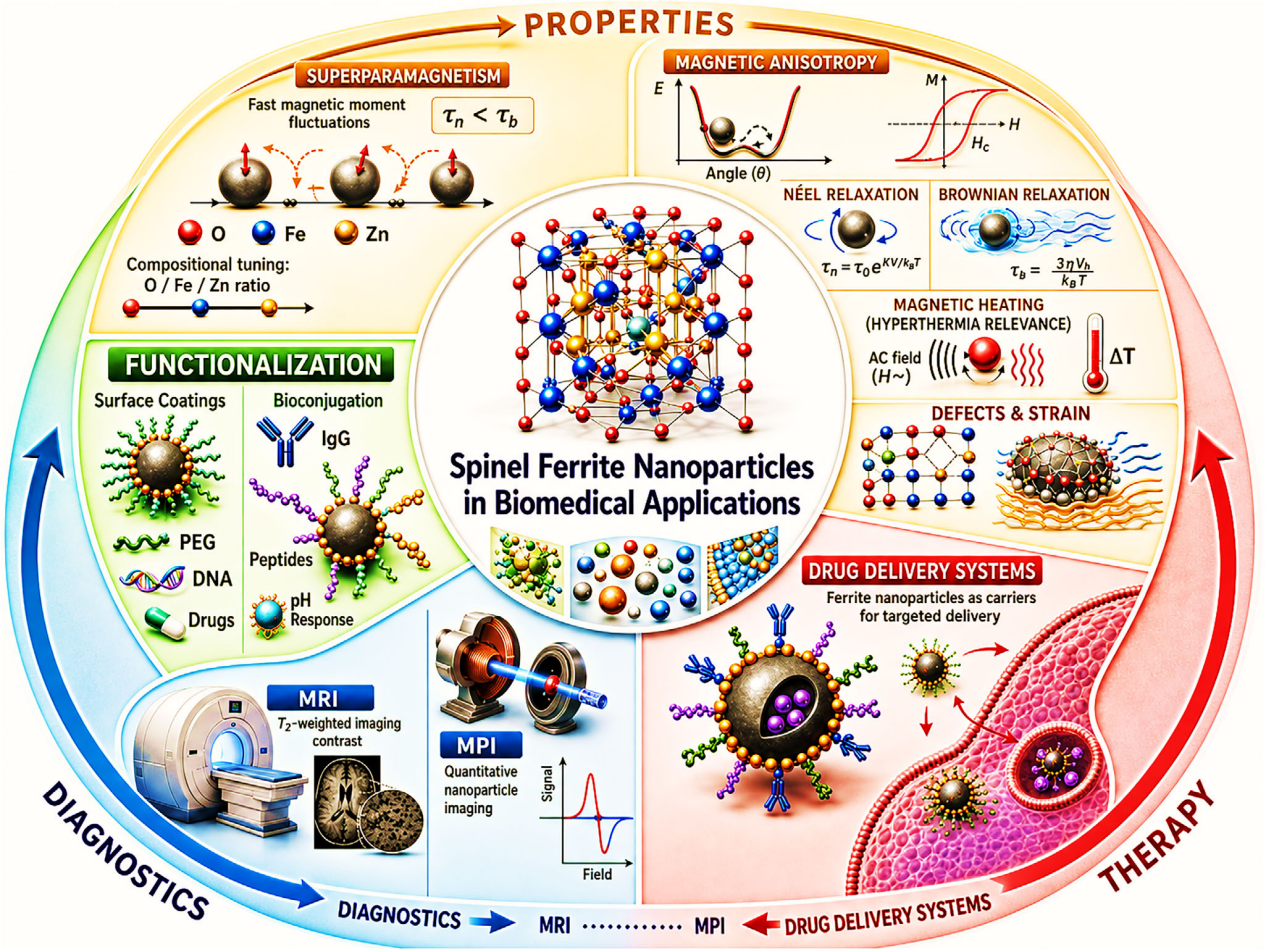
Schematic overview of spinel ferrite nanoparticles in biomedical applications, illustrating how compositional tuning, superparamagnetism, magnetic anisotropy, relaxation behavior, defect/strain engineering, and surface functionalization govern their diagnostic and therapeutic performance. The figure highlights the structure–property–function relationship underlying MRI and MPI diagnostics, targeted drug delivery, and other ferrite‐enabled theranostic applications.

Figure 6 provides a conceptual overview of how structural and compositional tailoring of spinel ferrite nanoparticles governs their biomedical performance. The schematic highlights the role of cation doping, defect modulation, and nanoscale magnetic parameters such as saturation magnetization, coercivity, and Néel–Brownian relaxation in dictating functional outcomes. By linking compositional tuning with diagnostic (MRI, MPI) and therapeutic (magnetic hyperthermia, drug delivery, magneto‐mechanical therapy) applications, the figure illustrates the structure–property–function relationships that underpin rational ferrite design. Figure 6 further demonstrates that multifunctional performance in ferrite‐based systems is achieved through the integration of structural, magnetic, and surface properties. The ability to simultaneously control parameters such as saturation magnetization, surface chemistry, and particle size enables the development of systems capable of combined imaging, therapy, and drug delivery. This highlights the importance of multifunctional design strategies for next‐generation biomedical applications. This integrative representation sets the framework for the quantitative design rules, defect engineering strategies, and functional optimization approaches discussed in the following sections.

### 4.1 Structural Framework and Cation Distribution

Ferrites generally adopt three fundamental crystallographic structures, such as spinel (AB_2_O_4_), hexagonal (M‐type, BaFe_12_O_19_ or SrFe_12_O_19_), and garnet (A_3_B_5_O_12_) types, and each exhibits distinct cationic distributions between tetrahedral (A) and octahedral (B) sites. The magnetic and electronic behavior of ferrites is largely governed by superexchange interactions between Fe^3+^ and substituted cations occupying these sites [128]. In spinel ferrites, variations in A–B site occupancy significantly impact the magnetocrystalline anisotropy, saturation magnetization (*M*_s_), and coercivity (*H*_c_). The inverse spinels such as Fe_3_O_4_ and CoFe_2_O_4_ exhibit high magnetic moments and moderate anisotropy, making them ideal candidates for magnetic hyperthermia and MRI contrast agents [129]. Conversely, the normal spinels such as ZnFe_2_O_4_ show low coercivity and superparamagnetic behavior at nanoscale dimensions, which are favorable for drug delivery and biosensing applications where rapid magnetic response and low remanence are required [130, 131].

Moreover, the control of cation distribution via doping by replacing Fe^3+^ with Mn^2+^, Ni^2+^, or rare‐earth ions, that allows fine‐tuning of electronic spin interactions and relaxation times, thereby enhancing contrast efficiency in MRI or specific absorption rates (SAR) in hyperthermia. Such atomic‐level engineering of cation arrangement has emerged as a cornerstone in designing biomedical ferrites with predictable magnetic responses and biocompatibility [132]. While magnetic anisotropy and saturation magnetization are key parameters governing heating efficiency, the role of frequency tunability and adaptive magnetic response is increasingly recognized as a critical factor in biomedical applications. Conventional ferrite systems are typically optimized for fixed frequency ranges; however, physiological environments often require dynamic adjustment of operating conditions to achieve optimal performance. Recent work by Zhang et al. [133] introduces frequency‐adjustable heterojunction‐based magnetic systems, where interfacial coupling between distinct magnetic phases enables tunable relaxation dynamics under varying excitation frequencies. Such adaptive systems allow modulation of energy dissipation mechanisms, leading to improved control over specific absorption rate (SAR) and enhanced responsiveness for applications such as magnetic hyperthermia and biosensing. This approach highlights a paradigm shift from static material optimization toward externally tunable and environment‐responsive magnetic platforms, enabling more precise and efficient biomedical interventions.

### 4.2 Particle Size and Morphological Control

Particle size and shape strongly influence both magnetic and biological behaviors of ferrites. As particle size decreases below the single‐domain limit (typically 10–30 nm), ferrites transition from ferromagnetic to superparamagnetic behavior, eliminating magnetic aggregation and residual magnetization [90]. This is particularly advantageous for in vivo biomedical applications, where reversible magnetic response ensures safe circulation and clearance post‐treatment. The morphology, which includes spherical, cubic, rod‐like, or flower‐like nanoparticles, affects surface area, cellular uptake, and magnetic heating efficiency [134]. The cubic and flower‐shaped Fe_3_O_4_ or CoFe_2_O_4_ nanoparticles often exhibit enhanced SAR values due to higher magnetic anisotropy, whereas spherical ZnFe_2_O_4_ or MnFe_2_O_4_ nanoparticles promote improved colloidal stability and biocompatibility is tabulated in Table 2. Tailored synthesis routes such as sol–gel, co‐precipitation, hydrothermal, and microemulsion methods allow precise control over particle size distribution and morphology. Surface passivation using organic ligands or polymers such as citric acid, PEG, and dextran further prevents agglomeration, stabilizes colloidal suspensions, and enhances biocompatibility without compromising magnetic properties.

**TABLE 2:** Various morphological features such as shape anisotropy, magnetocrystalline anisotropy, SAR values, colloidal stability, and biocompatibility of ferrite nanoparticles.

Feature	Cubic/Flower‐shaped Fe_3_O_4_ or CoFe_2_O_4_ [135, 136, 137, 138]	Spherical ZnFe_2_O_4_ or MnFe_2_O_4_ [139, 140, 141, 142]
Shape anisotropy	Higher due to nonspherical shape.	Lower (isotropic).
Magnetocrystalline anisotropy	CoFe_2_O_4_ has high magnetocrystalline anisotropy, which contributes to high SAR.	MnFe_2_O_4_ and ZnFe_2_O_4_ generally have lower anisotropy compared to CoFe_2_O_4_.
SAR values	Enhanced, making them highly efficient for magnetic hyperthermia treatments.	Generally lower, but can be optimized by controlling size/doping or by using core‐shell structures.
Colloidal stability	May require specific surface functionalization to prevent aggregation due to strong magnetic interactions.	Generally good stability, especially in aqueous solutions with proper coating.
Biocompatibility	Fe_3_O_4_ is widely studied for its good biocompatibility, while CoFe_2_O_4_ can have higher toxicity at certain concentrations, requiring careful surface functionalization.	Known for excellent biocompatibility and minimal toxicity, making them favorable candidates for cancer theranostics.

### 4.3 Cation Substitution and Doping Strategies

Cation substitution is one of the most powerful approaches to engineer the functional properties of ferrites. By introducing specific dopant ions into the A or B sites, both magnetic and electronic characteristics can be tuned to optimize biomedical performance. (i) Transition metal dopants such as Mn^2+^, Ni^2+^, Cu^2+^, and Zn^2+^ that modify magnetic anisotropy and coercivity; which offer higher magnetization, suitable for hyperthermia and MRI, while Zn^2+^ substitution reduces magnetic interactions, improving drug delivery performance [143, 144]. (ii) Rare‐earth ions such as Gd^3+^, Eu^3+^, Nd^3+^, and Sm^3+^, that introduce 4f magnetic moments, improving magnetic relaxation and enabling dual‐mode imaging such as MRI/fluorescence or theranostic applications. Gd^3+^ doping in Fe_3_O_4_ or CoFe_2_O_4_ enhances T_1_ contrast, while Eu^3+^ doping imparts luminescence for optical imaging [145, 146, 147]. (iii) The nonmagnetic or biocompatibility‐enhancing dopants (Ca^2+^, Mg^2+^, and Sr^2+^) which reduce magnetic hardness and improve cellular compatibility. MgFe_2_O_4_ nanoparticles that exhibit excellent biocompatibility and thermal stability suitable for drug encapsulation [148, 149]. Such dopant engineering provides a rational route to design application‐specific ferrites with high‐moment CoFe_2_O_4_ for hyperthermia, luminescent EuFe_2_O_4_ for bioimaging, or antibacterial CuFe_2_O_4_ for wound healing. The combination of multiple dopants like Zn–Gd or Mn–Eu co‐doped systems can further produce synergistic effects for multifunctional biomedical performance.

### 4.4 Surface Modification and Functionalization

For biomedical deployment, ferrite nanoparticles must be surface‐engineered to achieve stability in physiological environments, biocompatibility, and targeted functionality. Bare ferrite surfaces often exhibit high surface energy, leading to aggregation and nonspecific protein adsorption. Hence, surface functionalization serves as a crucial step to ensure biological safety and enhance targeted interaction with cells or tissues [44, 150, 151]. The polymer coatings (PEG, PVP, dextran, chitosan) that provide steric stabilization, hydrophilicity, and stealth characteristics that prevent immune recognition. The PEGylated Fe_3_O_4_ nanoparticles are among the most widely used MRI agents due to their excellent in vivo circulation time [152, 153]. The silica and carbon shells improved chemical stability and enabled further bio‐conjugation with antibodies, enzymes, or drugs [154]. The Fe_3_O_4_@SiO_2_ core–shell nanostructures have been utilized for biosensing and magnetic separation of biomolecules. The Biofunctional ligands, like folic acid, antibodies, and peptides, introduce targeting ability for specific cancer cells or receptors, enhancing drug delivery precision and minimizing side effects [155]. The use of plant extracts, amino acids, or polysaccharides as both reducing and capping agents reduces toxicity while introducing biocompatible surface functionality [156].

Beyond conventional surface coatings, recent advances emphasize the importance of biomimetic interfacial design, where nanoparticle surfaces are engineered to emulate biological structures and functions. As highlighted by Zheng et al. [157], such bioinspired interfaces enable precise control over protein adsorption, cellular recognition, and adhesion behavior, which are critical determinants of in vivo performance. In particular, the formation of the protein corona a dynamic layer of biomolecules adsorbed onto nanoparticle surfaces plays a decisive role in modulating biological identity and cellular uptake. Biomimetic strategies, including zwitterionic coatings, lipid‐mimetic layers, and peptide‐functionalized surfaces, can regulate corona composition and minimize nonspecific interactions. This leads to improved biocompatibility, reduced immune response, and enhanced targeting efficiency. These insights establish that interfacial design is not merely a protective strategy but a functional and dynamic component governing nanoparticle–biological system interactions. Overall, surface chemistry acts as the interface between the magnetic core and the biological system, dictating cellular uptake, biodistribution, and therapeutic efficacy. Optimizing this interface remains one of the most critical design parameters for clinical translation of ferrite nanomaterials.

### 4.5 Phase Engineering and Composite Integration

Beyond conventional spinel or hexaferrite phases, heterostructured and composite ferrite systems have gained increasing attention for their synergistic biomedical functionalities [158]. Coupling ferrites with other materials, such as noble metals (Au, Ag), polymers, or carbon derivatives (graphene, CNTs), that can simultaneously enhance magnetism, conductivity, and biocompatibility. The Fe_3_O_4_–Au hybrid nanoparticles combine superparamagnetic behavior with plasmonic properties, enabling simultaneous photothermal therapy and MRI imaging [159, 160]. Ferrite–hydroxyapatite composites improve osteogenic differentiation in bone tissue engineering through magnetically stimulated bioactivity. Core–shell ferrite structures such as CoFe_2_O_4_@MnFe_2_O_4_ optimize SAR values and magnetic relaxation, crucial for hyperthermia efficiency [159]. Phase engineering strategies also allow fine control over magnetic anisotropy and exchange coupling, producing materials that balance high magnetization with biological safety that a key requirement for multifunctional biomedical nanoplatforms.

### 4.6 Correlation Between Structure, Magnetism, and Biological Response

A fundamental understanding of the correlation between structural characteristics and biological outcomes is essential for rational ferrite design. Smaller superparamagnetic nanoparticles (<20 nm) typically exhibit higher cellular internalization and faster renal clearance, while larger or aggregated particles may show prolonged retention and potential cytotoxicity. Magnetic parameters such as saturation magnetization (*M*_s_) and anisotropy constant (*K*) determine heating efficiency in magnetic hyperthermia. Similarly, surface charge (zeta potential) and hydrodynamic diameter influence protein corona formation and biodistribution [161, 162].

While significant emphasis has been placed on enhancing heating efficiency through high specific absorption rate (SAR), effective magnetic hyperthermia also requires careful consideration of heat dissipation and spatial temperature distribution within biological tissues. Nonuniform heat generation can lead to localized overheating, potentially damaging healthy tissues, while insufficient heat propagation may reduce therapeutic efficacy. Recent advances have demonstrated that thermal conductivity engineering through composite design can address these challenges. In particular, Yang et al. [163] reported that incorporating ferrite nanoparticles into carbon nanotube (CNT)‐based hybrid systems significantly enhances thermal conductivity, enabling more uniform heat distribution and controlled thermal gradients. Such composite systems facilitate efficient heat transfer from localized nanoparticle heating sites to surrounding tissue, improving safety and treatment consistency. These findings highlight that optimizing hyperthermia performance requires not only maximizing heat generation but also controlling heat diffusion pathways, thereby integrating magnetic and thermal transport properties into a unified design strategy.

Recent studies reveal that cation composition and crystal defects also play a vital role in modulating reactive oxygen species (ROS) generation, which can be exploited for antibacterial or anticancer effects. Gupta et al. demonstrate that the manipulation of cation composition is a potent strategy for tuning the defect structure of metal oxides to enhance biomedical efficacy. By substituting Mg^2+^ ions, the study observed a widening of the band gap and a significant increase in the concentration of surface defects, specifically oxygen vacancies, compared to pure ZnO. These surface defects act as active sites that facilitate the generation of reactive oxygen species (ROS), including hydroxyl radicals, superoxide radicals, and singlet oxygen, even in the absence of light. The enhanced ROS generation resulting from this defect engineering played a critical role in biological interactions [164]. Hence, precise control over microstructural features such as grain size, crystallinity, defect density, and surface energy allows tailoring of ferrite materials for desired biological functionalities.

**FIGURE 7:**
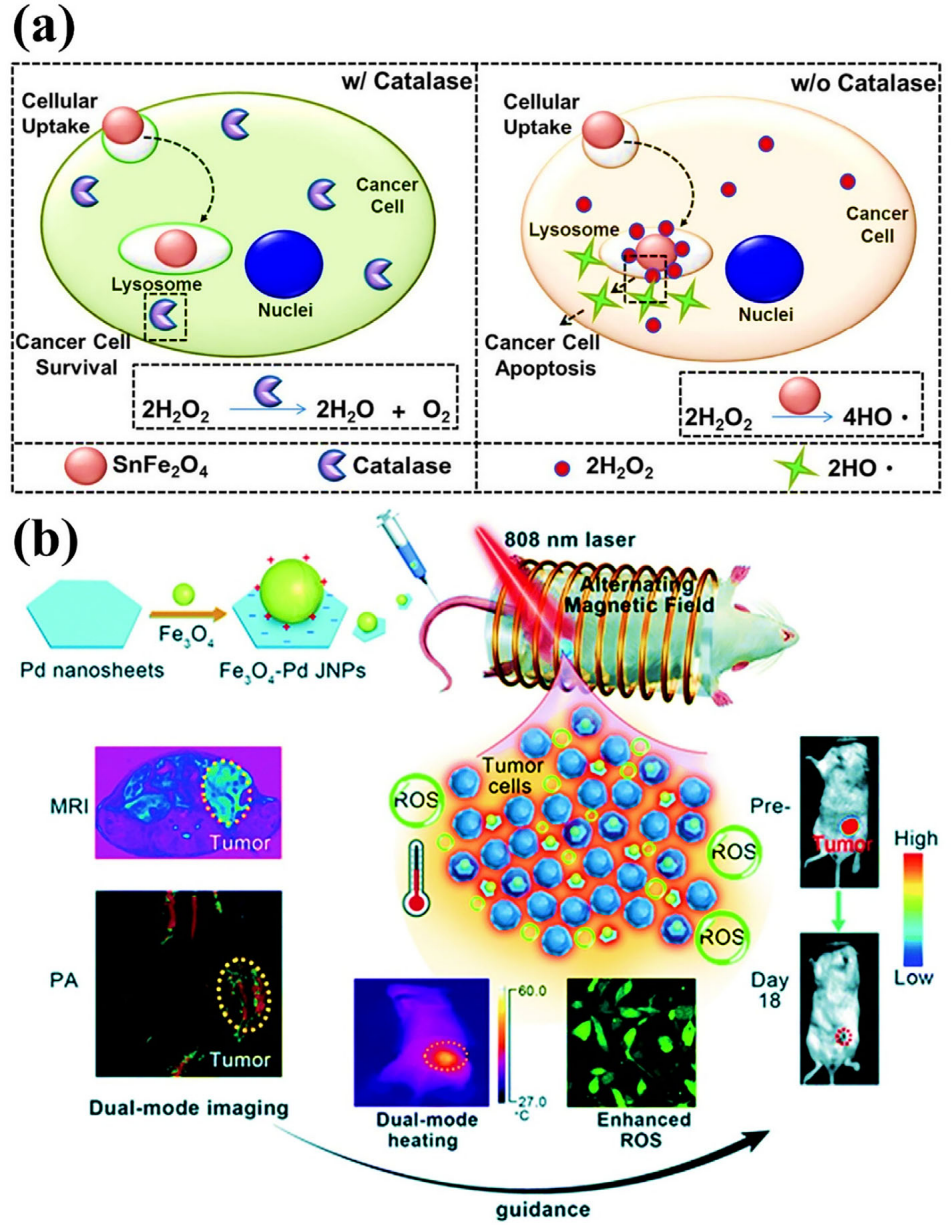
(a) SnFe_2_O_4_ nanocrystals: Cytotoxicity in cancer cells versus catalase‐mediated protection. (b) Schematic of Fe_3_O_4_–Pd Janus nanoparticles for MRI/PA‐guided hyperthermia and ROS‐mediated breast cancer therapy. (a) Reproduced with permission [165]. Copyright 2017, American Chemical Society. (b) Reproduced with permission [166]. Copyright 2019, Royal Society of Chemistry.

Figure 7a illustrate the intracellular mechanism of sonicated SnFe_2_O_4_ nanocrystals. Upon high cellular uptake, these nanocrystals function as H_2_O_2_‐specific redox catalysts, triggering the heterogeneous Fenton reaction to produce a surge of cytotoxic hydroxyl radicals. Crucially, the schematic validates this pathway by demonstrating that catalase, an enzyme that decomposes H_2_O_2_, completely inhibits this reaction, thereby neutralizing the cytotoxic effects and confirming the therapy's dependence on intracellular peroxide levels [165]. As depicted in Figure 7b, the Fe_3_O_4_–Pd nanoplatform leverages the outstanding magnetic properties of Fe_3_O_4_ and the NIR surface plasmon resonance of Pd to achieve a synergistic theranostic effect. The illustration highlights the mechanism wherein intravenous administration is followed by dual‐mode magnetic‐photo heating. This amplified hyperthermia, coupled with significantly enhanced ROS production under simultaneous AMF and NIR exposure, results in potent antineoplastic activity and high‐resolution monitoring via MRI/PA imaging [166].

The structural and compositional engineering of ferrites represents a versatile toolbox to design multifunctional materials for biomedical applications. Emerging approaches such as atomically precise doping, defect modulation, and surface‐selective functionalization are paving the way toward clinically viable ferrite nanoplatforms that integrate diagnosis, therapy, and tissue regeneration [167]. Nevertheless, future research must focus on establishing structure–property–bioactivity relationships through advanced characterization techniques (XPS, Mössbauer spectroscopy, in situ TEM) and computational modeling to predict biological interactions. Achieving reproducible, scalable, and biocompatible synthesis while retaining magnetic functionality remains a grand challenge for translation from laboratory to clinic.

### 4.7 Quantitative Design Guidance for Ferrite Nanoparticles

There were numerous studies reporting structural and compositional modifications of ferrite nanoparticles, but fewer efforts have consolidated these findings into actionable design criteria. From a powder‐engineering perspective, quantitative design rules linking particle size, magnetic properties, and surface chemistry to application‐specific performance are essential for translational success. For instance, MRI contrast efficiency is maximized using superparamagnetic ferrite nanoparticles with narrow size distributions and high saturation magnetization, whereas magnetic hyperthermia requires enhanced magnetic anisotropy and stable heating under viscous or immobilized conditions. Drug delivery and biosensing applications further impose constraints on surface charge, colloidal stability, and functional ligand density. Table 3 summarizes representative design targets reported across the literature, providing practical guidance for tailoring ferrite nanoparticle systems toward specific biomedical functions. Such quantitative frameworks facilitate rational material selection, reduce empirical trial‐and‐error, and align powder design with clinically relevant performance requirements.

**TABLE 3:** Quantitative design guidance for spinel ferrite nanoparticles in biomedical applications.

Application	Particle size (nm)	Ms (emu/g)	Hc/Anisotropy	Surface charge (mV)	Structural/Compositional design focus
MRI contrast (T_2_)	5–20	60–90	∼0 (SPM)	−10 to −30	High crystallinity, narrow size distribution, e.g., Zn‐substituted magnetite [34, 143, 168]
MRI contrast (T_1_/T_2_ dual)	3–10	40–70	∼0 (SPM)	−5 to −20	Ultrasmall cores e.g. ZnMn ferrite <6 nm, surface spin control for low r_2_/r_1_ [169, 170]
Magnetic hyperthermia (Brownian + Néel)	15–30	50–80	Moderate	−5 to +15	Shape anisotropy e.g. spherical CoFe_2_O_4_, controlled aggregation for dual relaxation [171, 172]​
Magnetic hyperthermia (viscosity‐independent)	20–40	55–85	High	−5 to +10	High anisotropy materials, Néel/hysteresis‐dominated [140, 171]​
Drug delivery (systemic)	20–80	40–70	Low–moderate	± 10–25	Long circulation via PEG, stealth coatings on spinel cores like ZnFe_2_O_4_ [34, 173]
Drug delivery (magnetically guided)	50–150	>60	Moderate–high	± 10–20	High magnetic moment per particle, such as NiZn ferrites with tuned Ms [105, 174]
Biosensing (magnetic separation)	10–50	60–90	Low	−20 to −40	Fast response, minimal aggregation in NiZn or CoZn spinels [174]
Biosensing (signal amplification)	5–20	50–80	Low	−15 to −35	High surface functional group density on small SPM particles [168, 173]
Magnetic tissue engineering (Mag‐TE)	30–200	>60	High	Variable	Mechanical actuation, scaffold integration with high‐Ms ferrites [72]
Magneto‐mechanical cell stimulation	50–300	>65	High	Neutral to −20	Torque generation under low fields using anisotropic Co‐based spinels [171]​
Photothermal–magnetic hybrid therapy	20–60	40–70	Moderate	−10 to −25	Core–shell or carbon‐coated ferrites (such as CoFe_2_O_4_ hybrids) [52]
ROS‐mediated therapy	5–15	30–60	Low	−20 to −40	Defect‐rich surfaces, ion release control in small NiZn ferrites [173, 175]
Gene delivery	20–100	40–65	Low	+15 to +35	Cationic surface on Zn/Co ferrites for DNA binding efficiency [34]
In vivo tracking/imaging probes	5–15	50–75	∼0 (SPM)	−10 to −30	High stability, minimal protein corona on ultrasmall SPIONs [169, 176]

Table 3 presents quantitative design guidelines optimized for spinel ferrite nanoparticles (MFe_2_O_4_) across diverse biomedical applications, specifying critical parameters like particle size, saturation magnetization (*M*s), coercivity/anisotropy (*H*c), and zeta potential for performance optimization. These ranges derive from structure‐property relationships in cubic spinel lattices, where cation substitution (like Co, Zn, and Mn) enables tailoring of superparamagnetism (SPM) for imaging, anisotropy for hyperthermia, and surface charge for biocompatibility. The comparative data presented in Table 3 provide key insights into the design principles governing ferrite‐based nanomaterials for biomedical applications. Specifically, materials exhibiting high saturation magnetization and optimized anisotropy are more suitable for hyperthermia, whereas smaller particle sizes and appropriate surface coatings are critical for achieving high MRI relaxivity and biocompatibility. These observations indicate that application‐specific optimization is essential, as no single material configuration simultaneously maximizes all performance metrics. Instead, rational design should focus on tailoring composition, size, and surface functionality based on the intended clinical application. The Table 3 serves as a practical reference for researchers synthesizing ferrites via sol–gel methods, facilitating reproducible translation from lab to clinic by linking composition to application‐specific metrics like SAR efficiency and circulation half‐life.

#### 4.7.1 Advanced Structural Engineering and Predictive Design of Ferrite Nanomaterials

As structural and compositional tailoring of ferrite nanoparticles has been widely explored, the lack of consolidated quantitative design rules remains a key barrier to rational material selection. To bridge this gap, Table 3 summarizes representative target ranges of particle size, magnetic properties, and surface charge required for major biomedical applications. These design windows highlight how distinct applications impose fundamentally different constraints, for example, SPM behavior and ultrasmall size for MRI contrast, enhanced magnetic anisotropy for hyperthermia, and controlled surface charge for biosensing and drug delivery. Importantly, these parameters must be optimized simultaneously rather than independently, as deviations often lead to aggregation, reduced heating efficiency, or unpredictable biological interactions [177, 178, 179]. Establishing such quantitative guidance transforms ferrite nanoparticle development from empirical optimization to a structure–property function guided design paradigm, aligning powder engineering strategies with translational performance requirements [180, 181].

#### 4.7.2 Defect Engineering: Role of Point Defects in Biomedical Performance

Defect engineering has emerged as a powerful yet underutilized strategy for tuning the biomedical performance of ferrite nanoparticles. Point defects such as oxygen vacancies, cation vacancies, and mixed‐valence states (Fe^2+^/Fe^3+^) significantly influence magnetic anisotropy, saturation magnetization, and surface reactivity. Oxygen vacancies, in particular, enhance local magnetic disorder and can increase Néel relaxation losses, thereby improving hyperthermia efficiency. Simultaneously, defect‐rich surfaces may promote reactive oxygen species (ROS) generation, which can be beneficial for synergistic cancer therapies but detrimental if uncontrolled [178, 181]. From a powder‐processing perspective, defect density can be modulated through synthesis atmosphere, annealing temperature, and quenching rate. However, excessive defect concentrations may accelerate chemical degradation and ion leaching. Therefore, controlled defect engineering represents a critical balance between enhanced functionality and long‐term biological stability [182, 183].

#### 4.7.3 Lattice Strain Effects and Strain‐Induced Property Modulation

Lattice strain arising from size confinement, dopant incorporation, or core–shell interfaces plays a decisive role in governing ferrite nanoparticle properties. Compressive or tensile strain alters cation distribution among tetrahedral and octahedral sites, thereby modifying magnetic exchange interactions and anisotropy constants. Strain‐induced distortion has been shown to enhance magnetic hardness in doped ferrites, directly impacting hyperthermia and magneto‐mechanical actuation efficiency [183]. In core–shell architectures, interfacial strain can either stabilize magnetic moments or induce spin canting, depending on lattice mismatch. Importantly, strain effects are highly sensitive to particle size and shell thickness, making them a critical consideration in powder‐level design. Understanding and controlling lattice strain enables fine‐tuning of magnetic and biological responses without altering overall chemical composition [178, 184].

#### 4.7.4 In Situ TEM/STEM Insights Into Structural Evolution

Recent advances in in situ TEM and STEM techniques have enabled real‐time visualization of ferrite nanoparticle behavior under external stimuli, offering unprecedented insight into structure–property relationships. These studies reveal dynamic processes such as surface reconstruction, defect migration, aggregation, and shell degradation under magnetic fields, thermal cycling, or biological media exposure. In situ heating experiments demonstrate that morphological stability directly correlates with sustained magnetic heating efficiency, while liquid‐cell TEM has provided evidence of early‐stage protein adsorption and aggregation pathways. Such real‐time observations bridge the gap between static ex situ characterization and functional performance, highlighting failure mechanisms that are otherwise overlooked. Integrating in situ microscopy with powder engineering offers a powerful framework for designing structurally robust ferrite nanoparticles capable of maintaining performance under physiological conditions [178, 179].

## 5 Magnetic, Electrical, and Biological Characteristics of Ferrite Materials

Ferrite materials exhibit a unique combination of magnetic tunability, electrical resistivity, and biocompatibility, making them an exceptional class of multifunctional oxides for biomedical applications. Their performance in magnetic hyperthermia, targeted drug delivery, biosensing, and imaging largely depends on a delicate interplay between these properties. Understanding how magnetic and electrical characteristics influence biological interactions is essential for the rational design of safe and efficient ferrite‐based nanoplatforms.

### 5.1 Magnetic Characteristics

Magnetism forms the core functionality of ferrites in biomedical systems. The intrinsic magnetic behavior arises from the superexchange interaction between Fe^3+^ ions (and substituted cations) situated in tetrahedral (A) and octahedral (B) sites of the crystal lattice. The type and strength of magnetic ordering, such as ferrimagnetic, superparamagnetic, or paramagnetic, depend on particle size, cation distribution, and synthesis conditions.

#### 5.1.1 Types of Magnetic Behavior

- Ferrimagnetism: The magnetic nanoparticles like Mn_x_Fe_1‐x_Fe_2_O_4_, where antiparallel magnetic sublattices (A and B sites) yield a net magnetic moment. Such materials possess high coercivity and anisotropy, suitable for magnetic hyperthermia where large hysteresis losses generate heat efficiently [140].
- Superparamagnetism: Observed in nanosized ferrites (<20–30 nm) such as Fe_3_O_4_ or ZnFe_2_O_4_. These nanoparticles exhibit zero remanence and coercivity, preventing agglomeration and ensuring safe circulation in the bloodstream. Superparamagnetic nanoparticles respond rapidly to external magnetic fields, making them ideal for MRI contrast and magnetic drug targeting [185, 186].
- Paramagnetism: Common in doped ferrites (such as Gd^3+^‐ or Eu^3+^‐modified) that display enhanced spin relaxation beneficial for T_1_ imaging or theranostics [187].

#### 5.1.2 Key Magnetic Parameters and Biomedical Correlation

- Saturation magnetization (*M*_s_): Determines the magnetic responsiveness and heating capability. Higher *M*_s_ leads to stronger magnetic guidance and enhanced contrast in MRI. For example, CoFe_2_O_4_ nanoparticles exhibit *M*_s_ ≈ 80–90 emu/g, significantly higher than ZnFe_2_O_4_ (∼50 emu/g) [188, 189].
- Coercivity (*H*c): Represents magnetic hardness; moderate coercivity ensures efficient heat generation under alternating magnetic fields, critical for magnetic hyperthermia [190].
- Magnetic anisotropy (*K*): Influences relaxation mechanisms; higher anisotropy enhances energy dissipation but can hinder colloidal stability [191].
- Specific absorption rate (SAR): Quantifies heat produced in hyperthermia and depends on both intrinsic (composition, size) and extrinsic (field frequency, amplitude) parameters [192].

In biomedical systems, achieving high M_s_ with negligible remanence is the goal, ensuring magnetic efficiency without particle aggregation. Compositional tuning such as Co–Zn or Mn–Ni substitutions enables precise adjustment of magnetic parameters to meet specific clinical requirements.

#### 5.1.3 Magnetic Relaxation and Heat Dissipation Mechanisms

Ferrite nanoparticles dissipate magnetic energy primarily via Néel and Brownian relaxation mechanisms. Néel relaxation arises from magnetic moment flipping within a stationary particle. Brownian relaxation originates from the physical rotation of the nanoparticle in a viscous medium [193]. The relative contribution of each depends on particle size and surface coating. Small, well‐dispersed nanoparticles primarily undergo Néel relaxation, while larger or functionalized particles favor Brownian relaxation, which is a key factor in optimizing magnetic hyperthermia and MRI contrast efficiency [194].

In addition to intrinsic material properties, the efficiency of magnetic hyperthermia is critically governed by the characteristics of the applied alternating magnetic field and the associated hardware design. Clinically relevant performance is constrained by the product of magnetic field strength (*H*) and frequency (*f*), commonly referred to as the H·f safety limit, which is typically restricted to ∼5 × 10^9^ A/m s to avoid adverse biological effects. While high SAR values are often reported under elevated field amplitudes and frequencies, such conditions are not always compatible with clinical safety requirements. Recent work by Lei et al. [31] highlights the importance of resonant magnetic field generators and optimized coil design in enhancing heating efficiency within safe operational limits. Their study demonstrates that system‐level parameters, including frequency tuning and power efficiency, play a crucial role in determining the actual therapeutic performance. This perspective underscores that the translation of ferrite nanomaterials from laboratory‐scale studies to clinical applications requires an integrated approach that simultaneously optimizes nanoparticle properties and external field generation systems.

### 5.2 Electrical Characteristics

Although ferrites are primarily known for their magnetic properties, their electrical behavior significantly influences biocompatibility, charge transport, and interfacial interactions in physiological environments. Ferrites are semiconducting oxides, and their conductivity originates from electron hopping between Fe^2^^+^ and Fe^3^^+^ ions at the octahedral sites [129].

#### 5.2.1 Electrical Resistivity and Charge Transport

The electrical resistivity (*ρ*) of ferrites typically lies in the range of 10^2^–10^10^ Ω·cm, depending on synthesis conditions, dopants, and microstructure [195]. This high resistivity is advantageous in biomedical settings because it minimizes Joule heating and prevents undesired current flow when subjected to electromagnetic fields. The conduction in ferrites occurs via a small‐polaron hopping mechanism [159]. The hopping rate depends on cation distribution, crystallite size, and the presence of defects or oxygen vacancies. For instance, substitution of divalent cations like Ni^2+^ or Zn^2+^ modifies the Fe^2+^/Fe^3+^ ratio, thereby influencing charge mobility and dielectric loss [196, 197]. In biomedical applications, moderate conductivity ensures controlled energy dissipation, preventing overheating during hyperthermia treatments while maintaining responsiveness to alternating fields.

#### 5.2.2 Dielectric and Impedance Behavior

The dielectric constant (*ε*′) and loss tangent (tan δ) are crucial in determining interfacial polarization in biological systems. Ferrites with moderate dielectric constants can store and dissipate electromagnetic energy efficiently, which is useful for biosensors and microwave‐assisted therapies [28, 198]. Moreover, impedance spectroscopy studies reveal that grain boundaries and surfaces act as potential barriers, influencing ion exchange with biological fluids. The surface engineering (like silica or polymer coatings) reduces dielectric losses, improving colloidal stability and electrical neutrality, which is vital for biocompatibility. The interplay between electrical conductivity and dielectric response therefore dictates how ferrite nanoparticles interact with external stimuli and biological tissues. Balancing these parameters ensures both magnetic efficiency and biosafety.

### 5.3 Biological Characteristics

The biological behavior of ferrite materials encompasses biocompatibility, cytotoxicity, biodegradability, and bioactivity, which determine their clinical viability. Biological responses are influenced by multiple physicochemical parameters, including particle size, surface charge, coating chemistry, and magnetic properties.

#### 5.3.1 Biocompatibility and Cytotoxicity

Ferrite nanoparticles such as Fe_3_O_4_, ZnFe_2_O_4_, and MgFe_2_O_4_ exhibit excellent biocompatibility, largely attributed to their chemical stability and low ion leaching. The U.S. FDA‐approved Fe_3_O_4_ nanoparticles have established a clinical benchmark for magnetic contrast agents [199]. Cytotoxicity arises primarily from ROS (reactive oxygen species) generation or metal ion dissolution, which can damage cellular membranes [200]. Surface functionalization with polymers (PEG, dextran, chitosan) or silica significantly mitigates these effects by providing a steric barrier between the ferrite core and biological environment [201]. The acceptable dose range typically falls within 10–200 µg/mL for in vitro applications, depending on composition and coating type [202]. Long‐term cytotoxicity studies reveal that superparamagnetic ferrites are largely nontoxic and can be safely excreted via renal or hepatic pathways [203, 204]. While ferrite‐based nanomaterials generally demonstrate acceptable cytocompatibility, their long‐term in vivo performance is strongly influenced by post‐operative biological responses, including fibrosis, inflammation, and tissue adhesion. The formation of fibrotic capsules around implanted materials can significantly reduce therapeutic efficacy by limiting mass transport and isolating the material from surrounding tissue. Recent advances, such as the nano‐skin and surface lubrication strategies reported by Wang et al. [205], highlight the importance of dynamic and low‐friction interfaces in minimizing these adverse effects. Such approaches involve the creation of hydrated, lubricating surface layers that reduce protein adsorption, prevent nonspecific cell adhesion, and suppress inflammatory responses. Integrating these concepts into ferrite‐based systems through advanced surface functionalization can improve implant integration, reduce fibrotic encapsulation, and enhance long‐term biomedical performance. In addition to oncology‐related applications, ferrite‐based nanomaterials are also being investigated for the treatment of inflammatory diseases, such as rheumatoid arthritis. Recent studies, including Deng et al. [206], demonstrate that magnetic nanoparticles can be engineered to deliver anti‐inflammatory agents or modulate immune responses through controlled release and localized targeting. These systems enable site‐specific accumulation in inflamed tissues, reducing systemic side effects while improving therapeutic efficacy. Furthermore, the ability to combine magnetic targeting with drug delivery and imaging functionalities positions ferrite‐based nanomaterials as promising platforms for multimodal treatment of chronic inflammatory disorders.

#### 5.3.2 Cellular Uptake and Biodistribution

Cellular internalization of ferrite nanoparticles occurs mainly through endocytosis or phagocytosis, influenced by size, surface charge, and hydrophilicity. Nanoparticles <50 nm efficiently penetrate cell membranes [207]. Surface charges near neutrality (−10 mV ≤ *ζ* (zeta potential) ≤ +10 mV) reduce nonspecific interactions. PEGylation and ligand conjugation (e.g., folic acid, antibodies) enhance targeted uptake by specific cancer cells or tissues [208, 209, 210]. Once internalized, ferrites can localize within endosomes or lysosomes, where pH‐mediated dissolution may occur, releasing iron ions that participate in normal metabolic pathways [211].

#### 5.3.3 Antibacterial and Antioxidant Activity

Certain ferrites, notably CuFe_2_O_4_, ZnFe_2_O_4_, and CoFe_2_O_4_, exhibit intrinsic antibacterial and antifungal activity due to their ability to generate ROS (such as hydroxyl radicals, superoxide ions). These species disrupt bacterial membranes, inhibit biofilm formation, and promote wound healing [3]. In parallel, ferrites such as MnFe_2_O_4_ demonstrate antioxidant potential, scavenging free radicals and protecting tissues from oxidative stress, an essential property for anti‐inflammatory and regenerative therapies [212, 213].

#### 5.3.4 Hemocompatibility and Immune Response

For any intravenous or implantable system, hemocompatibility is a critical consideration. Ferrite nanoparticles should not induce hemolysis, platelet aggregation, or complement activation [214, 215]. Coatings such as albumin, dextran, or silica reduce hemolytic activity and prevent immune recognition [216]. Recent studies show that superparamagnetic ferrites with neutral surface charge exhibit minimal immune activation, highlighting their promise for safe systemic administration [1, 217].

### 5.4 Interrelationship Between Magnetic, Electrical, and Biological Behavior

A key challenge in ferrite design is achieving an optimal balance between magnetic performance and biological safety. Strong magnetism enhances targeting and hyperthermia efficiency but may induce aggregation or oxidative stress if not properly controlled. Similarly, electrical conductivity affects energy dissipation and heating uniformity in biological tissues [218]. Hence, structure–property–bioactivity correlation must guide the material design. (i) Magnetic behavior governs guidance, imaging, and heat generation [219, 220]. (ii) Electrical characteristics influence energy transfer, stability, and surface charge [221, 222, 223]. (iii) Biological interactions depend on surface chemistry, particle size, and ionic composition [224]. The compositional engineering can fine‐tune this triad, reducing toxicity while preserving magnetic efficiency.

Ferrite materials offer an unparalleled combination of magnetic tunability, electrical control, and biological adaptability, making them indispensable in modern biomedicine. Through precise structural and compositional optimization, it is possible to design ferrite nanoparticles that balance strong magnetic response with excellent biocompatibility. Future developments should focus on integrating real‐time in vivo monitoring with magnetic and electrical response mapping, employing computational and AI‐based design to predict property–function correlations, and establishing standardized biological evaluation protocols for toxicity, biodistribution, and long‐term effects. Overall, a comprehensive understanding of magnetic, electrical, and biological interdependencies provides the scientific foundation for the next generation of ferrite‐based nanomedicines capable of safe, efficient, and targeted biomedical interventions.

To strengthen cross‐study comparison and avoid treating SAR as an isolated material property, Table 4 summarizes a multilevel benchmarking framework for ferrite‐based magnetic hyperthermia. This framework separates intrinsic nanoparticle parameters from formulation effects, field‐generation constraints, biological microenvironmental factors, and reporting practices. Such separation is important because high SAR values measured in well‐dispersed colloids may not directly translate into intracellular or in vivo therapeutic efficacy. In particular, biological confinement can suppress Brownian relaxation, while field‐generator design, coil geometry, field homogeneity, and frequency–amplitude limits strongly influence the actual magnetic dose delivered to the target region.

**TABLE 4:** Multilevel benchmarking framework for ferrite‐based magnetic hyperthermia performance.

Benchmarking level and scope	Key parameter/Factor	Effect on hyperthermia performance	Critical comparison/Controversy	Recommended reporting criteria	Refs.
Material/core level: intrinsic ferrite properties before biological exposure	Composition, cation distribution, particle size, size distribution, crystallinity	Govern *M*_s_, *H*c, anisotropy, superparamagnetism, relaxation losses, and SAR/SLP	High‐anisotropy ferrites may enhance heating but can increase remanence, aggregation, or toxicity; the particle size that maximizes SAR may not be optimal for biodistribution or clearance	Report composition, phase purity, cation distribution, TEM/XRD size, size distribution, crystallinity, *M*_s_, *H*c, anisotropy, and SAR/SLP under fixed AMF conditions	[225, 226, 227, 228]
Material/core level: shape‐ and morphology‐dependent magnetic behavior	Morphology: spherical, cubic, rod‐like, flower‐like, clustered, or core–shell structures	Shape anisotropy and interparticle coupling can enhance hysteresis losses and heating efficiency	Nonspherical or clustered ferrites may show higher SAR, but may also show stronger dipolar interactions, reduced colloidal stability, or altered cellular uptake	Compare SAR as a function of morphology while keeping composition, concentration, coating, and AMF conditions constant	[226, 227]
Colloidal/formulation level: behavior after dispersion, coating, and surface functionalization	Surface coating, shell thickness, hydrodynamic diameter, zeta potential, colloidal stability	Controls dispersion, Brownian rotation, protein adsorption, circulation, immune recognition, and cellular uptake	Coatings improve biocompatibility and stability but may reduce heating by increasing hydrodynamic size or suppressing Brownian relaxation	Report coating chemistry, shell thickness, hydrodynamic size, zeta potential, PDI, serum stability, and SAR before/after coating	[229, 230]
Colloidal/formulation level: interparticle interactions in suspension or biological fluids	Aggregation state, cluster size, concentration, dipolar magnetic interactions	Aggregation can either enhance or suppress SAR depending on cluster geometry and magnetic coupling	SAR measured in dilute aqueous suspension may not represent ferrites in serum, culture medium, tumor tissue, or lysosomes	Report concentration‐dependent SAR, DLS in water/serum/media, aggregation before/after AMF exposure, and heating in viscous or immobilized media	[230, 231]
Magnetic‐field‐system level: external magnetic‐field exposure used to activate heating	Field amplitude, frequency, waveform, exposure duration	Determines energy input, relaxation losses, hysteresis losses, and apparent SAR/SLP	SAR values are not comparable unless AMF conditions are standardized	Report field amplitude, frequency, H × f, waveform, exposure time, sample volume, sample position, and temperature‐probe location	[219, 229, 230]
Magnetic‐field‐system level: hardware and field‐generation architecture	Coil geometry, field homogeneity, resonant circuit design, coil resistance, switching behavior, power‐transfer efficiency	Determines reproducibility, spatial field distribution, system heating, and practical therapeutic efficiency	A high‐SAR ferrite may appear ineffective if the field generator is inefficient, nonuniform, or thermally unstable	Report coil type, diameter, number of turns, field map, calibration method, field uniformity, power efficiency, and cooling conditions	[31, 230]
Magnetic‐field‐system level: translational safety window	Clinically acceptable frequency–amplitude range and tissue tolerance	Limits usable AMF conditions for in vivo and clinical applications	Ferrites optimized only under very high laboratory fields may not be clinically relevant	Benchmark SAR under biologically and clinically acceptable AMF conditions, not only under maximum laboratory field strength	[219, 228, 230]
Biological‐environment level: behavior after cell uptake or tissue localization	Colloidal versus intracellular heating; endosomal/lysosomal confinement	Intracellular confinement suppresses Brownian rotation and may shift heating toward Néel relaxation, hysteresis, magneto‐mechanical effects, or ROS‐mediated damage	The key controversy is whether intracellular hyperthermia is dominated by nanoscale heat, nonthermal magnetic effects, or secondary biological stress pathways	Compare heating in water, serum, viscous media, immobilized gels, cell pellets, and intracellular conditions; report uptake and intracellular localization	[231, 232]
Biological‐environment level: biological identity after protein and cell interaction	Protein corona, serum adsorption, immune recognition, endosomal/lysosomal localization	Alters biodistribution, aggregation, cellular uptake, clearance, and post‐uptake magnetic behavior	The biological identity of ferrites may differ from their synthetic identity; designed surface chemistry may not fully predict in vivo behavior	Report protein corona composition, serum stability, uptake pathway, intracellular localization, and post‐incubation magnetic/SAR behavior	[230]
Biological‐environment level: tumor microenvironment and therapeutic response	pH, viscosity, H_2_O_2_ level, hypoxia, tissue heterogeneity, heat diffusion	Influences relaxation dynamics, Fenton/Fenton‐like ROS generation, heat transfer, and therapeutic outcome	Ferrite therapy may arise from combined heat, ROS, drug release, and mechanical stress, making purely thermal interpretation difficult	Report temperature rise, ROS generation, cell viability, apoptosis/necrosis markers, AMF‐only controls, nanoparticle‐only controls, and combined‐therapy controls	[227]
Reporting/benchmarking level: cross‐study comparability of heating data	SAR/SLP calculation method, calorimetry protocol, magnetic mass normalization	Determines whether reported heating efficiencies are reliable and comparable	SAR varies with concentration, calorimetry method, heat‐loss correction, probe position, and initial‐slope fitting	Report SAR equation, magnetic mass basis, concentration, medium, heat‐capacity assumptions, insulation, fitting range, uncertainty, and replicate number	[226, 227, 228, 229, 230]

Ferrite‐mediated magnetic hyperthermia should be evaluated as an integrated material–formulation–field‐system–biological‐environment problem rather than through isolated SAR/SLP values. Intrinsic ferrite properties such as composition, particle size, crystallinity, morphology, saturation magnetization, and magnetic anisotropy define the theoretical heating potential. However, the experimentally observed and biologically relevant heating efficiency is also strongly affected by colloidal stability, surface coating, aggregation, field‐generator design, AMF frequency/amplitude, intracellular confinement, protein corona formation, and SAR reporting methodology.

## 6 Synthesis and Functionalization

### 6.1 Synthesis Approaches for Ferrite Nanoparticles

The production of ferrite nanoparticles for biomedical applications demands precise control over critical synthesis parameters, as these directly define structural and magnetic characteristics essential for therapeutic effectiveness. Parameters such as reaction temperature, pH, precursor concentration, and duration are meticulously adjusted to tailor nanoparticle attributes like shape, size distribution, and crystallinity. Uniformity in these properties is particularly important, as consistent particle morphology and size enable reproducible magnetic responses, a prerequisite for applications in imaging, targeted drug delivery, and hyperthermia therapy. Deviations or broad distributions in particle size or structure can undermine magnetic functionality and diminish biomedical performance.

Advancements in synthesis methodologies have enabled researchers to selectively engineer nanoparticle surface area, dispersibility, and porosity, optimizing them for cellular interactions, tissue integration, and enhanced imaging contrast. Choosing the right synthesis technique has become a strategic decision in translational research, balancing scalability, biocompatibility, and functional performance for diverse applications such as tumor imaging or energy‐based therapeutics.

**FIGURE 8:**
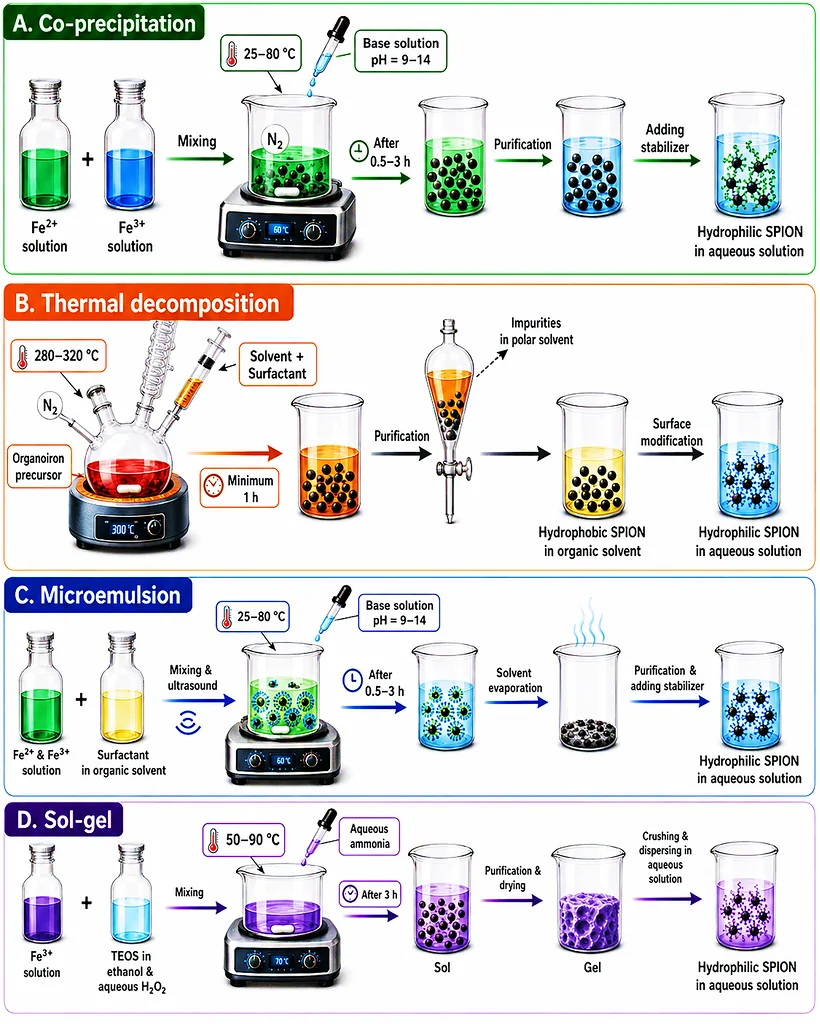
Schematics of commonly used synthesis strategies to prepare ferrite nanoparticles: (A) co‐precipitation, (B) thermal decomposition, (C) microemulsion, and (D) sol–gel routes, showing the key precursor mixing, nucleation, purification, stabilization, and aqueous dispersion steps.

Figure 8 is a schematic comparison of common synthesis strategies for producing superparamagnetic iron oxide nanoparticles. Figure 8 outlines four primary chemical routes: (A) Co‐precipitation, (B) thermal decomposition, (C) microemulsion, and (D) sol–gel. Each panel illustrates the distinct precursors, reaction conditions (e.g., solvent, temperature, and pH), and processing steps involved. While the initial synthesis conditions vary from aqueous to organic environments, all methods include necessary purification and, in some cases, surface modification steps to ultimately yield hydrophilic Iron Oxide Nanoparticles in an aqueous solution, which is crucial for biocompatibility and biomedical applications.

#### 6.1.1 Co‐Precipitation

The co‐precipitation route is extensively utilized for fabricating spinel ferrite nanoparticles due to its operational simplicity and scalability. Here, ferrous and ferric salts are co‐dissolved and subjected to alkaline precipitation, yielding nanoparticles as pH and temperature are tightly regulated [233, 234]. This method offers high yields and environmentally benign solvents but may require complex post‐processing to prevent agglomeration and improve colloidal stability [235]. Nevertheless, its reliability and ability to deliver uniform nanoparticles continue to support its widespread adoption in biomedical research.

#### 6.1.2 Thermal Decomposition

Thermal decomposition leverages thermolysis of organometallic precursors under inert atmospheres, often with surfactant capping for controlled particle growth. Despite demanding stringent conditions and higher energy input, this technique excels in producing monodispersed, highly crystalline, and structurally stable nanoparticles suitable for advanced imaging modalities [236, 237]. The use of toxic reagents limits biomedical translation, but ongoing research is refining greener alternatives.

#### 6.1.3 Microemulsion

Microemulsion synthesis utilizes nanoscale reactors provided by stabilized surfactant micelles within biphasic environments, facilitating spatially confined synthesis with tunable particle dimensions [238]. While this method achieves uniformity in size and shape, its low‐temperature reaction environments pose limitations in crystallinity, often necessitating additional thermal treatment [239]. Its scalability and the impact of residual surfactants on biological performance remain ongoing challenges.

#### 6.1.4 Sol–Gel

The sol–gel approach offers fine control over nanoparticle parameters via the hydrolysis and condensation of metal precursors under low temperatures. Tuning synthesis variables—temperature, pH, and solvent—enables the design of nanoparticles with customizable porosity and surface features, making them highly attractive for application as MRI contrast agents and targeted drug carriers [240]. The method's long reaction durations and the potential need for organic solvents are offset by the precision afforded over particle size and morphology.

#### 6.1.5 Hydrothermal

Hydrothermal synthesis involves reacting aqueous metal precursors under elevated temperature and pressure to generate highly crystalline nanoparticles with defined morphologies, including spheres, cubes, and hollow structures [241]. This method supports the production of nanoparticles with narrow size distributions and high purity, essential for reproducible in vivo behavior. Its flexibility allows for engineering nanostructures tailored to specific biomedical functions, such as drug delivery or imaging [242, 243].

#### 6.1.6 Biogenic Synthesis

Emerging biological synthesis strategies harness plant extracts, microbes, or other biological systems to yield ferrite nanoparticles in environmentally benign processes. While these routes offer advantages in biocompatibility and low toxicity, limitations include reduced yields and complex purification requirements [53, 244]. Nevertheless, bio‐inspired methods are gaining traction for next‐generation sustainable nanomedicine platforms.

### 6.2 Functionalization for Surface Modification and Biocompatibility Enhancement

Surface functionalization of ferrite nanoparticles (FNPs) is the central design tool that bridges their intrinsic magnetic behavior with the stringent requirements of in vivo safety, colloidal stability, and biological specificity. Pristine ferrite surfaces are often hydrophobic, prone to aggregation, and can catalyze reactive oxygen species (ROS) generation; therefore, rational surface engineering is indispensable to convert these inorganic cores into biocompatible, stealth, and targetable nanoplatforms for imaging, drug delivery, and theranostics.

**FIGURE 9:**
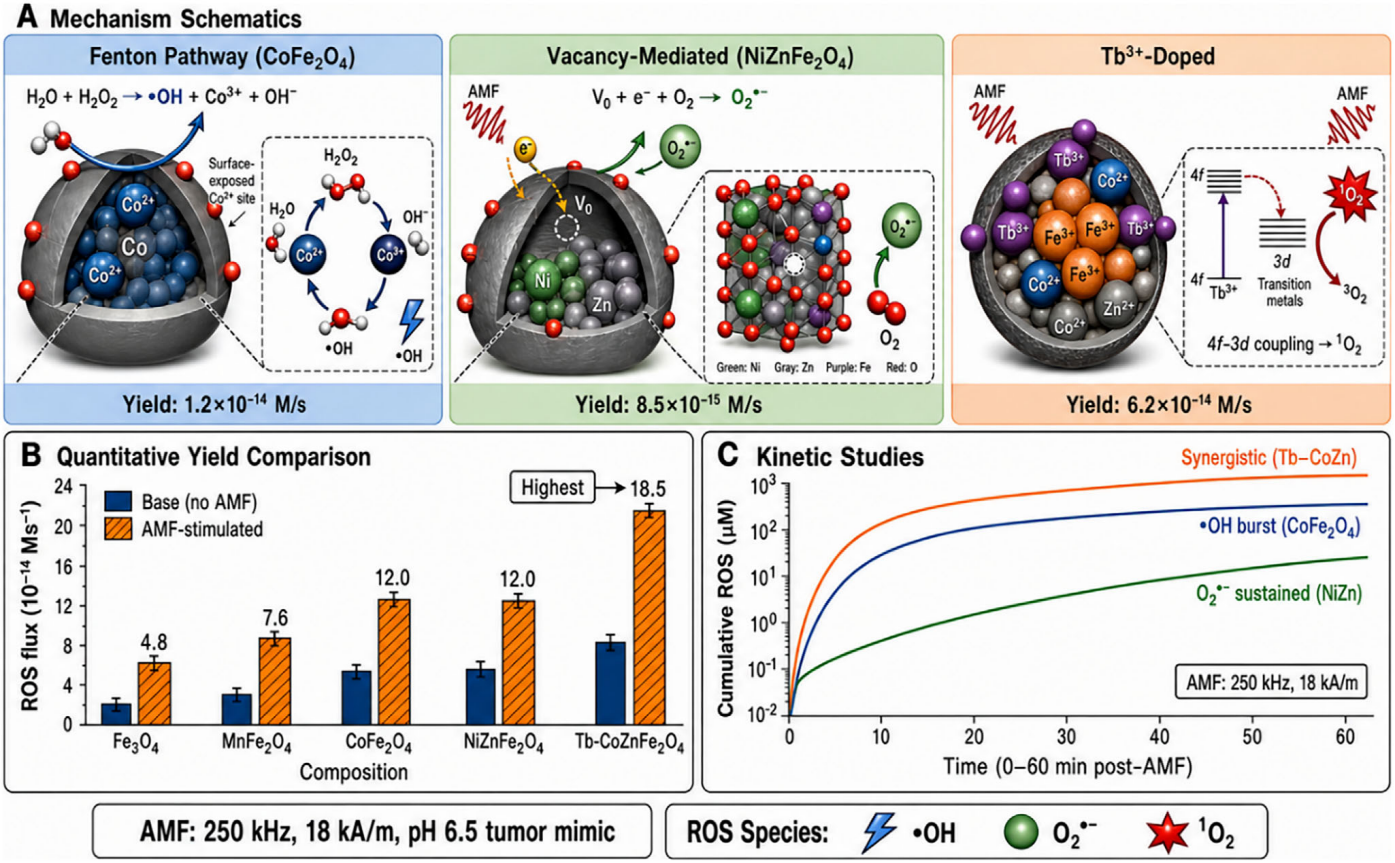
Quantitative ROS generation mechanisms and kinetics in spinel ferrite nanoparticles. (A) Schematic comparison of Fenton‐type, vacancy‐mediated, and Tb^3+^‐doped ROS‐generation pathways, highlighting the dominant active sites, reactive intermediates, and representative ROS yields. (B) ROS flux comparison for selected ferrite compositions under base and AMF‐stimulated conditions. (C) Cumulative ROS generation kinetics showing composition‐dependent •OH burst, O_2_^•−^‐sustained, and synergistic Tb–Co–Zn responses under AMF exposure. The bottom legend summarizes the AMF conditions and principal ROS species involved.

Figure 9 represents quantitative ROS generation mechanisms, yield comparisons, and kinetics in spinel ferrite nanoparticles under clinical alternating magnetic fields (AMF: 250 kHz, 18 kA/m). In Figure 9 panel A illustrates three dominant pathways: (1) Fenton chemistry in CoFe_2_O_4_ (Co^2+^+ H_2_O_2_ →—OH, 1 to 1.4 × 10^−14^ M/s or moles of—OH (hydroxyl radical) generated per liter per second), (2) oxygen vacancy‐mediated O_2_^−^‐ production in NiZnFe_2_O_4_ (7 to 9 × 10^−15^ M/s), and (3) rare‐earth enhanced ^1^O_2_ generation via 4f‐3d electronic coupling in Tb^3+^‐doped CoZn ferrites (6 to 7 × 10^−14^ M/s), the highest yield observed [245]. Panel B compares steady‐state ROS flux across compositions, revealing a 3‐fold AMF stimulation for defect‐engineered systems (CoFe_2_O_4_: 12.0 → 30 to 38 × 10^−14^ M/s; Tb‐CoZn: 15 to 20 ×10^−14^ M/s peak performance). This synergy arises from magnetic hyperthermia (42–47°C) accelerating Fenton kinetics while lattice vacancies serve as electron traps, amplifying ROS burst efficiency in tumor microenvironments (pH 6.5, elevated H_2_O_2_) [246]. Panel C demonstrates distinctive kinetic profiles critical for therapy design: CoFe_2_O_4_ exhibits a rapid—OH burst (peak 5 min, plateau 30 min) ideal for acute ablation, while NiZn systems sustain O_2_^−^—release linearly over 60 min for chronic oxidative stress. Tb^3+^‐doped ferrites display a 4x steeper initial slope, combining burst + sustained phases optimal for synergy with pH‐responsive drug release (DOX IC_50_ reduction >5x under combined ROS+heat) [247]. These quantitative relationships are compiled to establish structure–ROS efficacy correlations guiding next‐generation defect‐engineered ferrites beyond empirical optimization. Tb^3+^‐doping emerges as a breakthrough for clinical translation, achieving therapeutic ROS thresholds at safe field amplitudes [248].

**FIGURE 10:**
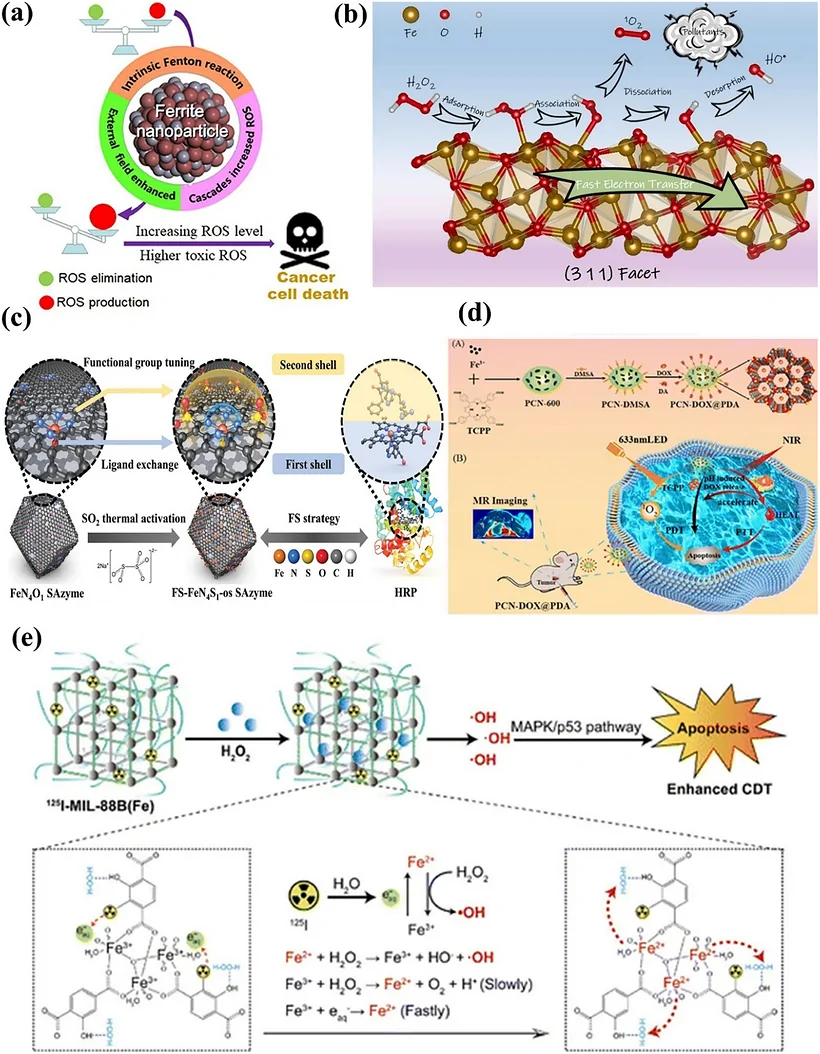
(a) Schematic illustration of the ferrite nanoparticles‐based ROS‐mediated cancer therapy, (b) The mechanism of POD‐like activity of α‐Fe_2_O_3_ with (311) facet, (c) Schematic view of the synthesis of the FS‐FeN4S1‐os SAzyme, (d) Schematic view of tumor therapy using PCN‐DOX@PDA, (e) 125I enhanced Fenton reaction. (a) Reproduced under the terms of the CC‐BY Creative Commons Attribution 4.0 International license. (http://creativecommons.org/licenses/by/4.0/) [246]. Copyright 2021, The Authors, published by Frontiers. (b) Reproduced with permission [252]. Copyright 2024, Elsevier. (c) Reproduced with permission [253]. Copyright 2024, Wiley‐VCH. (d) Reproduced with permission [254]. Copyright 2023, American Chemical Society. (e) Reproduced with permission [255]. Copyright 2024, Wiley‐VCH.

Extensive studies have explored the use of ferrite‐based nanomaterials as nanozymes capable of mediating reactive oxygen species (ROS) generation for cancer therapy. The overall concept, illustrated schematically in Figure 10a, highlights how ferrite nanoparticles can catalytically promote ROS formation to induce tumor cell death. Broadly, their ROS‐mediating functions can be categorized into three major mechanisms.

- Intrinsic Fenton catalytic activity

Ferrite nanoparticles exhibit inherent Fenton or Fenton‐like catalytic properties, which represent the primary pathway employed in ROS‐driven cancer treatment. After preferentially accumulating within tumor tissues facilitated by the enhanced permeability and retention (EPR) effect and, in some cases, magnetic targeting these nanoparticles release Fe^2+^/Fe^3+^ ions in the mildly acidic tumor microenvironment. These ions subsequently react with endogenous H_2_O_2_ to generate highly cytotoxic hydroxyl radicals (•OH), thereby triggering oxidative damage to cancer cells [246].

- External‐field‐assisted enhancement

A second strategy involves boosting the intrinsic Fenton reaction using external stimuli, such as magnetic fields, ultrasound, or light irradiation. Such external activation significantly improves catalytic efficiency, enabling higher ROS yields even at relatively lower nanoparticle doses an important consideration for maximizing therapeutic efficacy while minimizing systemic toxicity [249, 250].

- Cascade catalytic ROS amplification

The third mechanism encompasses cascade reactions designed to further enhance ROS generation. By integrating multistep catalytic processes, ferrite nanozymes can alleviate tumor hypoxia one of the major barriers to ROS‐based therapy and simultaneously elevate overall ROS production, resulting in improved tumor suppression [251].

The monometallic iron oxides such as hematite and magnetite are widely studied nanozymes for tumor therapy owing to their simple structures and tunable physicochemical properties. Variations in size, morphology, and exposed facets strongly influence their Fenton and Fenton‐like catalytic activity. For example, α‐Fe_2_O_3_ nanosheets with dominant (311) facets (Figure 10b) exhibit enhanced POD‐like activity due to stronger H_2_O_2_ adsorption and lower activation barriers, enabling efficient •OH generation and ferroptosis initiation.

Single‐atom engineering further advances catalytic performance. Fe‐N_4_ SAzymes modified with reduced/oxidized sulfur ligands (Figure 10c) show optimized second‐shell coordination, reduced reaction energy barriers, and significantly improved tumor‐cell cytotoxicity. Heteroatom incorporation, such as Mn‐doping in Fe‐based SAzymes, shifts the D‐band center and strengthens H_2_O_2_ affinity, thereby increasing POD‐like activity.

Metal–organic frameworks (MOFs) offer additional advantages through porous structure, tunable chemistry, and drug‐delivery capability. Fe‐MOFs loaded with DOX and coated with PDA (Figure 10d) exhibit pH/NIR‐responsive drug release and ROS generation, enhancing multimodal tumor therapy. MOFs can also be engineered for synergistic catalytic effects; for instance, ^125^I‐doped MIL‐88B(Fe) (Figure 10e) produces hydrated electrons that continuously regenerate Fe^2+^ from Fe^3+^, markedly amplifying CDT efficacy.

Surface chemistry of FNPs controls nearly all “biological” parameters: protein corona composition, opsonization, circulation time, cellular uptake, and intracellular fate. Even when core composition and size are optimal, unmodified ferrites interact nonspecifically with plasma proteins, leading to rapid clearance by the mononuclear phagocyte system and off‑target accumulation in liver and spleen. Functional coatings minimize direct contact between the inorganic surface and biological milieu, attenuate metal ion leaching, and tune surface charge to reduce nonspecific adsorption and cytotoxicity. A further motivation is the need for multifunctionality: the same nanoparticle must be magnetically responsive, water‑dispersible, non‑immunogenic, and often capable of loading therapeutic cargos or molecular probes. This requirement has driven the evolution from simple single‑layer stabilizers to hierarchical core–shell and multilayer architectures that integrate inorganic, polymeric, and bio‑ligand components in a modular fashion.

**FIGURE 11:**
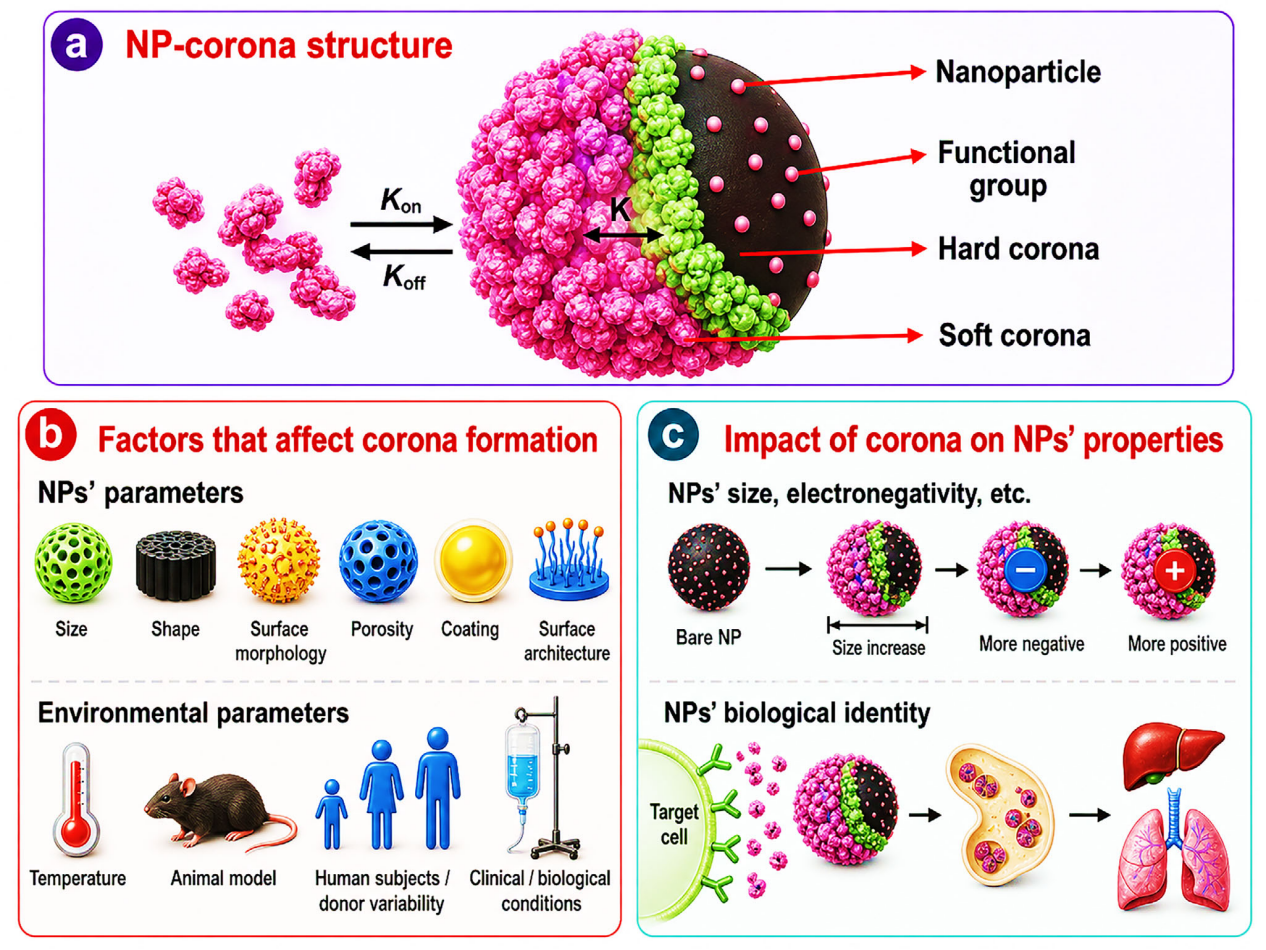
Protein corona formation and its impact on nanoparticle bioidentity. (a) NP–corona interface showing biomolecular adsorption/desorption, surface functional groups, and hard/soft corona layers. (b) Physicochemical and biological factors regulating corona formation. (c) Corona‐induced changes in nanoparticle size, surface charge, cellular interactions, uptake, and organ‐level biodistribution.

Engineered nanoparticles rapidly acquire a protein corona (PC) when exposed to biological fluids due to their high surface energy. As shown in Figure 11a, corona formation is a dynamic Vroman‐driven process in which abundant proteins bind first and are later replaced by higher‐affinity proteins [257]. This leads to two layers: a hard corona (HC) of tightly bound, long‐residence proteins and a soft corona (SC) of loosely bound, rapidly exchanging proteins [258]. The final composition of the PC depends strongly on nanoparticle physicochemical properties, including size, shape, charge, and most critically surface modification (Figure 11b). Surface functionalization can reduce nonspecific protein adsorption, tune protein–NP affinity, improve colloidal stability, and modulate how HC and SC evolve in different biological environments. External factors such as medium type, pH, temperature, and biological fluid source further influence corona structure and dynamics [256]. As illustrated in Figure 11c, the PC ultimately defines the biological identity of nanoparticles, affecting their toxicity, biodistribution, biodegradation, immune interactions, and targeting efficiency [259, 260, 261]. Therefore, surface modification strategies are essential for controlling corona formation and optimizing nanomaterial performance. Understanding PC behavior requires reliable methods for NP–PC isolation, analysis, and characterization, which continue to evolve alongside advances in nanoparticle design.

In physiological media, FNPs experience high ionic strength, competing biomolecules, and shear forces that promote aggregation and dynamic protein corona formation. Poorly stabilized particles agglomerate, lose magnetic addressability at the single‑particle level, and exhibit altered biodistribution and toxicity profiles compared with well‑dispersed systems [262]. Appropriate surface functionalization introduces steric and/or electrostatic repulsion that maintains colloidal stability in complex fluids such as serum, plasma, and cell culture media. The concept of the protein corona is now recognized as a major determinant of the “biological identity” of FNPs. Surface coatings modulate which proteins preferentially adsorb, thereby influencing cellular recognition pathways, immune activation, and organ targeting. Hydrophilic neutral or zwitterionic shells (e.g., polyethylene glycol, certain polysaccharides) tend to suppress opsonin adsorption and favor a “stealth” corona, whereas positively charged or hydrophobic coatings recruit complement and immunoglobulins that accelerate clearance [263, 264]. Advanced surface design thus aims not only at preventing aggregation but at engineering a favorable corona that prolongs circulation while preserving targeting capabilities.

Silica is a widely used inorganic coating for ferrite nanoparticles because of its chemical stability, tunable porosity, and ease of functionalization. A uniform silica shell minimizes magnetic dipolar interactions, prevents aggregation, and enhances dispersibility and biocompatibility by shielding reactive metal sites [265]. Methods such as Stöber sol–gel, microemulsion, and silicate deposition enable precise control of shell thickness. The silica surface can be further modified with functional organosilanes (e.g., amino, carboxyl, and PEG), allowing attachment of drugs, fluorophores, or targeting ligands [266]. This core–shell architecture provides magnetic functionality combined with improved stability and biointerface control. Similar coating strategies using gold, titania, or carbon offer additional optical, catalytic, or electrochemical advantages.

**FIGURE 12:**
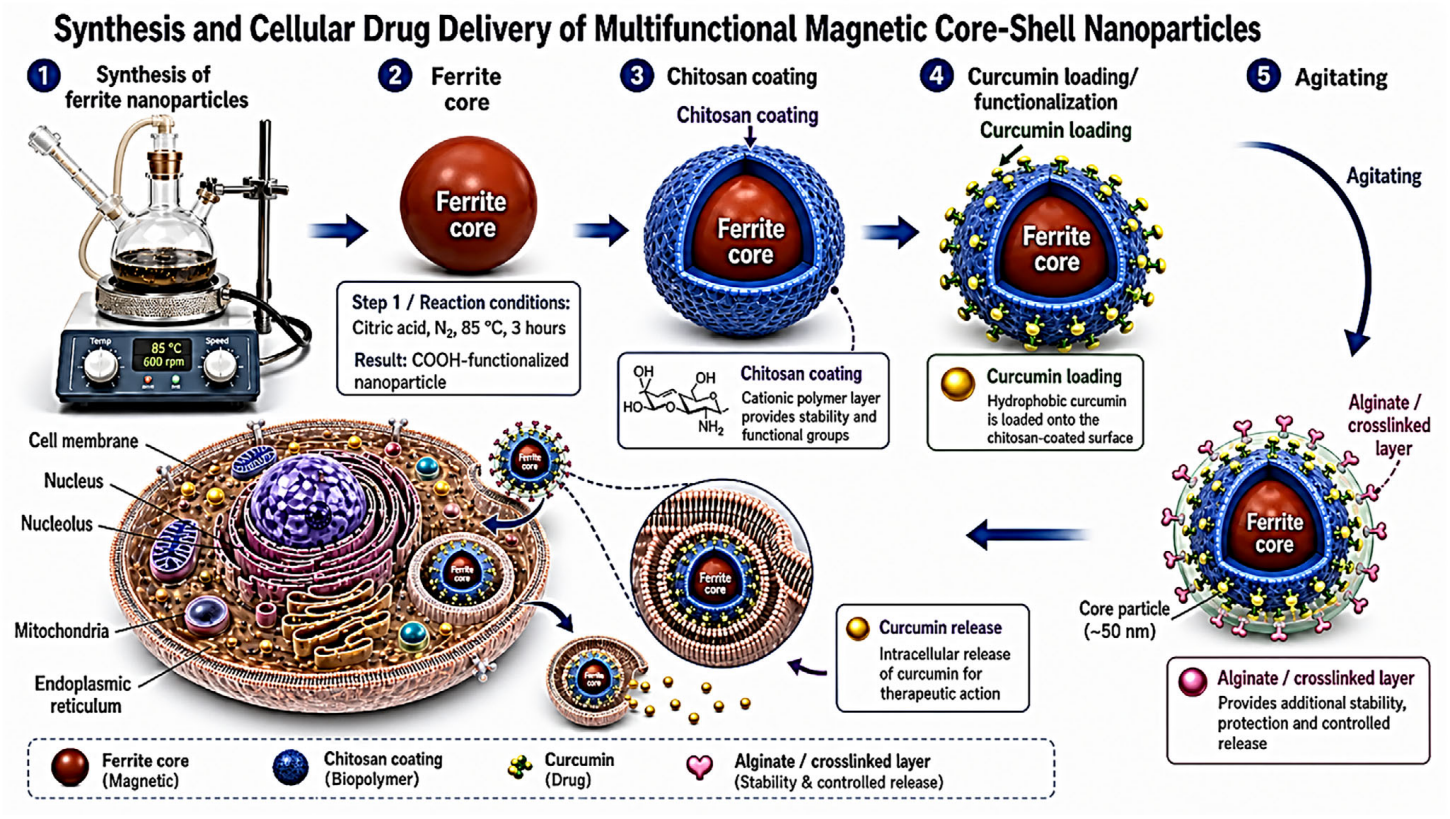
Schematic representation of the synthesis and cellular delivery of multifunctional ferrite‐based magnetic core–shell nanoparticles. The ferrite core is functionalized with citric acid, coated with chitosan, loaded with curcumin, and further stabilized with an alginate/crosslinked layer to enable cellular uptake and controlled intracellular drug release.

Ferrite nanoparticle surfaces are commonly engineered with polymers, polysaccharides, and small ligands to enhance stability, dispersibility, and biological performance. Hydrophilic polymers such as PEG, PVA, PAA, dextran, and biodegradable PLGA or chitosan reduce aggregation, suppress protein adsorption, and enable high drug‐loading with controlled release. Natural macromolecules like dextran, alginate, and hyaluronic acid improve biocompatibility and can provide active targeting, as shown in Figure 12 [267]. Overall, surface functionalization governs ion leakage, ROS suppression, protein corona formation, and in vivo fate, making it critical for safe and effective biomedical applications of ferrite nanoparticles.

## 7 Toxicity and Biocompatibility of Ferrite Nanomaterials

The integration of ferrite nanomaterials in biomedical applications mandates rigorous evaluation of their toxicity and biocompatibility to ensure safety and efficacy. Despite the promising magnetic properties and functional versatility of ferrite nanoparticles (FNPs), their interactions with biological systems can lead to adverse effects if not properly engineered. Consequently, systematic in vitro and in vivo investigations have been pivotal in elucidating cellular responses, biodistribution, and systemic toxicity profiles [268, 269].

Ensuring the biocompatibility and safety of ferrite nanomaterials is essential before they can be translated into clinical or therapeutic applications. While ferrites such as Fe_3_O_4_, ZnFe_2_O_4_, and MnFe_2_O_4_ are generally recognized as biocompatible due to their chemical stability and acceptable iron metabolism pathways, their toxicity fundamentally depends on composition, particle size, surface charge, dose, aggregation behavior, and surface functionalization [270, 271]. Additionally, dopants such as Co, Ni, and rare‐earth elements can significantly alter biological responses, demanding careful toxicity assessment. Toxicity primarily arises from oxidative stress, inflammatory responses, membrane destabilization, and metal ion leaching. Surface coatings, especially polymers and biomolecules, play critical roles in reducing adverse reactions by improving colloidal stability, reducing protein corona formation, and minimizing nonspecific cellular interactions [272, 273]. Hence, toxicity evaluation is a multidimensional process that involves in vitro, in vivo, and mechanistic studies.

### 7.1 In Vitro and in Vivo Toxicity Evaluations

#### 7.1.1 In Vitro Toxicity Assessments

**FIGURE 13:**
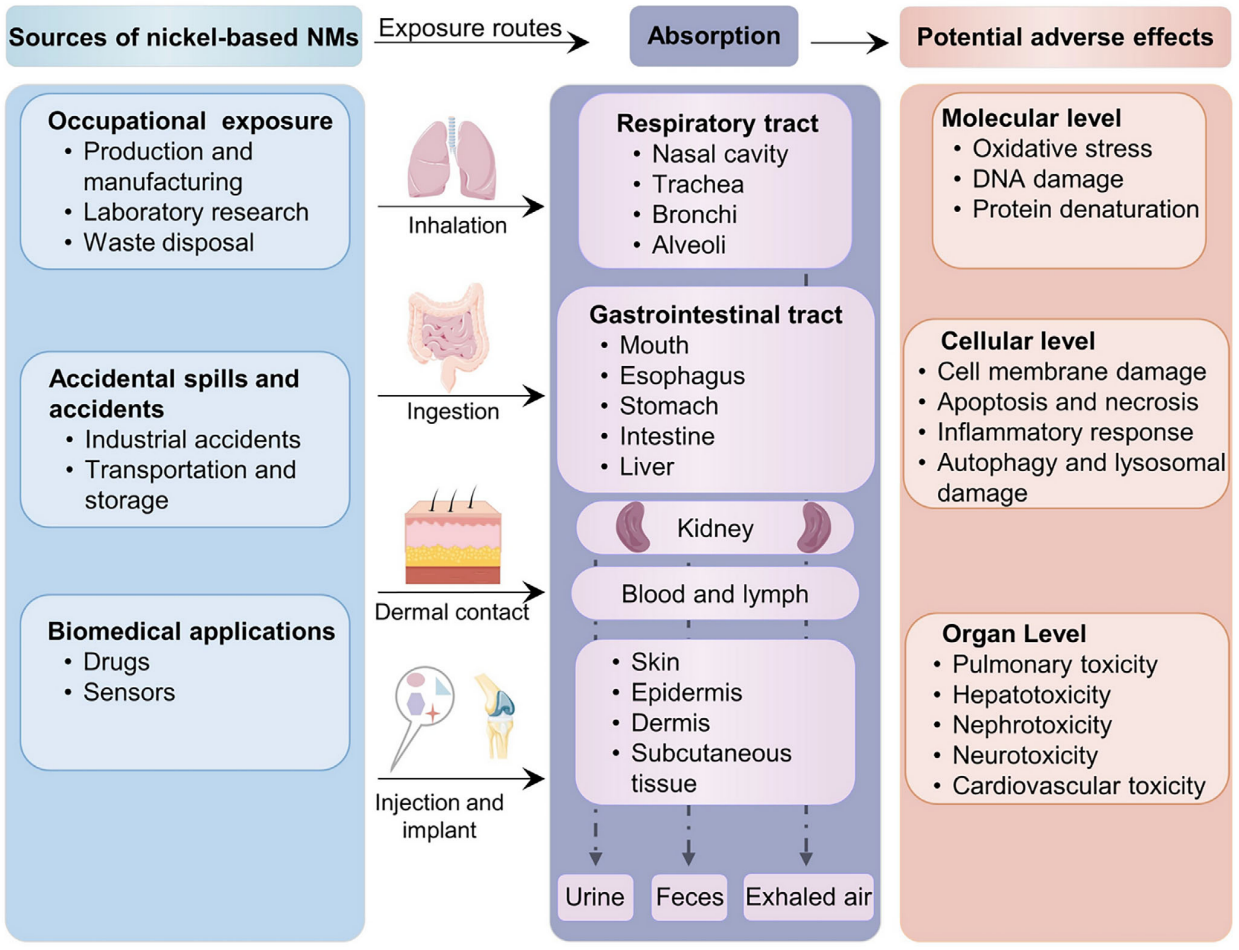
Overview of the exposure pathways and toxicological profile of nickel‐based nanomaterials. Reproduced under the terms of the CC‐BY Creative Commons Attribution 4.0 International license. (http://creativecommons.org/licenses/by/4.0/) [274]. Copyright 2025, Springer‐Nature. The Authors.

The Figure 13 illustrates the progression from sources of exposure (occupational, accidental, and biomedical applications) to the biological entry routes (inhalation, ingestion, dermal contact, and injection). It details the absorption and biodistribution through the respiratory and gastrointestinal tracts into the bloodstream and lymph systems. Finally, it categorizes the potential adverse effects at three distinct biological levels: molecular (e.g., oxidative stress, DNA damage), cellular (e.g., apoptosis, membrane damage), and organ‐level systemic toxicity (affecting the lungs, liver, kidneys, and nervous system) [274].

Cell culture‐based assays provide a foundational platform to scrutinize the cytotoxicity, genotoxicity, oxidative stress induction, and inflammatory potential of ferrite nanomaterials. Standard methodologies such as MTT/MTS assays, lactate dehydrogenase (LDH) release, reactive oxygen species (ROS) quantification, and apoptosis assays have been employed to evaluate cell viability and integrity upon exposure to FNPs. Functionalization strategies that improve surface biocompatibility, such as coating with biopolymers like gelatin or polyethylene glycol (PEG), consistently reduce cytotoxic responses in various cell lines [275, 276]. Notably, the physicochemical characteristics of FNPs, including size, surface charge, crystallinity, and aggregation state, significantly influence cellular uptake and toxicity. Smaller nanoparticles with optimized surface coatings exhibit reduced nonspecific uptake and lower induction of oxidative stress, thereby mitigating cytotoxicity. Furthermore, various studies report that surface‐functionalized ferrites can even selectively induce apoptosis in cancer cells while sparing healthy cells, illustrating their therapeutic potential alongside acceptable biocompatibility [275].

Hemocompatibility assessment forms another essential component of in vitro studies because many biomedical applications, especially imaging, drug delivery, and hyperthermia, involve systemic administration [277]. Hemocompatibility tests evaluate the interaction of nanoparticles with erythrocytes, platelets, and coagulation pathways [278]. Ferrites with neutral or slightly negative surface charges generally exhibit excellent blood compatibility, whereas highly positive surfaces can induce hemolysis or platelet activation [279, 280]. Surface functionalization plays a decisive role here as well, as coated ferrites tend to interact minimally with blood components, thereby reducing the risk of clot formation or undesired immune activation [281, 282].

#### 7.1.2 In Vivo Toxicity and Biodistribution

Animal model studies provide vital insights into the systemic effects, pharmacokinetics, and organ‐specific accumulation of ferrite nanoparticles. Typical administration routes include intravenous, intraperitoneal, or oral, depending on the intended application. Comprehensive hematological analyses, histopathological examinations, biochemical assays for liver and kidney function, and immunological profiling collectively inform on the biocompatibility of these nanomaterials in vivo.

**FIGURE 14:**
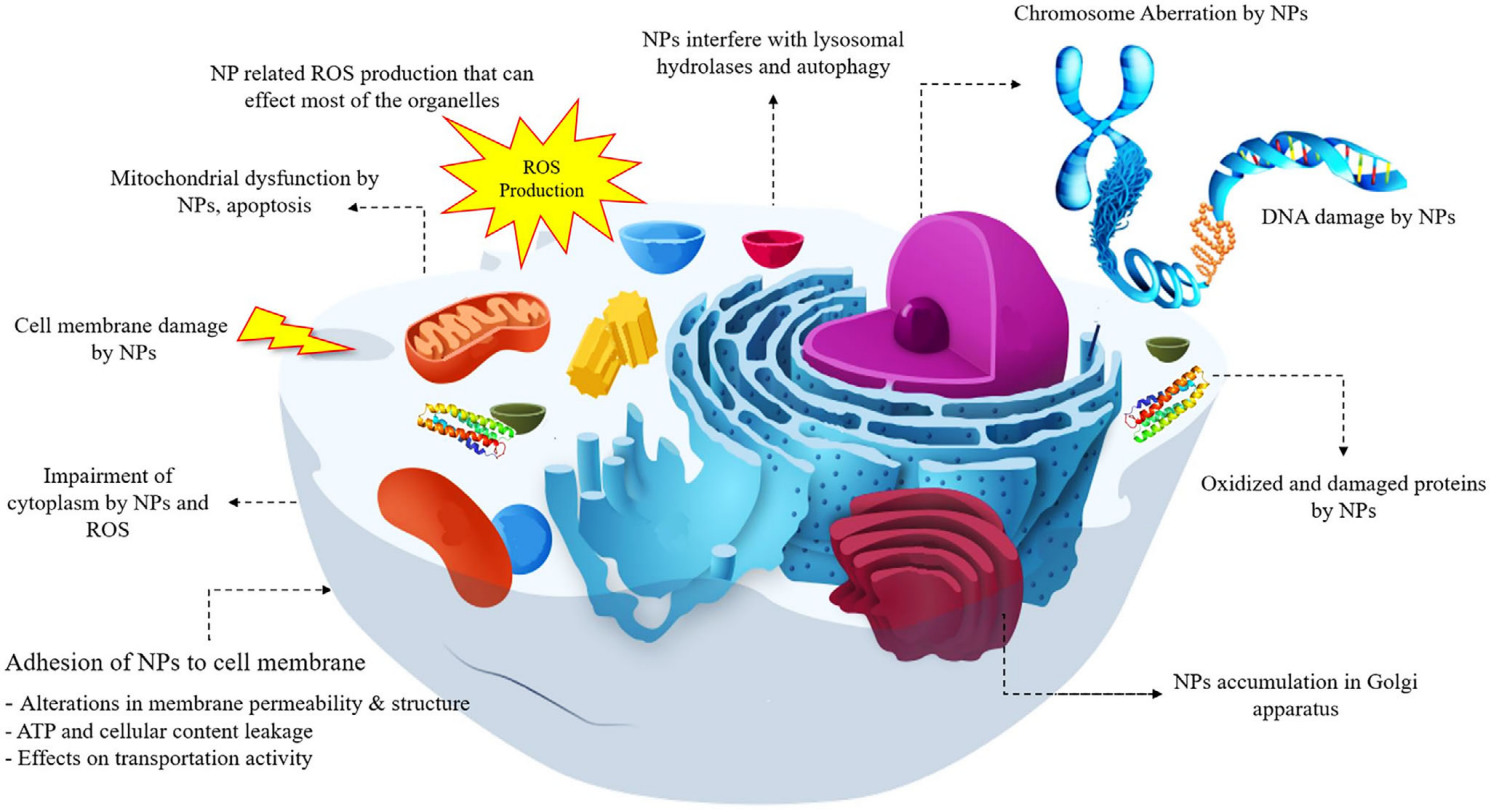
Representation of common mechanisms underlying nanoparticle‐induced toxicity. Reproduced under the terms of the CC‐BY Creative Commons Attribution 4.0 International license. (http://creativecommons.org/licenses/by/4.0/) [283]. Copyright 2020, The Authors, published by Frontiers.

Nanoparticles interact extensively with cellular components such as DNA, proteins, and mitochondria, triggering multiple toxicity mechanisms as shown in Figure 14. These interactions can induce the formation of reactive oxygen species (ROS), which disrupt various cellular functions [283]. Consequently, NPs may cause DNA damage, activate lysosomal hydrolases, impair mitochondrial function, lead to apoptosis, damage cell membranes, alter cytoplasmic integrity and ATP levels, increase cell membrane permeability, accumulate in the Golgi apparatus, and affect protein expression and function. These multifaceted effects reflect the complex cellular impact of NPs.

Clinical relevance hinges on achieving an optimal balance between magnetic functionality and minimal toxicity. Surface functionalization has been shown to dramatically influence in vivo behavior by reducing rapid clearance by the reticuloendothelial system (RES) and improving circulation half‐life. Notably, polymer coatings such as PEG and gelatin reduce opsonization, preventing immune recognition, thus lowering inflammatory responses and acute toxicity [275, 284]. Long‐term studies underscore the importance of biodegradability and clearance pathways of ferrite nanoparticles. Inert coatings facilitate safe elimination via renal and hepatobiliary routes, mitigating risks of chronic accumulation and toxicity. Nonetheless, uncoated or inadequately protected nanoparticles may provoke oxidative damage, inflammatory cascades, and organ dysfunction, particularly in the liver, spleen, and lungs [275].

### 7.2 Surface Engineering for Safety and Biomedical Compatibility

Surface engineering is pivotal for translating ferrite nanoparticles into safe, clinically relevant nanomedicines because it governs how these materials interact with biological barriers, fluids, and cells. Pristine ferrite surfaces often promote aggregation, protein adsorption, ion release, and reactive oxygen species (ROS) generation, all of which can trigger cytotoxicity and rapid clearance [285, 286]. Rational surface modification using polymers, inorganic shells, and bioinspired coatings aims to transform these reactive interfaces into stable, biocompatible, and functionally tunable platforms without compromising the magnetic performance required for imaging, hyperthermia, or magnetically guided drug delivery [287, 288].

**FIGURE 15:**
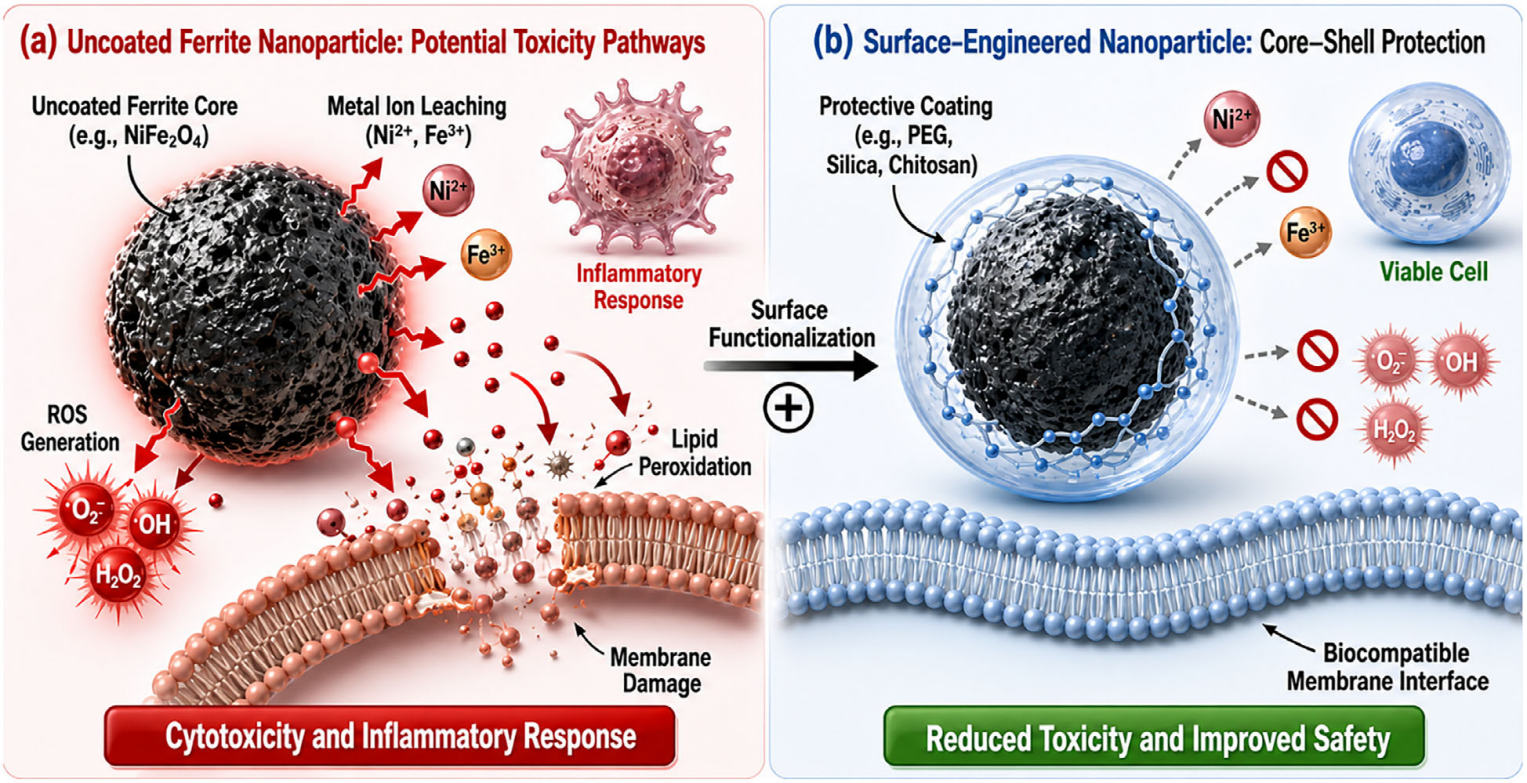
Surface engineering strategies for improving the safety and biocompatibility of ferrite nanoparticles. (a) Uncoated ferrite nanoparticles may induce metal‐ion leaching, ROS generation, lipid peroxidation, membrane damage, and inflammatory responses. (b) Surface‐functionalized core–shell nanoparticles suppress direct core–biointerface interactions, reduce ion leaching and oxidative stress, and promote a more biocompatible membrane interface.

As illustrated in Figure 15, the biocompatibility of ferrite nanoparticles is fundamentally governed by their surface chemistry. Uncoated nanoparticles (Figure 15a) are prone to degradation in physiological environments, leading to the leaching of toxic cations (such as nickel and iron) and the catalytic generation of reactive oxygen species (ROS). These factors synergistically attack the cell membrane, causing lipid peroxidation and inflammation. Conversely, the implementation of a core‐shell architecture (Figure 15b) serves as a robust barrier. By encapsulating the magnetic core with inert polymers (e.g., PEG, chitosan) or inorganic shells (Silica), the release of toxic ions is blocked, and the production of ROS is minimized, thereby transforming a potentially cytotoxic material into a safe, biocompatible platform for biomedical use.

Hydrophilic polymers such as polyethylene glycol (PEG), poly(vinyl alcohol), and amphiphilic copolymers create steric stabilization layers that improve aqueous dispersibility and reduce opsonization, thereby prolonging blood circulation and reducing uptake by the reticuloendothelial system. Natural polymers including gelatin, chitosan, dextran, and alginate further enhance biocompatibility and offer functional groups for drug loading or ligand attachment; gelatin‐ and chitosan‐coated ferrites, for example, show reduced hemolysis and inflammation while enabling controlled release of therapeutic cargo [289, 290, 291]. Inorganic shells, particularly silica and calcium phosphate, provide robust barriers that limit metal‐ion leaching and surface‐catalyzed ROS formation, and their chemically versatile surfaces facilitate subsequent conjugation of targeting ligands or imaging agents. Fine‐tuning of zeta potential and hydrophilicity through appropriate choice and combination of coating chemistries helps balance colloidal stability with cellular uptake, typically favoring slightly negative or near‐neutral surfaces to minimize nonspecific toxicity [276, 292, 293, 294].

These surface‐engineering strategies directly mitigate key nanotoxicology pathways, including oxidative stress, membrane disruption, lysosomal damage, and uncontrolled biodistribution, while enabling active targeting and stimuli‐responsive behavior through conjugation of antibodies, peptides, or pH/redox‐sensitive linkers [295]. Overall, the nano–biointerface defined by the coating now functions as the primary design space for ferrite nanomaterials, allowing safety, pharmacokinetics, and therapeutic performance to be engineered in an integrated manner for advanced biomedical applications [180, 269].

### 7.3 Comprehensive Toxicity and Biocompatibility Assessment of Spinel Ferrite Nanoparticles

Despite their excellent magnetic and catalytic properties, the successful clinical translation of spinel ferrite nanoparticles hinges on establishing robust toxicity profiles and biodistribution understanding across acute, chronic, and organ‐specific exposure scenarios. This comprehensive assessment integrates quantitative IC50 metrics, systemic clearance pathways, immunogenic responses, and degradation kinetics to define maximum tolerated doses and quality control standards essential for GMP manufacturing and Phase I trial design

#### 7.3.1 Quantitative Toxicity Profiles of Ferrite Nanoparticles

While ferrite nanoparticles are generally regarded as biocompatible, their toxicity strongly depends on composition, size, surface chemistry, dose, and exposure time. Quantitative assessment using IC_50_ values and time‐dependent viability measurements provides a more rigorous basis for safety evaluation. Table 5 summarizes representative toxicity thresholds reported for common ferrite systems across different cell types. Notably, cancer cells often exhibit higher sensitivity due to enhanced nanoparticle uptake and oxidative stress susceptibility, whereas normal cells typically tolerate higher concentrations. Importantly, toxicity frequently evolves with exposure duration, underscoring the necessity of time‐resolved evaluation rather than single‐time‐point assays.

**TABLE 5:** Representative quantitative toxicity metrics of ferrite nanoparticles.

Ferrite composition	Cell type	IC_50_ (µg/mL)	Exposure time	Viability threshold
Fe_3_O_4_	Fibroblasts	>250	24–72 h	>90% @ ≤200 µg/mL [296]
NiZn ferrite	Cancer cells	60–150	24–72 h	Dose‐dependent [275, 297]
MnFe_2_O_4_	Endothelial	>150	24–72 h	>85% [177, 181]
CoFe_2_O_4_	Cancer cells	40–100	24–72 h	ROS‐mediated [296, 297, 298]
Core–shell ferrite	Hepatocytes	>300	72 h	>90% [299]

**FIGURE 16:**
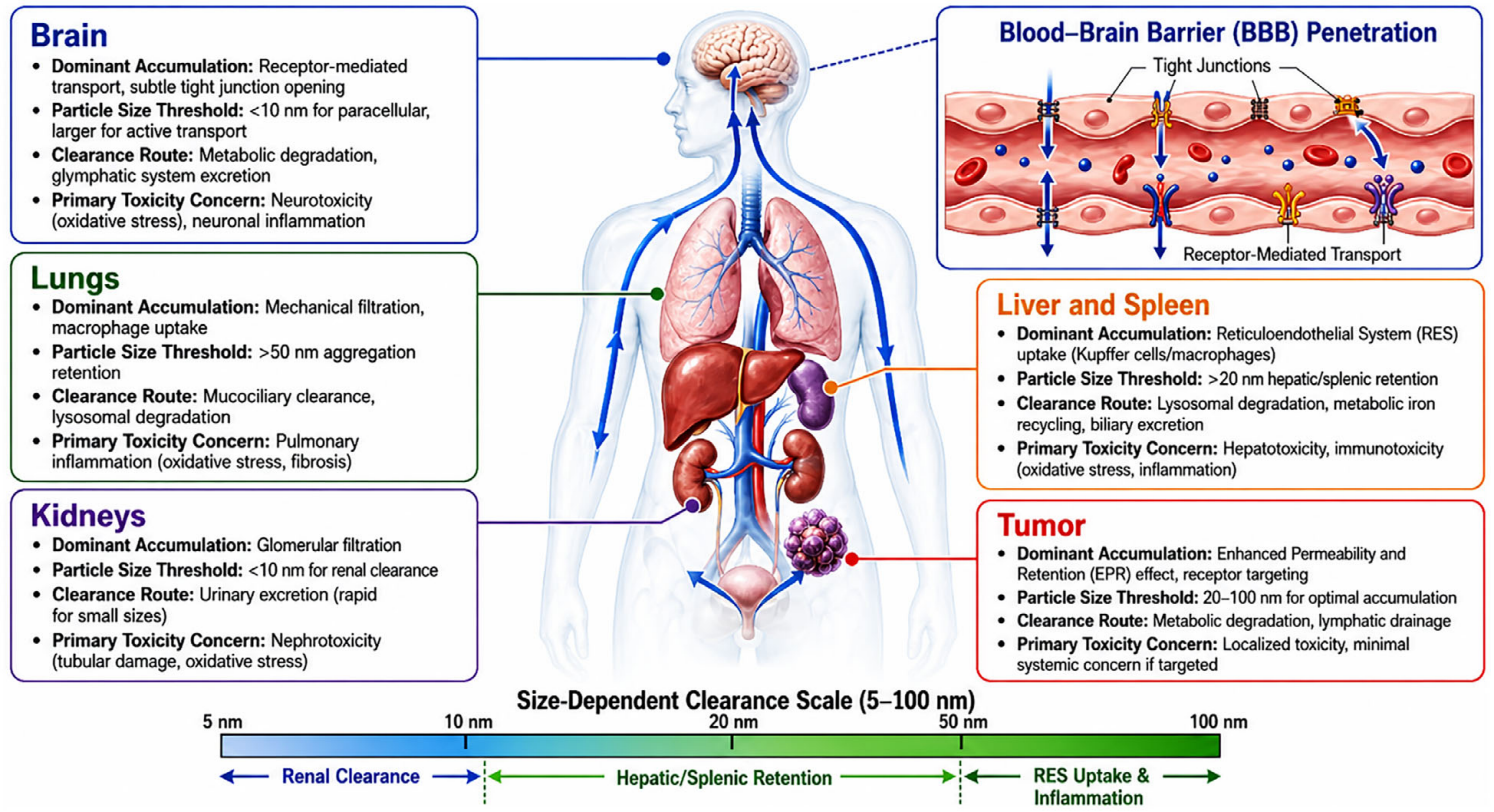
Systemic biodistribution, toxicity, and clearance pathways of spinel ferrite nanoparticles, highlighting organ‐specific accumulation, size‐dependent clearance, and principal toxicity concerns.

Figure 16 illustrates the systemic biodistribution, organ‐specific accumulation, and clearance pathways of spinel ferrite nanoparticles following systemic administration, highlighting the decisive role of particle size, surface chemistry, and transport mechanisms. Figure 16 contrasts the mechanistic pathways of ROS generation in spinel ferrites, demonstrating that the doped system (A) outperforms traditional Fenton and vacancy‐mediated approaches. Under AMF stimulation, this synergistic 4f‐3d coupling yields the highest ROS flux (B) and exhibits superior sustained kinetics (C), overcoming the saturation and slow reaction rates typical of undoped formulations. The detailed discussion of these mechanisms is provided in the subsequent sections.

#### 7.3.2 Long‐Term and Chronic Toxicity Considerations

Short‐term cytotoxicity assays alone are insufficient to predict long‐term biological safety. Recent in vivo studies extending beyond 12–24 weeks reveal that ferrite nanoparticles predominantly accumulate in the liver and spleen, followed by gradual biodegradation and clearance via iron metabolic pathways. However, prolonged exposure or repeated dosing may induce low‐grade inflammation, oxidative stress, or subtle organ dysfunction. Chronic exposure studies are also relevant for occupational safety during large‐scale nanoparticle manufacturing. These findings emphasize the importance of extended observation periods, repeated‐dose studies, and comprehensive histopathological analysis when assessing ferrite nanoparticle safety for clinical translation [300, 301, 302, 303].

#### 7.3.3 Immunogenicity and Genetic Toxicity Assessments

Beyond cytotoxicity, ferrite nanoparticles may elicit immune and genetic responses. Immunogenicity assessments commonly evaluate cytokine profiles (such as IL‐6, TNF‐α, IL‐1β), revealing that surface chemistry and protein corona composition strongly influence inflammatory signaling (IL‐6<2x baseline for PEG‐coated vs. 5x for bare CoFe_2_O_4_). Well‐coated ferrites generally induce minimal immune activation, whereas bare or defect‐rich surfaces may trigger macrophage‐mediated responses via NF‐κB pathways. Genotoxicity studies, including comet assays and micronucleus tests, indicate that DNA damage is typically associated with excessive ROS generation (tail moment <10 at <100 µg/mL) rather than direct nanoparticle–DNA interaction. While most ferrites are classified as non‐mutagenic at therapeutic doses (<50 µg/mL), systematic genetic toxicity screening remains essential, particularly for defect‐engineered and doped systems like Tb^3+^‐CoZn ferrites [296, 300, 301, 302].

#### 7.3.4 Organ‐Specific Toxicity Considerations

Ferrite nanoparticle biodistribution is highly organ‐dependent. The liver acts as the primary accumulation site (60%–80% injected dose at 24 h), where Kupffer cells internalize nanoparticles and facilitate degradation through iron metabolic pathways (ferritin/hepcidin recycling, clearance half‐life ∼30 days). The kidneys contribute to clearance primarily for ultrasmall nanoparticles (<8 nm hydrodynamic diameter), with toxicity thresholds closely linked to surface charge (zeta > ‐20 mV reduces glomerular filtration stress). Penetration of the blood–brain barrier is generally limited (<1% ID) but may occur under pathological conditions (e.g., glioma disruption) or via transferrin receptor targeting, necessitating careful neurological safety assessment via MPTP models. Understanding organ‐specific accumulation and clearance mechanisms is crucial for defining safe dosage windows (≤5 mg Fe/kg) and minimizing off‐target effects like hepatotoxicity or nephrotoxicity [179, 180, 181, 300, 301, 302].

#### 7.3.5 Biodegradation and Clearance Mechanisms

Biodegradation of ferrite nanoparticles proceeds primarily through a combination of lysosomal dissolution, oxidative transformation, and enzymatically mediated processing following cellular internalization. Once sequestered within acidic lysosomal compartments (pH 4.5–5.5), ferrite surfaces undergo proton‐assisted dissolution, promoting the release of Fe^2+^/Fe^3+^ ions (rate: 0.1–1 nm/day) and, in doped systems like CoFe_2_O_4_ or Tb‐CoZn ferrites, secondary metal ions. These released species are subsequently integrated into endogenous iron metabolic pathways, stored in ferritin complexes (capacity ∼4500 Fe atoms), or eliminated via renal (<8 nm particles) and hepatobiliary clearance routes (half‐life ∼30 days) [300, 301, 302, 304]. The degradation kinetics are highly sensitive to crystallinity, particle size, defect density, and surface functionalization, with defect‐rich or doped ferrites exhibiting accelerated dissolution (2–3x faster) due to lattice distortion and enhanced surface reactivity. Conversely, protective coatings such as silica or polymer shells (PEG, 5–10 chains/nm^2^) can significantly retard degradation by 4–6 weeks, prolonging functional lifetime but potentially delaying clearance. While degradation products are generally well tolerated at therapeutic doses (≤5 mg Fe/kg), uncontrolled ion release (Co^2+^ >100 µM) may elevate oxidative stress (GSH depletion) and inflammatory responses (IL‐6>2x baseline) [302, 305]. Reported in vivo clearance times range from 4–6 weeks (Fe_3_O_4_, MnFe_2_O_4_) to 12 weeks (CoFe_2_O_4_), depending on formulation and dosing frequency. Therefore, precise tuning of degradation kinetics via sol–gel processing parameters and shell engineering is critical for achieving a balance between sustained therapeutic performance (SAR >400 W/g) and long‐term biosafety, particularly for your Tb^3+^‐substituted Co‐Zn ferrite nanoparticles [177, 181, 302].

## 8 Biomedical Applications

A critical limitation in the current development of ferrite‐based nanomaterials lies in the lack of optimization of key functional parameters under application‐relevant conditions. For instance, although high specific absorption rate (SAR) values (>500 W/g) have been reported for magnetic hyperthermia, these are often achieved under nonclinical magnetic field conditions, limiting practical applicability. Similarly, while high relaxivity values are desirable for MRI contrast enhancement, achieving r_2_>100 mM^−1^s^−1^ with long‐term colloidal stability in physiological environments remains challenging. In drug delivery systems, although high loading efficiencies (>70%) are reported, controlled and stimuli‐responsive release under in vivo conditions is still not fully realized. These gaps highlight the need for a more integrated approach that simultaneously optimizes magnetic performance, stability, and biocompatibility.

### 8.1 Magnetic Resonance Imaging (MRI) Contrast Agents

Magnetic Resonance Imaging (MRI) stands as one of the most dynamic and versatile diagnostic modalities in modern medicine, distinguished by its noninvasive nature, high spatial resolution, and superior soft tissue contrast. While MRI provides anatomical detail, its diagnostic sensitivity can be significantly amplified through the use of contrast agents [306]. In this context, ferrite‐based nanomaterials have emerged as a transformative class of contrast agents, engineered to modify the intrinsic magnetic properties of tissues; specifically, the proton relaxation times thereby enhancing diagnostic precision and flexibility [307].

**FIGURE 17:**
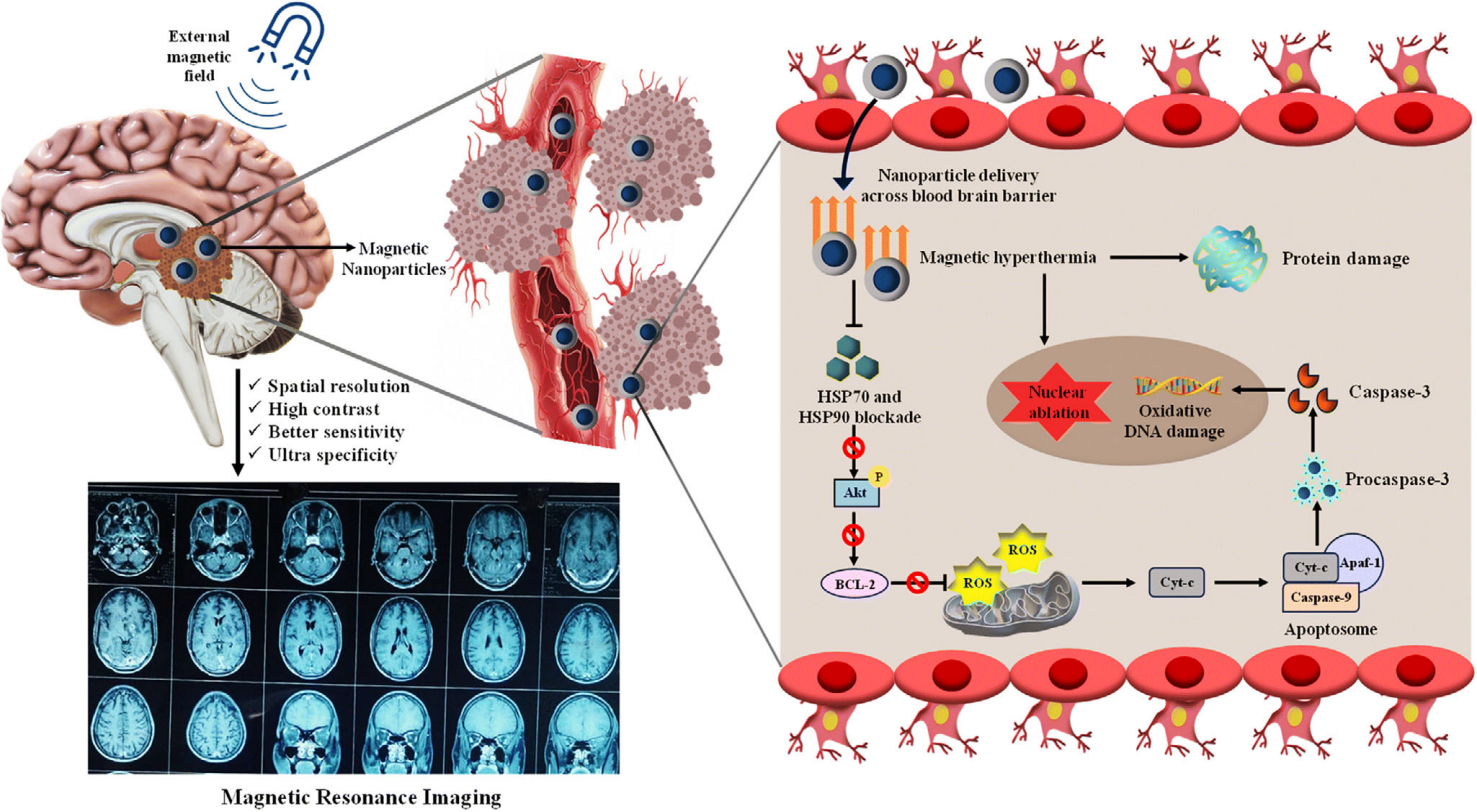
Representation of the dual role of magnetic nanoparticles in brain tumor theranostics. The diagram illustrates their function as high‐resolution MRI contrast agents and therapeutic mediators inducing magnetic hyperthermia, which triggers ROS‐mediated oxidative DNA damage and HSP blockade. Reproduced under the terms of the CC‐BY Creative Commons Attribution 4.0 International license. (http://creativecommons.org/licenses/by/4.0/) [308]. Copyright 2025, The Authors, published by Royal Society of Chemistry.

The development of these nanoprobes has moved beyond simple contrast enhancement toward multifunctional utility. As illustrated in Figure 17, the design strategy for next‐generation MRI agents involves the integration of a magnetic core (such as gold‐coated or ferrite nanoparticles) with various functional moieties [308]. This schematic demonstrates how a single nanoplatform can be engineered for targeted multimodal imaging by conjugating targeting ligands (e.g., antibodies, peptides, or aptamers) to the surface. This allows for the site‐specific accumulation of the contrast agent at the target tissue, such as a tumor, while simultaneously enabling complementary imaging techniques like nuclear or optical imaging alongside MRI.

For instance, ultrasmall MnFe_2_O_4_ nanoparticles (∼5 nm) have demonstrated enhanced longitudinal relaxivity (*r*_1_ ≈ 8–12 mM^−1^s^−1^), making them effective T_1_ contrast agents [309]. Similarly, Zn‐doped Fe_3_O_4_ nanoparticles have been reported to exhibit improved T_2_ relaxivity due to increased saturation magnetization, enabling superior contrast enhancement compared to conventional iron oxide systems [310]. Surface‐functionalized Fe_3_O_4_ nanoparticles coated with dextran or PEG have also shown prolonged circulation time and enhanced imaging efficiency in in vivo studies [311]. High‐performance MRI contrast agents require relaxivity values typically exceeding 100 mM^−1^s^−1^ (r_2_) or ∼10 mM^−1^s^−1^ (r_1_), which are strongly influenced by particle size, surface coating, and magnetic moment [312]. A wide spectrum of ferrite nanomaterials, including superparamagnetic iron oxides (SPIONs) and paramagnetic manganese oxides, is currently being explored to optimize these capabilities [307]. For instance, core‐shell architectures such as carbon‐coated iron oxide nanoparticles (Fe_3_O_4_@C) have shown promise for in vivo imaging of the liver and kidney, providing enhanced stability and biocompatibility [313]. Similarly, manganese‐based ferrites have been exploited for the specific imaging of tumor microenvironments. Surface engineering plays a pivotal role in this performance; PEGylated manganese‐zinc ferrite nanoparticles have been designed to serve as high‐contrast MRI agents by preventing agglomeration and extending circulation time [314, 315]. Furthermore, functionalization with citric acid has been utilized to modulate pH responsiveness, with studies revealing that such acidic coatings can significantly enhance biocompatibility and colloidal stability, rendering them suitable as high‐performance MRI probes [316, 317].

Despite these advancements, the clinical translation of ferrite‐based MRI agents faces challenges related to long‐term biocompatibility, biodistribution, clearance pathways, and potential off‐target accumulation. Addressing these issues requires a profound understanding of the magnetic effects and mode of action of these nanoparticles within various organ systems [308]. Future research efforts must focus on developing stimuli‐responsive contrast agents with optimized relaxivity and predictable biological fates. Moreover, the integration of artificial intelligence (AI)‐assisted image analysis and predictive labeling holds the potential to further revolutionize this field, enhancing the clinical utility and precision of nanoparticle‐based MRI platforms in the era of personalized medicine [318, 319]. In MRI, contrast arises from changes in longitudinal and transverse relaxation times; ultrasmall manganese or zinc ferrites can be engineered to act as positive (T_1_) agents, while larger, highly magnetic spinel ferrites typically function as negative (T_2_) agents that strongly shorten T_2_ and T_2_*. By tailoring core composition (e.g., MnFe_2_O_4_, ZnFe_2_O_4_, CoFe_2_O_4_), crystallinity, and particle size, it is possible to maximize relaxivities r_1_ and r_2_ and, in some cases, even realize dual‐mode T_1_/T_2_ behavior for more accurate diagnosis [116, 320, 321].

Dual‐modal T_1_/T_2_ ferrites, such as doped Mn‐Zn variants, further amplify diagnostic precision by combining positive and negative contrast for ratiometric imaging, while integration with targeting ligands (e.g., folic acid) enables tumor‐specific accumulation. These attributes position ferrite nanoparticles as gadolinium‐free platforms for theranostics, where MRI guidance couples with hyperthermia or drug release, heralding a new era in molecular imaging [322].

### 8.2 Drug Delivery & Targeted Therapy

The clinical efficacy of conventional chemotherapy is often compromised by nonspecific biodistribution, rapid renal clearance, and severe systemic toxicity, which limits the achievable dose at the tumor site. Ferrite nanoparticles have emerged as robust candidates for magnetic drug targeting (MDT), a strategy that utilizes external magnetic fields to guide drug‐loaded nanocarriers to specific pathological sites, thereby concentrating therapeutic agents in the diseased tissue while minimizing exposure to healthy organs.

Historically, pharmacotherapy has relied on conventional drug delivery systems (CDDS), where therapeutic agents are administered via oral, nasal, mucosal, or parenteral routes. While foundational, these traditional methods are often plagued by significant pharmacokinetic limitations, including poor solubility, rapid renal clearance, and enzymatic degradation. A critical drawback of CDDS is the nonspecific biodistribution of free drugs; therapeutic agents are distributed randomly throughout the body, often failing to reach the pathological site in effective concentrations while simultaneously accumulating in healthy tissues. This “off‐target” effect not only diminishes therapeutic efficacy but also induces severe systemic toxicity, a major hurdle in the treatment of chronic diseases [323, 324, 325].

The pharmaceutical industry has faced substantial challenges in overcoming these barriers. The development of new chemical entities (NCEs) is an intricate, capital‐intensive, and time‐consuming process, yet it is estimated that approximately 40% of drug candidates fail during clinical trials [326, 327]. These failures are frequently attributed to unpredictable toxicity profiles and poor bioavailability in humans rather than a lack of intrinsic potency. To address these limitations, early advancements focused on controlled‐release systems such as hydrogels, erotic matrices, and osmotic pumps designed to sustain drug release over time [328, 329]. However, these first‐generation engineered systems often struggled with issues of drug aggregation and lacked the ability to actively target specific cell populations.

**FIGURE 18:**
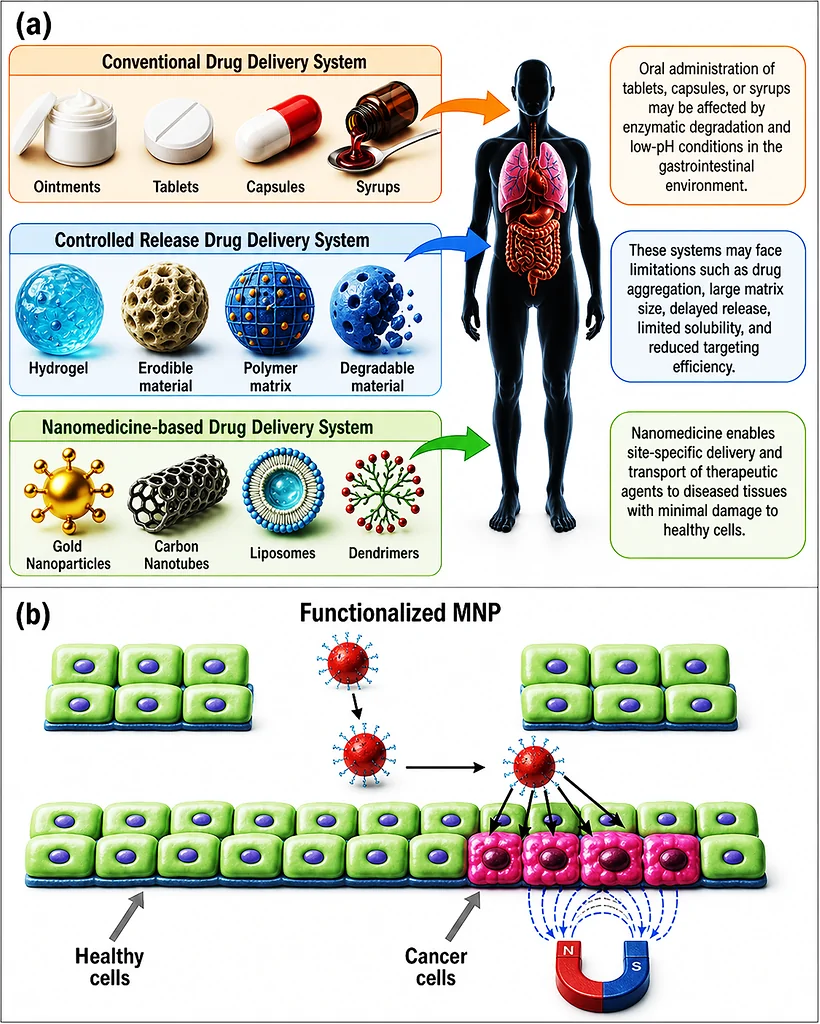
(a) Comparison of conventional, controlled‐release, and nanomedicine‐based drug delivery systems, highlighting the limitations of nonspecific drug administration and the advantages of site‐specific nanomedicine‐mediated delivery. (b) Schematic illustration of functionalized magnetic nanoparticles (MNPs) for magnetically guided cancer targeting, showing preferential accumulation at cancer cells under an external magnetic field while minimizing interaction with healthy cells.

Figure 18a schematically illustrates this paradigm shift in biomedical administration. It contrasts the mechanism of traditional medication, where free drugs circulate systemically causing collateral damage to healthy organs and cells, against modern nanomedicine approaches [330]. In the latter, nanocarriers (such as ferrite nanoparticles) are utilized to transport medications directly to specific pathological sites. This transition from the micro‐ to the nano‐scale revolutionizes therapy by protecting the therapeutic cargo from physiological degradation and ensuring high local concentrations at the disease site, thereby maximizing the therapeutic index while minimizing systemic harm [246, 331]. Figure 18b depicts the utilization of an external magnetic field to guide drug‐loaded magnetic nanoparticles (MNPs) deep into specific pathological tissues [332]. This active targeting mechanism significantly enhances the local bioavailability of the therapeutic agent while minimizing off‐target accumulation in healthy organs. By facilitating precise delivery and controlled release, this approach not only mitigates the systemic toxicity associated with conventional chemotherapy and radiotherapy but also effectively circumvents multidrug resistance (MDR) mechanisms in cancer cells, allowing for effective treatment at reduced dosages [333].

Ferrite nanoparticles have emerged as powerful drug delivery vehicles because their magnetic cores, tailorable surfaces, and tunable sizes allow spatiotemporal control over payload transport, release, and therapeutic action. In contrast to conventional systemic chemotherapy, which exposes both healthy and diseased tissues to cytotoxic agents, ferrite‐based nanocarriers can be engineered to concentrate drugs at pathological sites while minimizing off‑target accumulation, thereby improving therapeutic index and reducing dose‐limiting side effects [122, 334, 335]. For systemic administration, drugs are typically loaded onto or into ferrite nanocarriers via physisorption, electrostatic interaction, covalent conjugation, or encapsulation in polymeric, lipid, or mesoporous shells surrounding the magnetic core. Hydrophilic polymers (PEG, PVP, chitosan, and gelatin), lipids, or mesoporous silica are widely used to create core–shell architectures that provide high loading capacity, protect labile drugs, and enable controlled or stimuli‐responsive release in response to pH, redox conditions, enzymes, or temperature. For example, camptothecin‐loaded holmium ferrite nanocarriers achieve high loading (∼90%), sustained release, and enhanced anticancer activity, illustrating how ferrite cores can be integrated into multifunctional drug reservoirs [37, 276, 336, 337].

Targeting can be achieved through a combination of passive, magnetic, and molecular strategies, often used synergistically. Passive targeting exploits the enhanced permeability and retention (EPR) effect, whereby nanoscale ferrite carriers (typically 50–150 nm) preferentially accumulate in tumor tissue due to leaky vasculature and poor lymphatic drainage [338, 339]. Magnetic targeting, or magnetophoresis, uses external static magnetic field gradients to steer and retain drug‐loaded ferrite nanoparticles at specific anatomical sites, thereby increasing local drug concentration and reducing systemic exposure; this approach is particularly promising for tumors or lesions located within a few centimeters of an applied magnet [37]. Active molecular targeting is accomplished by decorating the nanoparticle surface with ligands such as antibodies, peptides (e.g., RGD), aptamers, or small molecules (e.g., folic acid), which bind overexpressed receptors on cancer cells or tumor vasculature to promote receptor‐mediated uptake and intracellular delivery [340, 341]. For instance, Fe_3_O_4_ nanoparticles functionalized with folic acid have demonstrated targeted delivery to cancer cells via receptor‐mediated endocytosis, significantly enhancing drug uptake [342]. In another study, PEGylated ZnFe_2_O_4_ nanoparticles loaded with doxorubicin showed controlled pH‐responsive drug release, improving therapeutic efficacy while minimizing systemic toxicity [343, 344]. Magnetic guidance of NiFe_2_O_4_‐based nanocarriers has also enabled site‐specific drug accumulation under external magnetic fields [345, 346]. Effective drug delivery systems demand high loading efficiency (>70%) and controlled release kinetics, which are governed by surface functionalization and nanoparticle stability in physiological environments [347].

Beyond serving as passive carriers, ferrite nanoparticles can actively participate in therapy through magnetically or ROS‐mediated mechanisms, giving rise to magneto‐chemotherapy and chemo–theranostic platforms [139, 246]. Under alternating magnetic fields, ferrite cores generate localized heat (magnetic hyperthermia), which can (i) increase tumor perfusion and cell membrane permeability [348], (ii) accelerate drug release from thermosensitive shells [225], and (iii) synergistically sensitize cancer cells to chemotherapeutics [349]. In parallel, specific compositions such as NiFe_2_O_4_ or Fe_3_O_4_ can potentiate drug‐induced apoptosis by elevating intracellular reactive oxygen species, modulating p53 and caspase pathways, and overcoming chemoresistance, as demonstrated for doxorubicin combinations. These multifunctional ferrite platforms enable integrated imaging, guidance, triggered release, and combination therapy within a single nanoconstruct, positioning them as promising candidates for precision oncology and next‐generation targeted therapeutics.

### 8.3 Magnetic Hyperthermia for Cancer Therapy

A critical analysis of the past three decades of magnetic hyperthermia (MHT) research highlights a significant divergence between clinical implementation and material science innovation. Current clinical trials, particularly for glioblastoma and prostate cancer, continue to rely on spherical iron oxide nanoparticles synthesized via hydrolytic routes. While these formulations have established a safety profile suitable for regulatory approval, their heating efficiency quantified by the specific absorption rate (SAR) remains modest compared to state‐of‐the‐art materials. Chang et al. herein underscores that the field is transitioning towards next‐generation heat mediators synthesized via non‐hydrolytic thermal decomposition. These methods yield superior crystallographic integrity and allow for precise morphological control. Specifically, anisometric shapes such as nanocubes, octopods, and nanoflowers have demonstrated SAR values orders of magnitude higher than commercial standards (e.g., Resovist) [350]. However, a significant translational gap remains: the scalability of these complex, high‐performance nanostructures is limited compared to aqueous synthesis [351, 352]. Therefore, the immediate challenge for the materials community is not merely discovering higher SAR materials, but developing scalable, green synthesis protocols that bridge the gap between high‐performance laboratory prototypes and good manufacturing practice (GMP) standards [353, 354]. For example, CoFe_2_O_4_ nanoparticles with enhanced magnetic anisotropy have exhibited high specific absorption rate (SAR) values exceeding 500 W/g under clinically relevant alternating magnetic fields. Mn–Zn ferrite nanoparticles have also been reported to achieve efficient heating performance due to optimized Néel–Brownian relaxation mechanisms [5, 21]. Recent studies on core–shell structures such as CoFe_2_O_4_@MnFe_2_O_4_ have demonstrated improved thermal efficiency and stability, making them promising candidates for cancer hyperthermia therapy [355].

Magnetic hyperthermia is one of the most intensively explored therapeutic uses of ferrite nanoparticles, exploiting their ability to convert electromagnetic energy into heat when exposed to an alternating magnetic field (AMF) [21]. Superparamagnetic or ferrimagnetic spinel ferrites such as Fe_3_O_4_, MnFe_2_O_41_, CoFe2O_4_, and doped Zn‐ or Cu‐ferrites dissipate energy via Néel/Brownian relaxation and hysteresis losses, raising the local temperature of tumor tissue into the therapeutic window of 42°C–46°C, where cancer cells undergo apoptosis or necrosis while surrounding healthy tissue is largely spared [356, 357]. The heating efficiency is quantified by the specific absorption rate (SAR), which depends sensitively on particle size, anisotropy, magnetization, concentration, and AMF parameters (typically 100–500 kHz, 5–20 kA/m); optimized nanoferrites can reach SAR values >300 W/g, enabling effective treatment at low injected doses [358, 359]. Efficient magnetic hyperthermia requires ferrite nanoparticles with high saturation magnetization (>60–80 emu/g) and optimized magnetic anisotropy, enabling SAR values exceeding ∼300 W/g under clinically acceptable field conditions [360, 361].

A recurring limitation identified in pre‐clinical evaluations is the discrepancy between colloidal heating efficiency and in vivo performance. Standard calorimetric characterization in aqueous media frequently overestimates therapeutic potential by neglecting the complex biological microenvironment. Upon cellular internalization, magnetic nanoparticles (MNPs) often undergo lysosomal clustering and encounter a high‐viscosity intracellular milieu, factors that severely suppress Brownian relaxation mechanisms [362]. Recent studies indicate that heating efficiency can plummet by up to 90% post‐internalization. The future MNP design must prioritize “viscosity‐independent” heating mechanisms. Nanostructures dominated by Néel relaxation, such as specific core‐shell architectures (e.g., exchange‐coupled systems) or pre‐assembled magnetic chains, appear more resilient to these biological constraints [363, 364, 365]. Consequently, there is a pressing need to update standardization protocols to include viscosity‐dependent susceptibility measurements, ensuring that reported SAR values are predictive of intratumoral behavior rather than ideal solution conditions.

**FIGURE 19:**
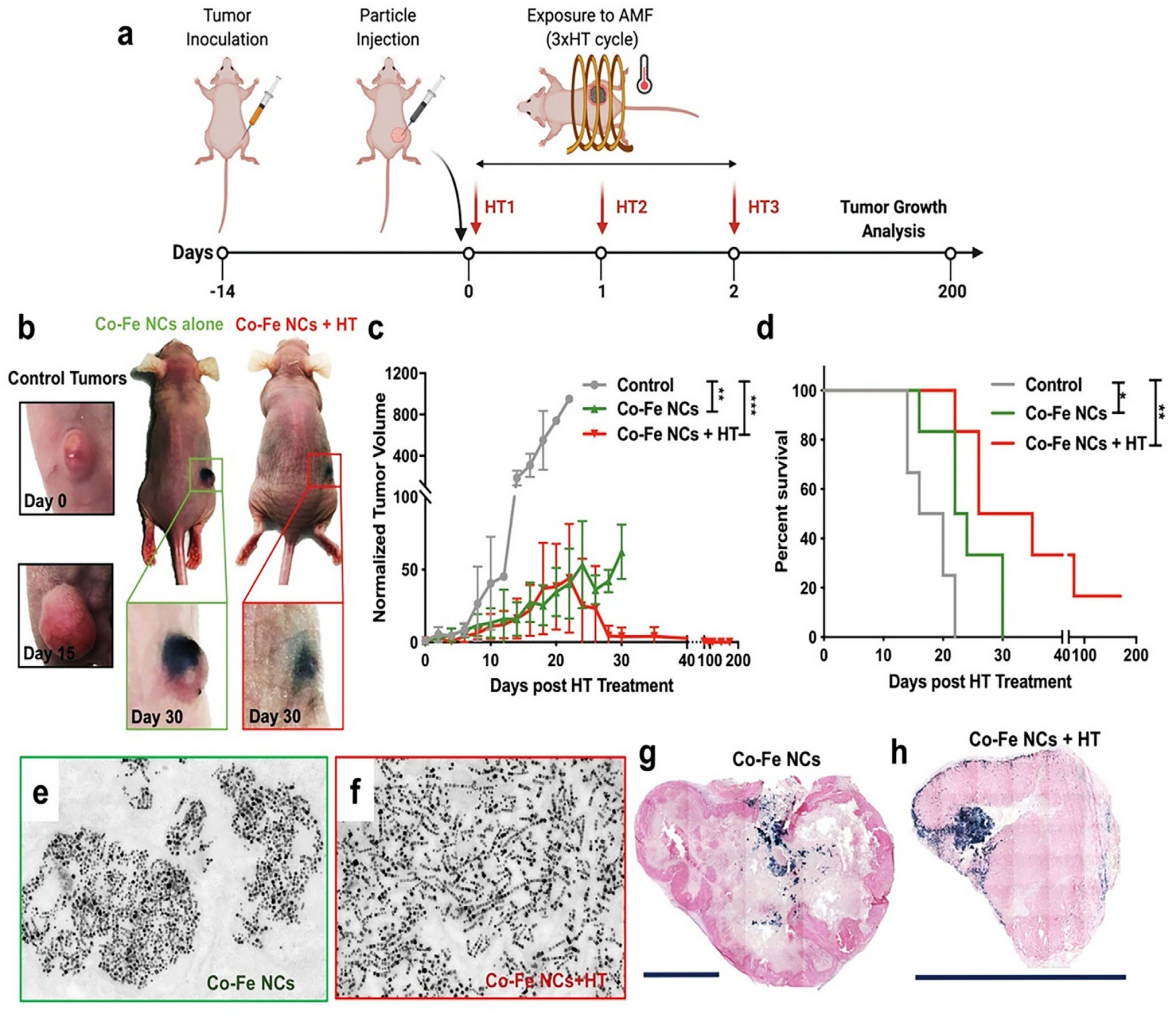
Long‐term in vivo therapeutic efficacy of Co–Fe nanocube‐mediated magnetic hyperthermia (HT). (a) Schematic representation of the treatment timeline. (b) Macroscopic observation of tumor progression; the Co–Fe NCs + HT group demonstrates complete tumor eradication by day 30, in contrast to the rapid growth observed in controls. (c) Tumor growth kinetics indicating durable remission with no recurrence observed up to 200 days post‐treatment in the hyperthermia group. (d) Kaplan–Meier survival analysis revealing a significant survival advantage for the Co–Fe NCs + HT group (100% survival at 200 days) compared to monotherapy and control groups (max survival < 30 days). (e, f) TEM micrographs of excised tumor tissues at day 30, confirming the persistence of chain‐like nanoparticle assemblies in both the Co–Fe NCs alone and Co–Fe NCs + HT (f) groups. (g, h) Histological assessment of tumor sections (Prussian blue for Fe, Fast Red for collagen/cells). The combined therapy group (h) exhibits extensive stromal ablation and nanoparticle retention compared to the nanoparticle‐only group (g). Scale bars: 0.5 cm. Reproduced under the terms of the CC‐BY Creative Commons Attribution 4.0 International license. https://creativecommons.org/licenses/by/4.0/ [366]. Copyright 2020, The Authors. Published by Wiley‐VCH GmbH.

Balakrishnan et al. did a long‐term in vivo study as shown in Figure 19. Figure 19a evaluated the therapeutic efficacy of Co–Fe nanocubes (NCs). While control tumors progressed rapidly, treatment with Co–Fe NCs alone delayed growth, whereas the combination of Co–Fe NCs + HT achieved complete tumor suppression by day 30 Figure 19b. This combined therapy resulted in durable remission with no recurrence for up to 200 days (Figure 19c), leading to a significantly extended disease‐free survival compared to the maximum 30‐day survival observed in control and monotherapy groups (Figure 19d). TEM analysis at day 30 confirmed the persistence of stable, long‐chain NC assemblies within the tissue (Figure 19e,f). Histological assessment (Figure 19g,h) revealed that HT treatment induced a swirling migration of these chains toward the tumor periphery, effectively ablating the protective collagenous stroma (Figure 19h). This mechanical disruption of the tumor microenvironment, which was absent in the group treated with NCs alone Figure 19g), likely contributed to the complete eradication of the tumor [366].

In cancer therapy, ferrite‐based magnetic hyperthermia is generally implemented either by intratumoral or systemic administration of a ferrofluid followed by AMF exposure to achieve spatially confined heating at the tumor site. Intracellular hyperthermia, achieved when nanoparticles are internalized into endosomes and lysosomes, is particularly effective, as the subcellular temperature rise disrupts organelle function, induces ROS generation, and activates programmed cell death pathways [367]. Compositional engineering such as Zn–Ca, Cu–Zn, or Mg‐doped ferrites allows tuning of Curie temperature for self‐limiting hyperthermia and modulation of biocompatibility, enabling nanoferrites that selectively kill cancer cells while showing negligible cytotoxicity toward normal fibroblasts in vitro [89, 246]. Clinically, iron oxide‐based magnetic fluid hyperthermia has already reached feasibility and early efficacy trials in glioblastoma and prostate cancer, where intratumoral nanoparticle injection combined with carefully controlled AMF exposure yields intratumoral temperatures of 42°C–49°C, acceptable safety profiles, and encouraging survival or local control when combined with radiotherapy or brachytherapy [368, 369].

A key strength of ferrite‐mediated hyperthermia lies in its capacity for synergistic multimodal therapy. Local heating enhances tumor perfusion, drug diffusion, and membrane permeability, thereby potentiating co‐delivered chemotherapeutics or immunomodulators, as demonstrated in combined MHT–chemotherapy regimens and MHT‐assisted chemo‐radiotherapy in preclinical glioblastoma models [370]. Moreover, magnetothermal heating can be coupled to Fenton‐ or Fenton‐like chemistry using iron‐containing ferrites, generating reactive oxygen species in situ and enabling ROS‐mediated tumor ablation and immunogenic cell death. These capabilities position ferrite nanoparticles as central components of “magnetotheranostic” platforms that integrate imaging, controlled heating, and adjuvant delivery, although translation still requires rigorous optimization of SAR under clinically acceptable AMF conditions and long‐term biodistribution and clearance studies [21, 352].

Table 6 summarizes key magnetic nanoparticles (MNPs) discussed in the review, categorized by synthesis method, morphology, and their respective specific absorption rate (SAR) values under defined alternating magnetic field (AMF) conditions.

**TABLE 6:** Comparative heating performance of magnetic nanomaterials for hyperthermia.

Material/Composition	Synthesis method	Morphology & size (nm)	Magnetic field conditions (H, f)	SAR (W/g)	Refs.
Fe_3_O_4_	Oxidative precipitation	Nanocubes (26 nm)	*H* = 35 kA/m, *f* = 70 kHz	170	[371]
Fe_3_O_4_	Aerial oxidation	Elongated (16 × 87 nm)	*H* = 43.7 kA/m, *F* = 109 kHz	370	[372]
Fe_3_O_4_	Thermal decomposition	Nanocubes (19 nm)	*H* = 16 kA/m, *f* = 300 kHz	620	[373]
Fe_3_O_4_	Thermal decomposition	Nanocubes (24 nm)	*H* = 16 kA/m, *f* = 300 kHz	650	[373]
Fe_3_O_4_	Solvothermal	Nanoflowers (21 nm)	*H* = 11 kA/m, *f* = 400 kHz	500	[374]
Fe_3_O_4_	Thermal decomposition	Octahedra (18 nm)	*H* = 24.5 kA/m, *f* = 400 kHz	124	[375]
Ag/Fe_3_O_4_	Solvothermal	Nanorods (7 × 41 nm)	*H* = 16 kA/m, *f* = 310 kHz	40	[376]
FeO/Fe_3_O_4_	Thermal decomposition	Nano‐octopods (47 nm)	*H* = 43.7 kA/m, *f* = 109 kHz	60	[360]
CoFe_2_O_4_	Thermal decomposition	Nanocubes (19 nm)	*H* × *f* = 3.36 × 10^9^ A/m s	880	[377]
CoFe_2_O_4_	Coprecipitation method	Nano Spheres (14 nm)	—	142	[378]
MnFe_2_O_4_	Thermal decomposition	Nanoflowers (19 nm)	*H* × *f* = 4.5 × 10^9^ A/m s	689	[379]
Zn_0.4_Mn_0.6_Fe_2_O_4_	Thermal decomposition	Nano spheres (15 nm)	*H* × *f* = 2.6 × 10^9^ A/m s	432	[380]
ZnFe_2_O_4_@Co_6_Fe_2_O_4_	Seed‐mediated growth	Core‐shell nanocubes (60 nm)	*H* × *f* = 1.87 × 10^11^ A/m s	10600	[381]
Co_6_Fe_2_O_4_@Mn_6_Fe_2_O_4_	Seed‐mediated growth	Core‐shell spheres (15 nm)	*H* × *f* = 1.87 × 10^11^ A/m s	2284	[382]
FeO@Fe_3_O_4_	Seed‐mediated growth	Core‐shell nanocubes (23 nm)	*H* × *f* = 4.8 × 10^9^ A/m s	310	[383]

Overall, the critical impact of morphological anisotropy and complex architecture on heating efficiency with nanocubes, octopods, and exchange‐coupled core‐shell systems demonstrates SAR values orders of magnitude higher than conventional spherical oxides. However, a significant translational gap remains between these high‐performance laboratory prototypes and clinical application. Overcoming this divide requires a paradigm shift towards developing viscosity‐independent heating mechanisms that retain efficacy within the intracellular environment, alongside the establishment of scalable, green synthesis protocols compliant with GMP standards. Ultimately, the future of magnetic hyperthermia lies in optimizing these advanced nanoferrites not only as superior heat mediators but as multifunctional agents within synergistic magnetotheranostic platforms, capable of delivering precise thermal ablation, mechanical tumor disruption, and targeted drug release.

### 8.4 Biosensing and Diagnostics

The integration of spinel ferrites (MFe_2_O_4_) into biosensing platforms represents a paradigm shift from their traditional role as passive carriers for magnetic separation to active components in signal transduction and amplification. While the superparamagnetic behavior of ferrite nanoparticles remains the cornerstone for enriching low‐abundance analytes from complex biological matrices (e.g., blood, urine), recent advances have pivoted toward exploiting their intrinsic enzymatic (nanozyme), electrochemical, and optical properties to push the limits of detection (LOD) into the attomolar range.

A critical innovation in the last decade is the utilization of ferrite nanoparticles as nanozymes, stable inorganic mimetics of biological enzymes. Unlike natural enzymes, which suffer from thermal instability and susceptibility to proteolytic digestion, ferrite nanostructures exhibit robust intrinsic peroxidase‐like activity. This catalytic capability arises from the Fenton and Fenton‐like reactions facilitated by the redox cycling between Fe^2+^ and Fe^3+^ on the particle surface [384]. In diagnostic applications, this allows ferrites to serve a dual function in Magneto‐ELISA (Enzyme‐Linked Immunosorbent Assay) formats: they act simultaneously as the capture support for antibodies and the signal generator that oxidizes colorimetric substrates in the presence of H_2_O_2_ [385]. Recent studies demonstrate that doping zinc or cobalt into the magnetite lattice (Zn_x_Fe_3‐x_O_4_) can tune the electronic structure, enhancing catalytic efficiency orders of magnitude beyond standard Fe_3_O_4_, thereby enabling the naked‐eye detection of cancer biomarkers and viral antigens at concentrations previously necessitating polymerase chain reaction (PCR) [386, 387].

The demand for early‐stage cancer detection and precise prognosis necessitates diagnostic tools that transcend the limitations of traditional assays. Magnetic sensing technologies have emerged as a formidable contender in this domain, offering capabilities that extend beyond the mere segregation and capture of analytes [388]. Over the past two decades, research has increasingly focused on exploiting the unique properties of magnetic nanostructures to engineer high‐performance Giant Magnetoresistance (GMR) sensors and electrochemical platforms. Giant magnetoresistance (GMR) biosensors, since the discovery of the GMR effect in ferromagnetic/nonmagnetic multilayers by Fert and Grünberg, have been adapted to detect biomolecular recognition events with remarkable sensitivity [389]. The working principle relies on the labeling of biomarkers (proteins, DNA) with magnetic nanoparticles (MNPs); binding events alter the resistance of the magnetic stack structure. A defining advantage of GMR platforms over optical counterparts is the virtual absence of magnetic background noise in biological media, as native tissues and fluids are diamagnetic. This high signal‐to‐noise ratio allows for the detection of low‐abundance targets in complex matrices. For example, Fe_3_O_4_ nanozymes have been widely used for colorimetric detection of glucose and hydrogen peroxide due to their peroxidase‐like activity [390, 391]. Similarly, CoFe_2_O_4_ nanoparticles have demonstrated enhanced catalytic efficiency in electrochemical biosensors, enabling sensitive detection of biomolecules [392]. Recent advancements in single‐atom ferrite nanozymes have further improved catalytic selectivity and sensitivity for biomedical diagnostics.

**FIGURE 20:**
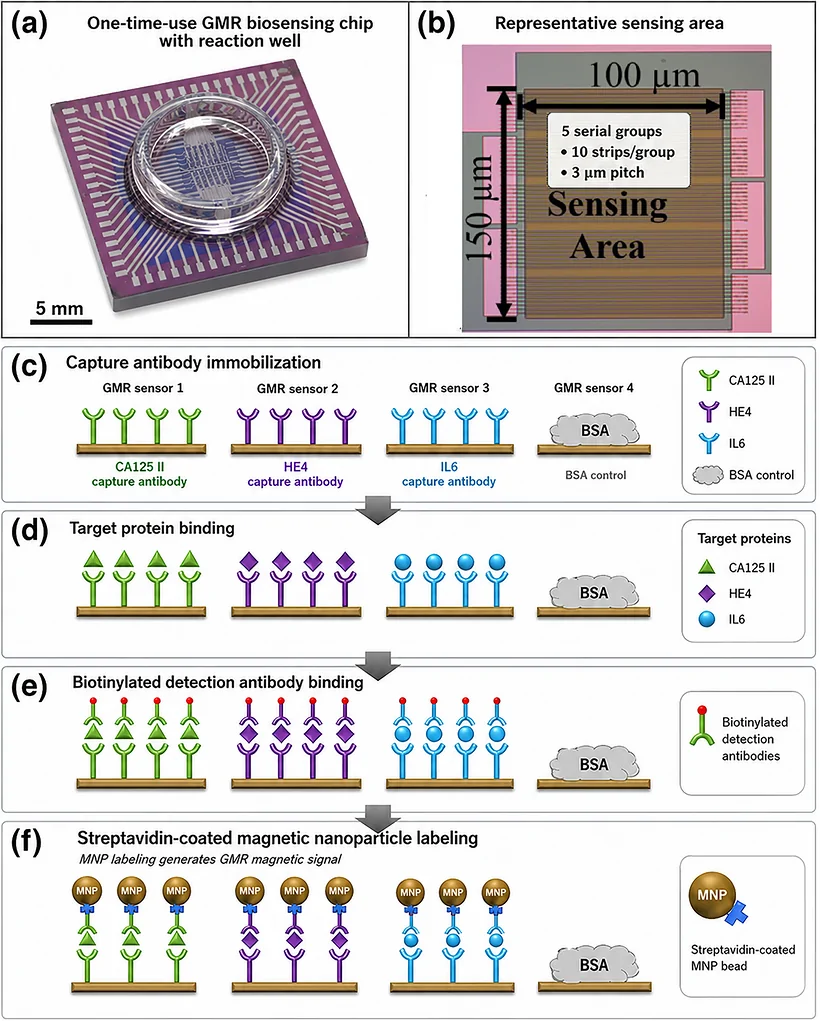
Multiplexed giant magnetoresistive (GMR) biosensor platform for magnetic nanoparticle‐assisted biomarker detection. (a) One‐time‐use GMR biosensing chip with reaction well. (b) Representative GMR sensing area showing sensor dimensions and strip arrangement. (c–f) Stepwise sandwich immunoassay workflow showing capture antibody immobilization for CA125 II, HE4, and IL6, selective target protein binding, biotinylated detection antibody recognition, and streptavidin‐coated magnetic nanoparticle labeled for GMR signal readout. BSA‐coated sensors serve as negative controls. Reproduced with permission [393]. Copyright 2018. Published by Elsevier.

Figure 20 highlights a significant leap in translating this technology to clinical utility. The study illustrates a portable, multiplexed GMR lab‐on‐a‐chip system designed for the serological screening of late‐stage serous ovarian cancer [393]. Unlike single‐analyte assays, this prototype simultaneously quantifies a panel of three distinct biomarkers: CA125 II, HE4, and IL6. The system demonstrates exceptional analytical performance, achieving Limits of Detection (LOD) of <3.7 U/mL for CA125 II and <7.4 pg/mL for both HE4 and IL6 (Figure 20a–f). This capability to detect picogram‐level concentrations in a portable format underscores the potential of GMR to decentralize cancer diagnostics, moving testing from centralized labs to point‐of‐care (POC) settings. Beyond ovarian cancer, the versatility of magnetoresistive sensing is evident in diverse architectures. Hybridization of commercial MNPs with gold nanoparticles has enabled the detection of human IgG with a detection limit of 13 pM (2 ng/mL), proving the robustness of magnetic‐labeled [394]. Furthermore, giant magneto‐impedance (GMI) sensors combined with maghemite nanoparticles have been successfully employed to quantify prostate cancer cells, validated via x‐ray fluorescence [395]. Recent material innovations, including spindle‐like Fe_3_O_4_, Ag‐decorated ferrites, and core‐shell nanostructures, continue to push the sensitivity limits of these devices [396, 397].

While GMR sensors require complex fabrication involving specialized magnetic wafers posing a barrier compared to simpler optical setups their compatibility with CMOS electronics facilitates the development of high‐density arrays necessary for high‐resolution genomic and proteomic screening. Ferrite‐based electrochemical sensors parallel to magnetic readout strategies, electrochemical biosensing utilizing magnetic nanomaterials offers a cost‐effective, rapid, and miniaturized alternative for POCT [398]. These sensors exploit the tunable redox properties and high surface area of ferrites to detect a broad spectrum of tumor markers, including CA19–9, HER2, MUC1, and CEA, using techniques such as voltammetry and electrochemical impedance spectroscopy (EIS) [399].

Structural engineering of ferrites has proven critical for enhancing sensor performance. For instance, heterogeneous hollow nanorods composed of α‐Fe_2_O_3_/Fe_3_O_4_‐Au have been synthesized to detect tumor antigen 125, demonstrating that complex morphologies can facilitate superior electron transfer [400]. Integration with 2D Mxenes has yielded robust nanohybrids (like CoFe_2_O_4_@Ag‐Mxene) for the rapid electrochemical isolation of HER2‐positive cells from whole blood [401]. Beyond cellular targets, Fe_3_O_4_ nanoparticles facilitate redox‐mediated signal amplification for multiplexed miRNA profiling [402, 403]. Furthermore, the tunable inverse spinel structure of NiFe_2_O_4_ optimizes surface electrocatalysis, proving highly effective for the sensitive detection of p53 and ovarian cancer biomarkers [401, 402, 403, 404].

Furthermore, preventing particle agglomeration is essential for reproducibility; dispersing CoFe_2_O_4_ on graphene oxide (GO) sheets not only stabilizes the nanostructures but also synergistically enhances conductivity for the detection of carcinoembryonic antigen (CEA) [405]. While some ferrite‐modified electrodes have been validated for pharmaceutical analysis (detecting oxycodone and codeine), their robust electrocatalytic surface properties are directly translatable to oncological applications [406]. Emerging candidates such as CuFe_2_O_4_ and MgFe_2_O_4_ are also gaining traction as cost‐effective alternatives to noble metals [407, 408].

Despite these advances, a disconnect remains between academic research and clinical adoption. While ferrite‐based sensors demonstrate superior performance in controlled laboratory environments, the transition to real‐world applications requires rigorous validation against complex biological interference and batch‐to‐batch consistency. Bridging this gap is the primary challenge for the next generation of magnetic diagnostic devices. The trajectory of ferrite‐based diagnostics is moving decisively toward miniaturized, lab‐on‐a‐chip, and lateral flow immunoassay (LFIA) devices. The magnetic contrast of ferrites is now being exploited in Magnetic Particle Imaging (MPI) and giant magnetoresistance (GMR) sensors, offering quantitative readouts that eliminate the “ambiguity of the faint line” often seen in visual rapid tests. However, the translation of these high‐performance materials into clinical reality hinges on solving the bio‐interface challenge: developing robust surface chemistries that prevent nonspecific protein adsorption (biofouling) while maintaining the accessibility of the catalytic or binding sites.

### 8.5 Antimicrobial and Antibacterial Agents

#### 8.5.1 Antimicrobial Agents

The escalating global crisis of antibiotic resistance has necessitated an urgent search for novel and effective antimicrobial agents. Conventional antibiotics are increasingly failing against multidrug‐resistant (MDR) bacterial strains, including notorious pathogens such as Methicillin‐resistant *Staphylococcus aureus* (MRSA) and drug‐resistant *Escherichia coli* [409]. In this context, nanotechnology has emerged as a promising frontier, offering metal‐oxide nanoparticles with unique physicochemical properties that enable potent and often multimodal antimicrobial action, presenting a viable alternative to traditional pharmaceuticals [410, 411]. Among the most intensively studied metal oxide nanomaterials for biomedical applications, ferrite nanoparticles (FNPs), particularly those with a spinel structure (Mfe_2_O_4_), where M is a divalent metal like Co, Ni, Zn, or Mn, have garnered significant attention due to their magnetic properties, high surface area, chemical stability, and demonstrated antimicrobial efficacy against a broad spectrum of pathogenic bacteria [214, 412].

Ferrite nanoparticles exhibit strong and often enhanced antimicrobial activity compared to their bulk counterparts, a phenomenon primarily attributed to their small size (10–100 nm) and exceptionally high surface‐to‐volume ratio. This high surface area maximizes the contact interface between the FNP and the bacterial cell, thereby promoting interaction and subsequent cell damage [413, 414]. The antimicrobial performance of FNPs is highly dependent on their physicochemical characteristics. Smaller nanoparticles generally exhibit superior antibacterial activity, as their increased surface energy and ability to infiltrate bacterial cells are enhanced [415]. Ultrafine magnetic nanoparticles have been shown to readily cross the bacterial cell wall/membrane. The divalent metal cation (M) within the spinel structure significantly influences antimicrobial potency. Doping with certain divalent metals, such as Cu^2+^, Zn^2+^, or Co^2+^, has been shown to enhance the antibacterial properties. The Zink (ZnFe_2_O_4_) and Cobalt (CoFe_2_O_4_) ferrites, as well as their mixed compositions (Zn‐Co or Ni‐Co ferrites), have been reported to exhibit potent activity against clinically relevant, drug‐resistant strains like *E. coli* and *S. aureus* [416, 417, 418]. The choice of the M‐site cation can modulate the release of bactericidal metal ions and the efficiency of reactive oxygen species (ROS) generation, both of which are key mechanisms of action. The coating FNPs with polymers (polyvinylpyrrolidone (PVP), polyethylene glycol (PEG)) or silica can enhance their biocompatibility, stability in biological media, and dispersibility, which is crucial for maximizing effective contact with bacterial cells and minimizing aggregation [419, 420]. Polymer coatings can also be functionalized to enable targeted delivery or to act as carriers for synergistic antimicrobial agents.

**FIGURE 21:**
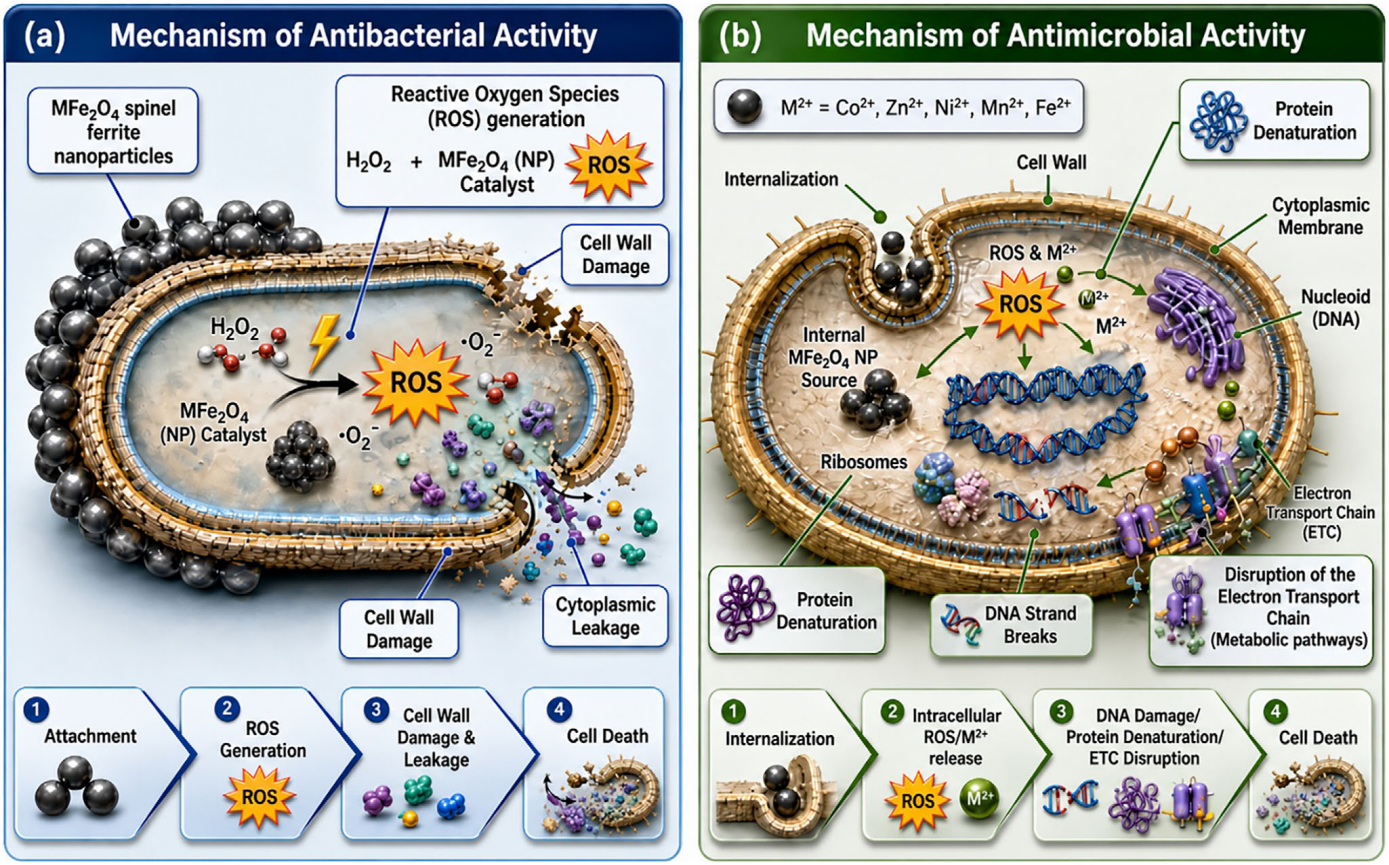
Schematic illustration of the antimicrobial action of spinel ferrite nanoparticles. (a) Antibacterial pathway showing the attachment of MFe_2_O_4_ nanoparticles to the bacterial surface, catalytic ROS generation, oxidative damage to the cell wall/cytoplasmic membrane, cytoplasmic leakage, and loss of cell viability. (b) Intracellular antimicrobial pathway involving nanoparticle internalization, ROS/M^2+^ release, DNA strand breaks, protein denaturation, disruption of the electron transport chain, impaired metabolic activity, and bacterial inactivation.

Figure 21a schematically illustrates the proposed antibacterial mechanism of spinel ferrite nanoparticles (MFe_2_O_4_). The initial interaction generally involves the attachment of nanoparticles to the bacterial surface, which may be assisted by electrostatic interactions between the nanoparticle surface and charged functional groups present on the cell envelope. Following surface contact, MFe_2_O_4_ nanoparticles can act as catalytic sites for the generation of reactive oxygen species (ROS), including superoxide radicals, hydroxyl radicals, and other oxidative intermediates, particularly in the surrounding microenvironment. These ROS impose oxidative stress on the bacterial envelope, promoting lipid peroxidation and progressive disruption of the cell wall and cytoplasmic membrane. As membrane integrity deteriorates, permeability increases, leading to leakage of intracellular components and eventual loss of cellular viability [421, 422]. The extent of this bactericidal response can depend on the structural characteristics of the bacterial cell envelope. Gram‐negative bacteria are often reported to exhibit relatively higher susceptibility because their envelope contains a thinner peptidoglycan layer associated with an outer membrane rich in lipopolysaccharides. In contrast, Gram‐positive bacteria possess a thicker, multilayered peptidoglycan network, which can provide a more effective physical barrier against nanoparticle penetration and oxidative damage.

Figure 21b illustrates the intracellular antimicrobial pathway associated with membrane disruption and nanoparticle internalization. After entering the bacterial cell, Mfe_2_O_4_ nanoparticles may function as localized sources for ROS generation and, depending on composition and dissolution behavior, can release metal ions such as Co^2+^, Zn^2+^, Ni^2+^, Mn^2+^, or Fe^2+^. The combined action of ROS and released M^2+^ species can perturb multiple intracellular targets, including genomic DNA, proteins, ribosomes, and membrane‐associated respiratory components. Oxidative stress may induce DNA strand breaks and interfere with replication, while protein denaturation can impair essential enzymatic and metabolic functions. Disruption of the electron transport chain further compromises respiratory activity and cellular energy production. In this context, the antimicrobial response of ferrite nanoparticles is best viewed as a coupled, multistep process involving membrane damage, intracellular oxidative stress, ion‐mediated biochemical interference, genotoxic effects, protein dysfunction, and respiratory impairment [423].

**FIGURE 22:**
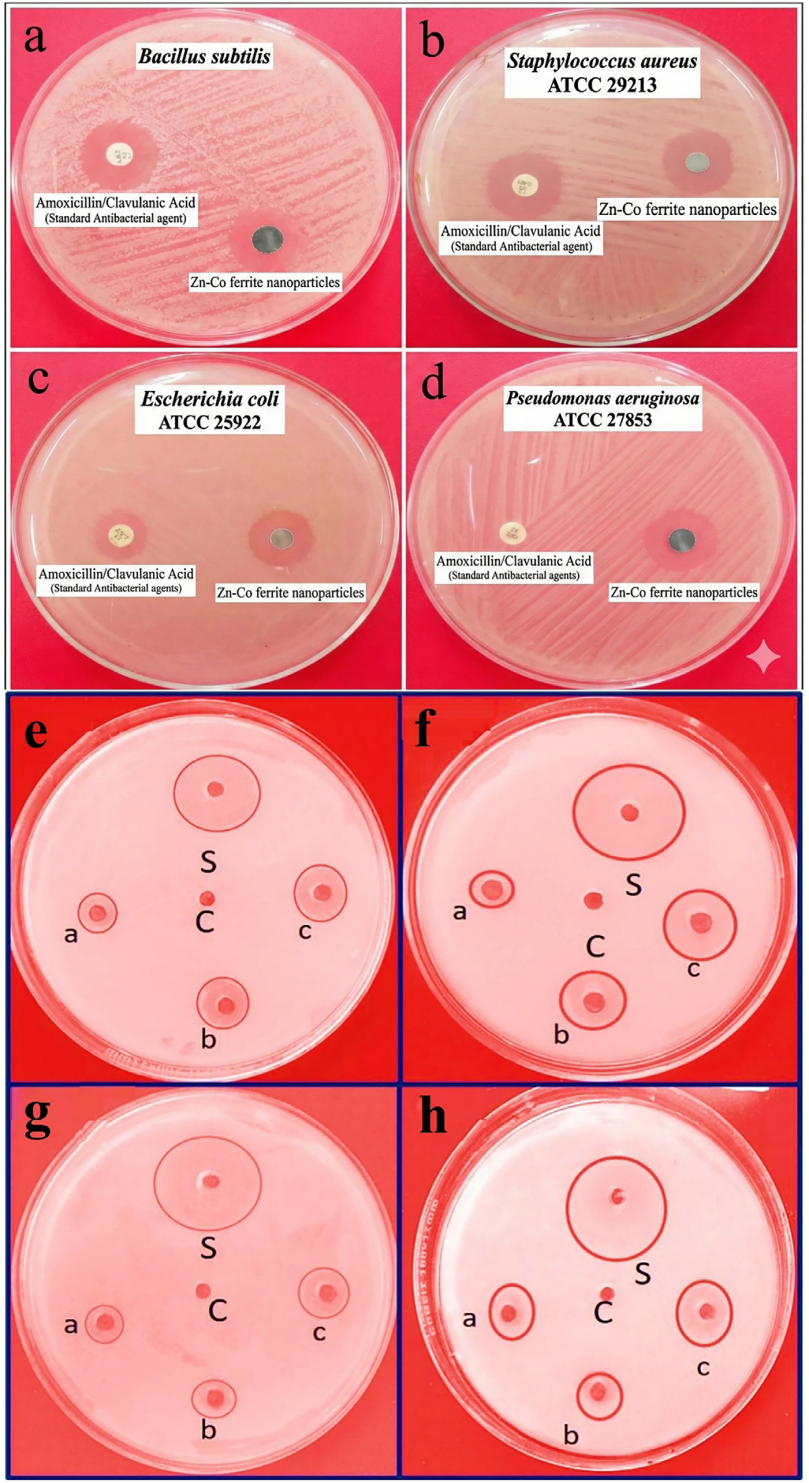
Antibacterial activity of irradiated zinc cobalt ferrite nanoparticles (150 kGy) demonstrating zones of inhibition against: (a) *Bacillus subtilis*, (b) *Staphylococcus aureus* (ATCC 29213), (c) *Escherichia coli* (ATCC 25922), and (d) *Pseudomonas aeruginosa* (ATCC 27853). Antibacterial activity of ZnFe_2_O_4_ nanoparticles against pathogenic bacterial strains: *S. aureus*, (f) *E. coli*, (g) *P. desmolyticum*, and (h) *K. aerogenes*. The labels denote: S = standard antibiotic; C = control; and a–c represent nanoparticle concentrations of 50, 100, and 150 µg/µL, respectively. (a–d) Reproduced under the terms of the CC‐BY Creative Commons Attribution 4.0 International license. https://creativecommons.org/licenses/by/4.0/ [424]. Copyright 2019, The Authors. Published by MDPI. E‐h) Reproduced with permission [423]. Copyright 2019, Published by Elsevier.

The antimicrobial efficacy of zinc cobalt ferrite nanoparticles was found to be dose‐dependent upon gamma irradiation (50, 100, and 150 kGy), culminating in peak activity at 150.0 kGy. As illustrated in Figure 22a–d, the irradiated samples exhibited substantial inhibition zones, particularly against *S. aureus* (16.0 mm) and *P. aeruginosa* (15.0 mm). This enhanced performance is directly attributed to the radiation‐induced structural refinement, specifically the reduction in crystallite size from 14.64 to 12.33 nm (as detailed in the “effect of ionizing radiation” section). The reduction in particle size leads to a significant increase in the specific surface area, maximizing the interfacial contact between the nanoparticles and the bacterial cell walls. This amplified surface interaction facilitates greater ROS generation and cellular uptake, thereby significantly elevating the bactericidal efficiency [424, 425]. The antibacterial potential of microwave‐assisted green‐synthesized ZnFe_2_O_4_ nanoparticles was evaluated against a panel of pathogenic strains, including *S. aureus*, *E. coli*, *P. desmolyticum*, and *K. aerogenes*, utilizing the agar well diffusion assay. As illustrated in Figure 22e–h, the nanoparticles displayed significant bactericidal activity relative to the positive control (ciprofloxacin). Notably, the expansion of the zones of inhibition correlates with increasing nanoparticle dosages (50, 100, and 150 µg/µL), suggesting a clear dose‐dependent antimicrobial mechanism [423].

The antimicrobial activity of ferrite nanoparticles (FNPs) is generally multimodal, exhibiting nonspecific and broad‐spectrum effects that make bacterial resistance development far less likely compared to conventional antibiotics. Several complementary mechanisms have been proposed to explain their bactericidal action [414, 426, 427]. The most widely accepted mechanism involves the generation of reactive oxygen species (ROS), including superoxide radicals ($\cdot O_{2}^{-}$), hydroxyl radicals (· OH), and hydrogen peroxide (H_2_O_2_). Due to their small size and high surface area, FNPs provide a large number of reactive sites, and the presence of surface‐exposed transition metal ions (Fe^2 +^ /Fe^3 +^) facilitates redox reactions in the biological environment [428]. These reactions trigger oxidative stress within bacterial cells, leading to multiple forms of molecular damage: 1) Lipid peroxidation, which compromises the structural integrity and fluidity of the cell membrane. 2) DNA damage, causing strand breaks, mutagenesis, and cell cycle arrest. 3) Protein oxidation, leading to enzyme inactivation and disruption of essential metabolic pathways. In certain ferrites such as copper ferrite (CuFe_2_O_4_), a Fenton‐like reaction may further enhance antibacterial efficiency by catalyzing the decomposition of H_2_O_2_ into highly reactive  · OH radicals, resulting in amplified oxidative damage [429, 430].

Electrostatic interactions play a vital role in the antibacterial effect of FNPs. The positively charged surfaces of many metal‐oxide nanoparticles (due to M^2 +^  or Fe^3 +^  cations) interact strongly with the negatively charged bacterial membrane components such as phospholipids and lipopolysaccharides. This interaction induces mechanical stress and membrane deformation, leading to the formation of pores or pits on the cell surface. The resultant loss of membrane integrity causes leakage of intracellular contents, including proteins, DNA, and essential ions (K^+^), culminating in cell lysis and death [431, 432]. Another significant mechanism involves the release of metal ions due to partial dissolution of FNPs under physiological or slightly acidic conditions. The liberated M^2 +^  and Fe^3 +^  ions can penetrate bacterial cells and interact with intracellular biomolecules. They tend to bind with sulfhydryl (─SH) groups in proteins, deactivating critical enzymes, and can also interfere with nucleic acid synthesis by binding directly to DNA or RNA [433]. The toxicity intensity largely depends on the type of dopant cation (e.g., Cu^2 +^, Zn^2 +^, or Co^2 +^) incorporated into the ferrite structure [434].

Current research efforts aim to enhance the antimicrobial efficacy of FNPs by employing synergistic and multifunctional strategies. Bacterial biofilms present a major clinical challenge, as their extracellular polymeric matrix significantly impedes antibiotic penetration and enhances resistance [435]. Functionalized or silver‐decorated ferrites have demonstrated pronounced anti‐biofilm activity, effectively disrupting established biofilms and inhibiting new ones from forming. This property highlights their potential for integration into medical coatings, wound dressings, and hospital surface materials. FNPs can be engineered to combine antimicrobial action with other therapeutic modalities [436]. Ferrite nanoparticles such as CuFe_2_O_4_ can efficiently absorb near‐infrared (NIR) radiation, converting it into localized heat that induces bacterial death through hyperthermia. When coupled with their intrinsic ROS‐generating (Fenton‐like catalytic) behavior, a strong synergistic antibacterial effect is achieved. The magnetic nature of FNPs enables external‐field control for targeted drug delivery. FNPs can be conjugated with antibiotics and magnetically directed to infection sites, thereby increasing local drug concentration while minimizing systemic toxicity [437].

For clinical and biomedical applications, the biocompatibility of FNPs is a key consideration. Despite their potent antibacterial function, excessive ROS generation or metal ion release can harm mammalian cells. Among ferrites, magnetite (Fe_3_O_4_) and zinc ferrite (ZnFe_2_O_4_) are generally preferred for biomedical use because of their relatively low cytotoxicity and the physiological compatibility of their constituent ions [412, 438]. Surface modification with biocompatible polymers (such as PEG, PVA) or inorganic shells (such as silica) is a common strategy to enhance safety and colloidal stability. These coatings prevent ion leaching, reduce nonspecific cellular interactions, and improve the overall therapeutic index of the nanoparticles [439].

Overall, the future efforts should prioritize robust, low‐cost, and scalable synthesis protocols, including plant‐mediated and other green routes, to achieve reproducible control over particle size, morphology, composition, and surface chemistry, which are critical for regulatory approval and clinical translation. Establishing standardized characterization and quality benchmarks will be essential to correlate structure–property relationships with antimicrobial performance and safety. Systematic in vivo studies are required to evaluate antibacterial efficacy in realistic infection models, along with detailed assessment of pharmacokinetics, biodistribution, metabolism, and clearance of FNPs over acute and chronic timescales. Such studies should also clarify their long‐term fate (biodegradation vs. persistence), immunological responses, and possible off‐target accumulation in critical organs. Rational surface engineering can enable multifunctional FNP platforms that integrate imaging (e.g., MRI contrast), magnetic hyperthermia or photothermal therapy, and targeted drug delivery with intrinsic antimicrobial action in a single “theranostic” system. Smart designs that exploit magnetic guidance, stimuli‐responsive drug release, and synergistic ROS or heat‐mediated killing could provide precision tools for real‐time diagnosis and treatment of difficult‐to‐treat infections and biofilms.

#### 8.5.2 Antibacterial Agents

The antimicrobial potential of spinel ferrite nanoparticles (SFNPs) represents a groundbreaking frontier in combating multidrug‐resistant (MDR) bacteria, whose rapid evolution undermines the efficacy of classical antibiotics. The theoretical basis for their action lies in multifaceted physicochemical interactions that disrupt bacterial physiology at multiple levels, thereby impeding adaptive resistance development. This comprehensive multimodal bactericidal mechanism combines oxidative stress induction, physical membrane interactions, and metal ion toxicity, each underpinned by intrinsic ferrite properties such as crystal structure, surface chemistry, and electronic configuration.

First, the prime mechanism involves the generation of reactive oxygen species (ROS) through Fenton and Fenton‐like catalytic reactions occurring at the ferrite surface or intracellularly after nanoparticle internalization. Ferrites containing transition metals with variable valence states (e.g., Fe^2+^/Fe^3+^, Cu^+^/Cu^2+^, and Mn^2+^/Mn^3+^) participate in redox cycling that converts metabolic hydrogen peroxide (H_2_O_2_) into highly reactive hydroxyl radicals (‐ OH), superoxide anions (‐ O_2_^−^), singlet oxygen (^1^O_2_), and other oxidative entities [440]. The narrow bandgap semiconductor nature of ferrites additionally promotes photo‐induced electron‐hole pair formation under UV/visible light, where valence band holes oxidize water molecules producing ─OH radicals while conduction band electrons reduce molecular oxygen to superoxide species. These ROS inflict severe oxidative damage by initiating lipid peroxidation within bacterial membranes, oxidizing critical thiol groups on proteins and enzymes, and causing DNA strand breaks and mutations [441, 442]. This oxidative onslaught culminates in cellular apoptosis or necrosis, overwhelming bacterial repair systems.

Complementing this, electrostatic and mechanical membrane disruption is a pivotal antibacterial strategy. Bacterial membranes are characteristically negatively charged due to ionized phospholipids and cell wall components such as lipopolysaccharides and teichoic acids [443]. Ferrites engineered with a positive surface charge exhibit strong electrostatic attraction, facilitating adsorption and clustering at the bacterial surface. This high local concentration triggers membrane depolarization, compromising the transmembrane potential essential for ATP synthesis and solute transport [444]. Moreover, ferroic nanoparticles with anisotropic geometries (e.g., nanorods, nanoplates) act as nanoscale knives, mechanically puncturing membranes to form pores that result in leakage of intracellular ions like K^+^, proteins, and nucleic acids, ultimately causing irreversible lysis. This physical mode of attack is independent of bacterial metabolic states, offering potent efficacy against dormant and persister cells resistant to conventional drugs [445, 446]. The third mechanistic pillar pertains to the controlled release of toxic metal ions from ferrite matrices under acidic biofilm microenvironments or intracellular conditions. Slow dissolution liberates divalent cations such as Zn^2^^+^, Cu^2^^+^, and Ni^2^^+^, which traverse nonspecific bacterial ion channels. Once internalized, these “soft” metal ions preferentially bind to thiol and imidazole groups within respiratory enzymes and DNA polymerases, disrupting electron transport, enzyme function, and nucleic acid replication. This ion interference leads to metabolic collapse and halted cell division [447, 448]. The toxicity spectrum and potency are tunable via compositional doping; for instance, zinc‐doped ferrites display broad‐spectrum antimicrobial efficacy linked to Zn^2^^+^ enzymatic inhibition and elevated ROS, while copper‐doped counterparts leverage redox‐active Cu ions and Jahn‐Teller distortions for enhanced catalytic ROS generation. Silver‐doped ferrites combine magnetic recoverability with potent silver ion bactericidal properties, enabling recycling and minimizing environmental release risks [449]. For example, CuFe_2_O_4_ nanoparticles have shown strong antibacterial activity against *E. coli* and *S. aureus* through ROS‐mediated membrane disruption [450, 451]. Similarly, ZnFe_2_O_4_ nanoparticles have demonstrated efficient biofilm inhibition due to their ability to generate superoxide radicals. Defect‐engineered ferrites with oxygen vacancies have further enhanced ROS production, improving their antimicrobial and anticancer efficacy [452, 453].

Beyond single‐cell killing, ferrite nanoparticles demonstrate exceptional anti‐biofilm capabilities. Biofilms, complex bacterial consortia embedded in an extracellular polymeric substance (EPS) matrix, pose considerable drug penetration barriers and infection persistence risks [454, 455]. The intrinsic magnetism of ferrites enables magnetically guided deep penetration into viscous EPS under applied magnetic fields (magnetophoresis), overcoming diffusion limits. Subsequent hyperthermia via alternating magnetic fields raises localized temperatures within biofilms, destabilizing EPS polymers and killing entrapped bacteria through thermal stress [456, 457, 458]. Additionally, continuous ROS generation chemically degrades EPS components, exposing bacteria to host immune mechanisms and co‐administered antibiotics. These mechanisms synergistically disrupt biofilms far more effectively than conventional treatments, with potential applications in wound healing, implant coatings, and nosocomial infection control [459, 460]. Nonetheless, employing ferrite nanoparticles clinically mandates addressing biocompatibility and selectivity challenges. While MRSA and E. coli eradication demonstrate promise, systemic toxicity from metal ion leaching or nonspecific ROS damage must be minimized. Biocompatible surface coatings such as chitosan and polyethylene glycol (PEG) provide steric and electrostatic shielding to host cells while allowing diffusion of antimicrobial species. Smart functionalization with pathogen‐specific ligands (e.g., bacteriophages, antimicrobial peptides) further enhances targeted bacterial adhesion, maximizing efficacy and minimizing off‐target effects [461, 462, 463, 464].

**FIGURE 23:**
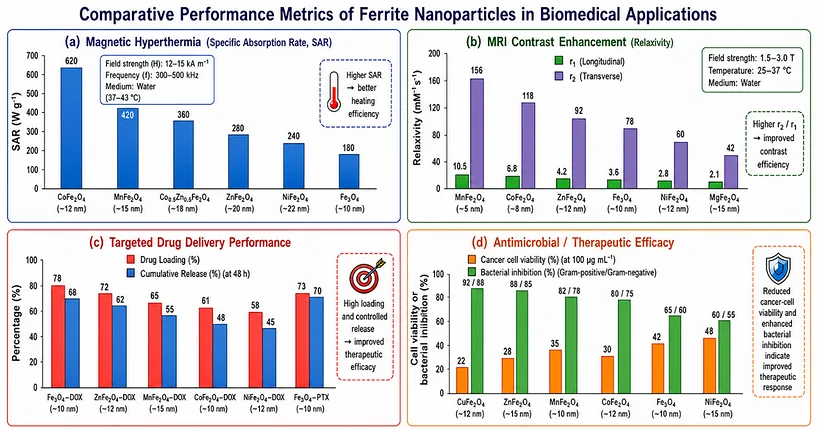
Comparative analysis of experimentally reported performance metrics of ferrite nanoparticles in biomedical applications: (a) specific absorption rate (SAR) for magnetic hyperthermia, (b) longitudinal/transverse relaxivity for MRI contrast enhancement, (c) drug loading and release efficiency for targeted drug delivery systems, and (d) therapeutic efficacy represented by cell viability or antimicrobial activity. Data are compiled from representative literature reports to highlight structure–property–performance relationships.

To provide a quantitative perspective on the performance of ferrite‐based nanomaterials, a comparative summary of key experimental metrics is presented in Figure 23. Parameters such as specific absorption rate (SAR), magnetic relaxivity, drug loading efficiency, and therapeutic activity are critically compared across different ferrite compositions. This analysis highlights the influence of structural parameters, including composition, particle size, and surface modification, on the overall biomedical performance.

To enable a clearer understanding of the relationship between material design and functional performance, it is essential to correlate the intrinsic physicochemical properties of ferrite nanomaterials with their application‐specific requirements. Parameters such as saturation magnetization, magnetic anisotropy, relaxivity, catalytic activity, and surface functionality play a decisive role in determining the efficiency of biomedical applications including hyperthermia, MRI contrast enhancement, and targeted drug delivery. Table 7 summarizes the key performance metrics, representative material systems, and existing limitations, providing a quantitative framework for evaluating current progress and identifying critical gaps for future development.

**TABLE 7:** Structure–property–performance requirements of ferrite nanomaterials for biomedical applications.

Application	Required property	Typical target value	Example material	Reported value	Limitation
Hyperthermia	SAR	>300 W/g	CoFe_2_O_4_	∼500 W/g	Heating efficiency varies with size
MRI	R_2_ relaxivity	>100 mM^−1^s^−1^	MnFe_2_O_4_	∼150 mM^−1^s^−1^	Stability in vivo
Drug delivery	Drug loading	>70%	Fe_3_O_4_–DOX	∼75%	Burst release
Antimicrobial	ROS efficiency	High	CuFe_2_O_4_	Strong inhibition	Cytotoxicity
Tissue engineering	Biocompatibility	>90% viability	Fe_3_O_4_ scaffolds	∼95%	Long‐term stability

A critical challenge in advancing ferrite nanomaterials toward clinical translation lies in achieving an optimal balance between material properties and functional performance. While high saturation magnetization and anisotropy enhance hyperthermia efficiency, they may compromise colloidal stability and biocompatibility. Similarly, materials exhibiting high relaxivity for MRI applications often require precise control over particle size and surface chemistry to ensure in vivo stability [347]. Despite significant progress, key performance gaps remain, including the need for higher SAR values under clinically safe magnetic fields, improved long‐term stability, reduced cytotoxicity, and scalable synthesis strategies. Addressing these challenges requires a rational design approach guided by quantitative structure–property–performance correlations [465].

In summary, the theoretical rationale behind ferrite nanoparticles as antimicrobials lies in harnessing their tunable physicochemical properties to deliver an integrated multimodal bactericidal attack. By combining ROS‐mediated oxidative damage, physical membrane disruption, and toxic ion interference with enhanced biofilm penetration and controlled selectivity, SFNPs constitute a versatile and powerful platform poised to address the escalating threat of multidrug‐resistant infections. Continuous advances in controlled synthesis, surface engineering, and mechanistic understanding will be imperative for translating this promising class of nanomaterials from bench to bedside.

### 8.6 Other Biomedical Applications

Ferrite‐based nanomaterials extend well beyond the classical triad of MRI, drug delivery, and hyperthermia, underpinning a broader portfolio of biomedical technologies that exploit their magnetic, catalytic, and electronic properties in increasingly sophisticated ways. These emerging applications range from tissue engineering and regenerative medicine to magnetically assisted surgery, bioseparation, and integrated theranostics, and collectively highlight how rationally engineered ferrites can function as active regulators of cellular microenvironments and biological processes rather than passive carriers alone.

#### 8.6.1 Tissue Engineering and Regenerative Medicine

In tissue engineering, ferrite nanoparticles are incorporated into polymeric or ceramic scaffolds to impart magneto‐mechanical cues, enhance mechanical strength, and enable remote control of cell behavior. Magnetically responsive scaffolds containing Fe_3_O_4_, MnFe_2_O_4_, or hexaferrite fillers can transduce externally applied magnetic fields into subtle mechanical or thermal stimuli, promoting osteogenic differentiation, angiogenesis, and accelerated tissue regeneration in bone and soft‐tissue models. The presence of ferrites also improves scaffold electrical and dielectric properties, which can be advantageous for electroactive tissues such as nerve or muscle, while their intrinsic antibacterial activity helps mitigate infection at the implant–tissue interface. The integration of ferrite‐based nanomaterials, particularly SPIONs, has precipitated a paradigm shift in regenerative medicine from passive scaffolding to active, stimuli‐responsive tissue engineering. Beyond their established role in noninvasive magnetic resonance imaging (MRI) for tracking biodegradation, ferrites are increasingly utilized to orchestrate cellular behavior and fabricate complex three‐dimensional (3D) architectures through magnetic force‐based tissue engineering (Mag‐TE) [466, 467, 468].

**FIGURE 24:**
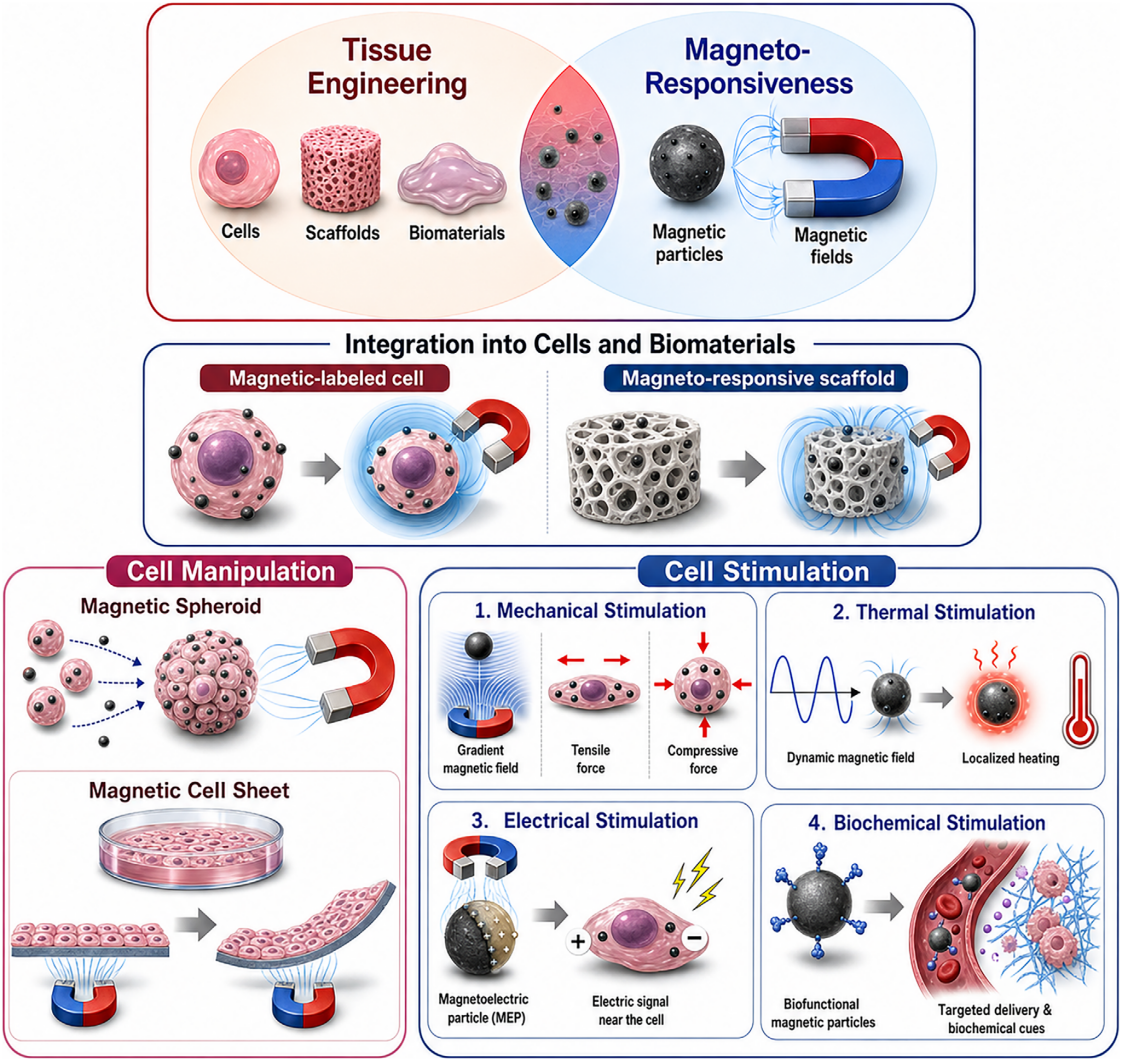
Conceptual overview of magnetic tissue engineering (MagTE), showing the integration of magnetic particles with cells, scaffolds, and biomaterials to enable magnetic cell manipulation and mechanical, thermal, electrical, and biochemical stimulation for tissue regeneration.

The fundamental physicochemical principles of magnetic particles and field dynamics bridge the gap between basic actuation mechanisms and advanced cell‐ and tissue‐level applications. Crucially, the analysis extends beyond static assembly to emphasize the integration of magnetic tissue engineering (MagTE) into dynamic biofabrication workflows, highlighting its pivotal role in promoting functional tissue maturation and facilitating clinical translation. Furthermore, the convergence of MagTE with emerging domains, specifically magnetic field‐based robotics, presents a transformative frontier that remains largely underexploited within current biofabrication paradigms. The future directions, along with strategic frameworks for the clinical implementation of MagTE, is illustrated in Figure 24 [51].

In hard tissue engineering, ferrites act as dual‐functional agents that enhance scaffold mechanical properties and provide osteoinductive cues. Incorporating SPIONs into hydroxyapatite (HA) or chitosan composites significantly improves compressive strength while promoting osteogenesis. A key mechanism driving this is the formation of a dynamic protein corona on the nanoparticle surface, which activates the MAPK/ERK signaling pathway to accelerate bone healing. Furthermore, magneto‐mechanical stimulation, exerting physical forces on SPIONs‐loaded scaffolds composed of piezoelectric polymers like polyvinylidene fluoride (PVDF), mimics the physiological environment of native bone, thereby enhancing stem cell differentiation. Magnetic gradients have also been used to guide BMP‐2‐loaded SPIONs within hydrogels, creating osteochondral constructs with distinct mineral transitions [51, 469, 470]. For instance, BaFe_12_O_19_ nanoparticles incorporated into polymer scaffolds have been shown to enhance osteogenic differentiation under magnetic stimulation [120, 471]. Fe_3_O_4_‐based composite scaffolds have demonstrated improved cell adhesion and proliferation [472], while MnFe_2_O_4_ nanoparticles have been reported to promote angiogenesis and tissue regeneration [473]. These studies highlight the role of magnetic stimulation in accelerating tissue repair processes.

Ferrites address critical challenges in vascular endothelialization and cardiac coupling. Magnetic forces can radially direct SPION‐labeled endothelial cells to luminal scaffold surfaces, achieving rapid and uniform coverage under flow conditions [474]. Mag‐TE has also facilitated the construction of scaffold‐free, multilayered cell sheets, such as ring‐shaped cardiac tissues that exhibit spontaneous synchronous beating. Crucially, the internalization of ferrites by cardiomyocytes is linked to the upregulation of the gap junction protein connexin 43 (Cx43), enhancing electrical propagation and integration with host myocardium [475, 476, 477]. In the nervous system, superparamagnetism is exploited to bridge nerve gaps and guide axons. Functionalized SPIONs act as mechanical actuators; for instance, magnetic translocation of particles into neurite tips induces filopodial elongation and directs axonal growth. In spinal cord injury, magnetically aligned hydrogels provide topographic cues for nerve sprouting, while SPIONs labeled enhance the targeting of Schwann cells to astrocyte‐rich domains [478]. Finally, in soft tissue engineering, Mag‐TE enables the construction of heterotypic liver tissues by magnetically stratifying cells. In wound healing, magnetic stimulation of bFGF‐functionalized SPIONs promotes sustained drug release and macrophage polarization toward the pro‐healing M2 phenotype [479, 480].

#### 8.6.2 Cell Tracking, Labeling, and Magnetically Guided Surgery

The clinical translation of cell‐based therapies is currently impeded by the inability to noninvasively monitor graft fate and ensure precise localization at the injury site [481]. Ferrite‐based nanomaterials, particularly SPIONS, address this translational bottleneck by functioning as dual‐purpose contrast agents and magnetic actuators. In the context of diagnostic tracking, the internalization of SPIONS allows for high‐resolution magnetic resonance imaging (MRI) of cell biodistribution, migration, and clearance over extended periods [482]. Unlike conventional fluorophores, the high magnetic susceptibility of ferrite cores generates strong T2/T2* hypointense contrast, enabling the visualization of small cell clusters within deep tissue structures such as the myocardium and spinal cord [483]. However, a critical challenge remains in distinguishing viable therapeutic cells from resident macrophages that have phagocytosed cellular debris. To overcome this, recent strategies employ multimodal labeling coupling ferrites with reporter genes (e.g., luciferase or fluorescence) to correlate magnetic signals with metabolic viability, ensuring that MRI contrast reflects functional engraftment rather than merely iron accumulation [484, 485].

Beyond passive monitoring, the intrinsic magnetic moment of ferrite nanoparticles facilitates magnetically guided surgery, a paradigm where external magnetic field gradients are utilized to actively direct and retain therapeutic agents against physiological forces. This is particularly advantageous in cardiovascular and neurological interventions, where hemodynamic washout and the blood–brain barrier (BBB) severely limit the retention of systemically or locally administered cells [486, 487]. By employing high‐gradient magnetic topologies, such as Halbach arrays or implantable magnetic stents, SPIONS‐loaded mesenchymal stem cells (MSCs) and endothelial progenitor cells (EPCs) can be tethered to target lesions. This magnetic targeting has been demonstrated to significantly enhance cell retention in infarcted myocardia and injured spinal cords, shifting the therapeutic window from an inflammatory response to reparative regeneration. Furthermore, this actuation capability extends to complex microsurgical applications, such as the magnetic retrieval of encapsulated pancreatic islets in diabetes therapy or the precise guidance of axonal growth cones in nerve repair [488, 489, 490, 491]. Consequently, ferrite‐based systems offer a seamless integration of image‐guided delivery and mechanical actuation, providing a robust platform for overcoming the physical barriers to tissue regeneration.

#### 8.6.3 Bioseparation, Purification, and Lab‐Scale Bioprocessing

Ferrite‐based magnetic nanoparticles (MNPs) have transformed bioseparation and bioprocessing workflows by replacing purely centrifugation‐ or filtration‐driven operations with controllable, field‐responsive platforms. In contrast to conventional solid supports, spinel ferrites such as magnetite Fe_3_O_4_, manganese ferrite MnFe_2_O_4_, and cobalt ferrite CoFe_2_O_4_ combine high saturation magnetization with chemical robustness and readily tailorable surface chemistry, enabling selective capture and release of target biomolecules in complex matrices. In lab‐scale settings, these nanostructures function as mobile solid phases that can be rapidly collected or re‐dispersed using external magnetic fields, eliminating high shear and centrifugal stress that typically compromise shear‐sensitive cells, vesicles, and protein complexes. This magnetically switchable behavior is readily integrated into microfluidic, lab‐on‐a‐chip, and small‐volume bioreactor systems, supporting parallelization and automation of bioprocessing steps.

a) Mechanistic advantages in nucleic acid extraction

The use of ferrite nanoparticles for nucleic acid isolation has enabled rapid, column‐free DNA and RNA purification with minimal instrumentation. Superparamagnetic MnFe_2_O_4_ and surface‐modified Fe_3_O_4_ particles can reversibly bind genomic and plasmid DNA under high‐salt conditions (for example, molar‐range NaCl or chaotrope‐containing buffers) and release it at low ionic strength, effectively acting as an on–off magnetic switch for nucleic acids [492]. In this configuration, the negatively charged phosphate backbone of DNA interacts with positively charged or amine/guanidinium‐functionalized ferrite surfaces through electrostatics and salt‐bridge formation, yielding high‐purity DNA comparable to commercial spin‐column kits but with substantially reduced processing time and without centrifugation [493, 494]. Importantly, the same chemistry can be downscaled to microliter volumes in integrated microfluidic cartridges or scaled up to milliliter–liter batch formats, demonstrating the intrinsic scalability of ferrite‐based nucleic acid capture for diagnostics, synthetic biology, and upstream bioprocessing [495, 496].

b) Precision protein purification

In protein purification, surface‐engineered ferrites are increasingly viewed as agile alternatives or complements to packed‐bed chromatography, particularly for affinity‐based capture of recombinant proteins. Silica‐coated cobalt ferrite SiO_2_‐CoFe_2_O_4_ nanoparticles functionalized with Ni–NTA (nitrilotriacetic acid) ligands exemplify this trend: the Ni^2 +^  centers on the silica shell form coordination complexes with histidine residues on His‐tagged proteins, while the magnetic core enables instantaneous separation of the protein–nanoparticle complex from crude lysates using a simple magnet [497, 498]. The silica interlayer prevents oxidation and dissolution of the ferrite core and provides a chemically robust, low‐fouling platform for ligand anchoring, thereby reducing nonspecific adsorption and improving purity relative to nonmagnetic particulates [499]. Because mass transfer is governed by nanoscale diffusion rather than convective flow through packed beds, these ferrite‐based systems often exhibit faster binding kinetics and lower back‐pressure, which is advantageous for high‐throughput screening, small‐batch production of therapeutic proteins, and parallel optimization of purification conditions [500].

c) Magnetic Activated Cell Sorting and Cellular Bioseparation

For cellular bioseparation, the superparamagnetic behavior of ferrite nanoparticles is critical: particles must exhibit strong magnetization in the field yet negligible remanence once the field is removed, to avoid irreversible aggregation and preserve cell viability [501]. Ferrite‐based magnetic activated cell sorting (MACS) leverages antibody‐ or ligand‐conjugated nanoparticles to label specific surface markers on rare cell populations such as hematopoietic stem cells, mesenchymal stromal cells, or circulating tumor cells directly in complex suspensions, including whole blood [502, 503]. Upon application of a magnetic field gradient, labeled cells are selectively retained while unlabeled cells are washed away, delivering separation purities comparable to flow cytometry‐based sorting but with higher throughput, gentler handling, and lower equipment complexity [504]. Optimizing core size and hydrodynamic diameter is essential: cores must be large enough to respond efficiently to magnetic gradients yet small enough (typically below ∼100 nm) to maintain colloidal stability, minimize opsonization, and limit perturbation of cell physiology [505, 506, 507]. Combined with microfluidic channel design and multiplexed marker panels, ferrite‐enabled MACS is evolving into a key enabling technology for lab‐scale bioprocessing, including immunotherapy manufacturing, organoid preparation, and preparative isolation of extracellular vesicles [112].

These orthogonal applications underscore how ferrite nanoparticles transcend their role as mere separation aids to become active components of integrated bioprocessing pipelines, where magnetic actuation synchronizes capture, washing, elution, and downstream analysis in continuous or batch formats. Continued advances in core–shell architectures, stimuli‐responsive ligands, and automated magnetic manipulators promise to further embed ferrites within industrial‐scale workflows for cell therapies, biologics production, and precision diagnostics.

## 9 Current Challenges and Opportunities

Despite the remarkable progress in tailoring the magnetic and physicochemical properties of ferrite nanoparticles for high‐performance biomedical applications, a substantial divide remains between laboratory innovation and clinical reality. While theoretical and pre‐clinical studies have demonstrated the immense potential of these materials for imaging, hyperthermia, and drug delivery, realizing their full therapeutic value requires navigating a complex landscape of translational hurdles. Despite significant progress in material design, the clinical translation of ferrite‐based nanomaterials is constrained by regulatory and safety limitations, which necessitate quantitative evaluation of performance metrics. In magnetic hyperthermia, the product of magnetic field strength (*H*) and frequency (*f*) is typically restricted to approximately 5 × 10^9^ A m^−1^ s^−1^ to minimize adverse physiological effects. Consequently, reported high SAR values must be interpreted within these clinically acceptable limits. In addition, nanoparticle dosage, biodistribution, and clearance rates are critical factors influencing safety and regulatory approval. While typical experimental studies employ elevated nanoparticle concentrations to achieve therapeutic effects, clinically relevant doses must ensure minimal toxicity and efficient clearance through renal or hepatic pathways. These considerations highlight that achieving optimal performance requires balancing magnetic efficiency, biocompatibility, and pharmacokinetics within regulatory constraints.

The following section critically examines the primary bottlenecks impeding the commercialization and clinical adoption of ferrite‐based nanomedicines, ranging from regulatory compliance and biological unpredictability to the fundamental challenges of large‐scale manufacturing.

### 9.1 Issues in Translation to Clinical Applications and Regulatory Perspectives

The transition of ferrite‐based nanomaterials from laboratory prototypes to clinically approved agents faces several significant hurdles that bridge material science and regulatory compliance.

1. The translational gap: There is a marked disconnect between academic research and clinical implementation. While research has developed high‐performance “next‐generation” heat mediators (e.g., nanocubes, octopods) with superior Specific Absorption Rates (SAR), current clinical trials (such as those for glioblastoma) still rely on conventional spherical iron oxide nanoparticles synthesized via hydrolytic routes. Bridging this divide requires moving beyond discovery to developing protocols that meet Good Manufacturing Practice (GMP) standards.
2. In vitro versus in vivo efficacy discrepancy: A major clinical challenge is the discrepancy between heating efficiency measured in the lab and that observed in biological systems. Standard calorimetric characterization often overestimates therapeutic potential because it neglects the intracellular environment; recent studies show heating efficiency can drop by up to 90% post‐internalization due to lysosomal clustering and high intracellular viscosity.
3. Regulatory and safety hurdles: Clinical translation is impeded by the need for rigorous, standardized evaluation of long‐term biocompatibility, biodistribution, and clearance pathways. Although iron oxides like Fe_3_O_4_ are generally considered safe, other ferrite compositions (e.g., those doped with Ni or Co) require careful toxicity assessment due to potential metal ion leaching. Regulatory bodies require predictable biological fates, yet issues such as off‐target accumulation in the liver and spleen remain unresolved.
4. Standardization needs: There is a pressing need to update standardization protocols. Current measures often fail to account for viscosity‐dependent susceptibility, meaning reported SAR values may not predict actual intratumoral behavior. Future developments must establish standardized biological evaluation protocols for toxicity and long‐term effects to ensure clinical viability.

### 9.2 Stability, Scalability, and Reproducibility of Ferrite‐Based Biomedical Agents

For ferrite nanoparticles to become viable pharmaceutical products, they must overcome strict manufacturing and stability constraints.

1. Scalability versus performance: A significant translational gap exists regarding synthesis methods. Thermal decomposition routes yield superior crystallographic integrity and precise morphological control (essential for high‐performance imaging and hyperthermia) but are difficult to scale compared to aqueous synthesis methods. The challenge lies in developing scalable, green synthesis protocols that can produce these complex nanostructures without compromising their magnetic functionality.
2. Reproducibility: The biomedical performance of ferrites is highly sensitive to physical parameters; deviations in particle size, shape, or crystal structure can critically undermine magnetic functionality and therapeutic effectiveness. Achieving reproducible synthesis that guarantees consistent particle morphology and size distribution across batches is a prerequisite for clinical use.
3. Colloidal stability in physiological media: In complex biological fluids, ferrite nanoparticles face high ionic strength and competing biomolecules, which promote aggregation and the formation of a protein corona. Instability leads to agglomeration, which alters biodistribution, toxicity profiles, and magnetic response.
4. Surface engineering opportunities: Addressing stability requires advanced surface engineering. Functional coatings (e.g., silica, PEG, and dextran) are critical not just for biocompatibility but for maintaining colloidal stability and preventing rapid clearance by the reticuloendothelial system. The development of robust surface chemistries that prevent nonspecific protein adsorption (biofouling) while preserving catalytic or binding sites remains a primary challenge for next‐generation diagnostic devices.

In summary, while the path to clinical translation is fraught with challenges related to physiological stability, batch‐to‐batch reproducibility, and regulatory standardization, these obstacles also present opportunities for material innovation. Overcoming the “translational gap” will necessitate a paradigm shift toward scalable, green synthesis protocols and the development of viscosity‐independent heating mechanisms that retain efficacy within the intracellular environment. Ultimately, a concerted interdisciplinary effort focusing on the convergence of advanced surface engineering, rigorous toxicity profiling, and standardized manufacturing practices will be essential to transform ferrite‐based prototypes into safe, commercially viable, and clinically effective nanomedicines.

## 10 Future Trends and Emerging Directions

Future advancements in ferrite‐based nanomaterials should move beyond static material optimization toward adaptive, multifunctional, and clinically integrated systems. Recent developments provide clear directions for this transition. For instance, frequency‐tunable heterojunction systems reported by Zhang et al. [133] demonstrate that dynamic control of excitation frequency enables precise modulation of therapeutic response, offering opportunities for real‐time optimization of hyperthermia and neuromodulation. Similarly, regenerative nanomaterials such as layered double hydroxides (LDHs), as highlighted by Luo et al. [110], emphasize the importance of bioactive ion release and tissue integration, suggesting that future ferrite‐based systems should incorporate biofunctional components to enhance regenerative performance. In addition, advances in 3D‐printed multifunctional scaffolds, such as those demonstrated by Kanwar et al. [123], highlight the potential of integrating structural support, magnetic responsiveness, and therapeutic functionality within a single platform, enabling simultaneous tissue regeneration and localized therapy.

As the field of ferrite‐based nanomedicine matures, the focus is shifting from fundamental characterization to the strategic design of next‐generation materials that can overcome existing clinical barriers. While current research has established the baseline utility of spherical iron oxides, the demand for higher therapeutic efficacy and multifunctionality is driving a paradigm shift toward atomically precise architectures and intelligent systems. The following section outlines the critical trajectory for future innovations, highlighting how advances in material synthesis, functionalization, and system‐level integration will redefine the capabilities of ferrite nanoplatforms in the coming decade.

**FIGURE 25:**
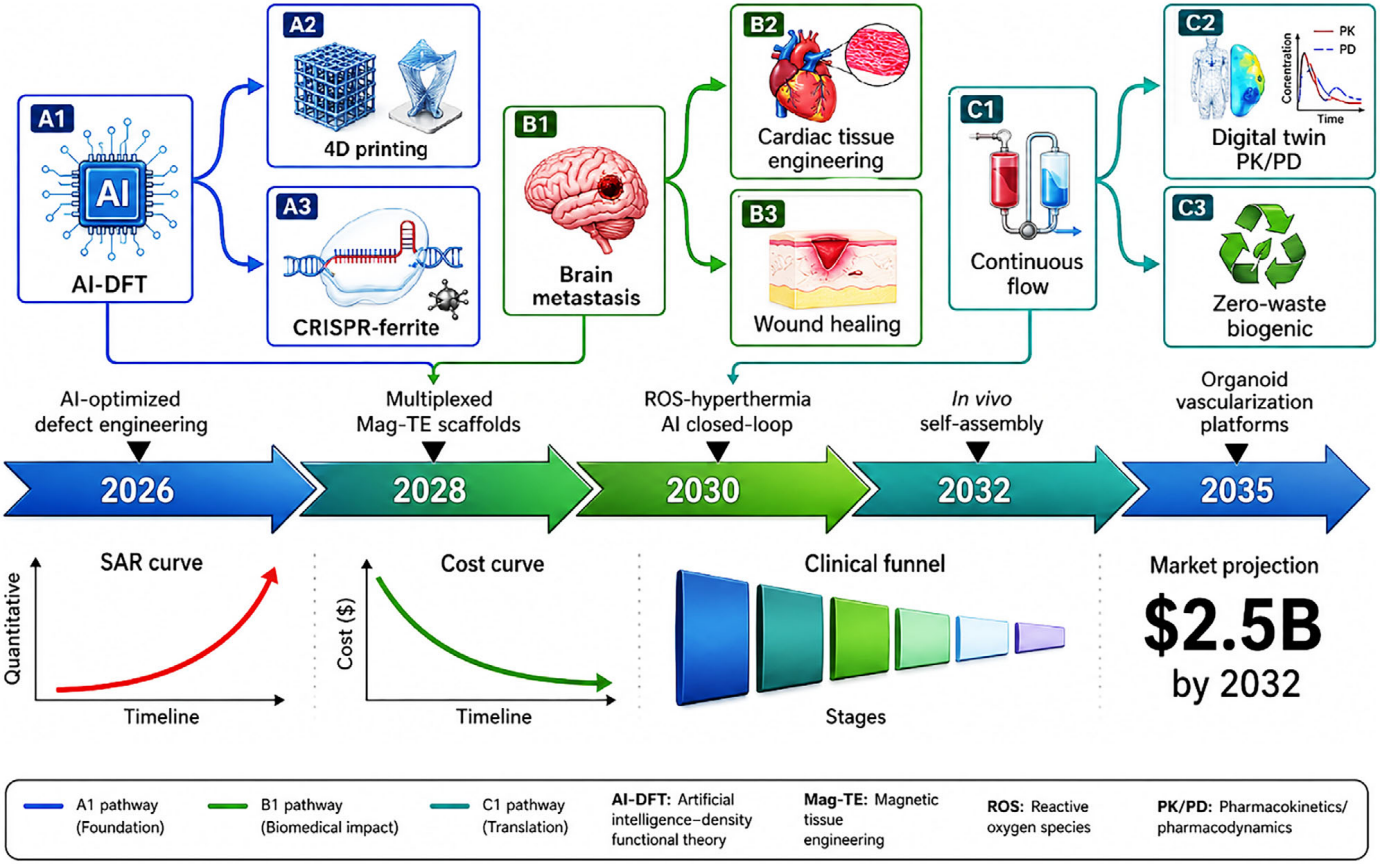
Conceptual forward‐looking roadmap for ferrite‐based biomedical technologies, highlighting the progression from AI‐assisted materials design to biomedical application and clinical translation. The figure summarizes projected milestones from 2026 to 2035, including AI‐optimized defect engineering, multiplexed Mag‐TE scaffolds, ROS‐hyperthermia with AI closed‐loop control, in vivo self‐assembly, and organoid vascularization platforms, together with expected trends in SAR, cost reduction, clinical progression, and market growth.

Figure 25 illustrates the strategic ten‐year roadmap for advancing spinel ferrite nanomedicine from experimental research to commercial scalability. The schematic is divided into three converging tracks: (A) Technology convergence, featuring AI‐driven material discovery and 4D printing; (B) clinical expansion, highlighting diverse applications in neuro‐oncology and tissue engineering; and (C) manufacturing & biosafety, emphasizing the shift toward continuous flow synthesis and zero‐waste biogenic production. The central timeline projects the evolution of these systems into personalized, closed‐loop theranostic platforms by 2035. Future research should focus on bridging the gap between laboratory‐scale performance and clinical requirements by integrating material design with biological validation. Particular emphasis should be placed on achieving high efficiency under clinically safe conditions, long‐term in vivo stability, and scalable synthesis routes. The development of multifunctional ferrite nanoplatforms with tunable properties offers significant opportunities for next‐generation theranostic applications.

Based on these advances, several key research directions can be proposed:

- Multi‐field coupling systems: Development of ferrite‐based platforms responsive to combined magnetic, thermal, electrical, and acoustic stimuli to achieve synergistic therapeutic effects.
- Frequency‐tunable and adaptive systems: Engineering materials capable of dynamic response under variable excitation conditions to optimize performance in complex physiological environments.
- In situ monitoring and feedback systems: Integration of sensing capabilities (e.g., magneto‐responsive biosensors) to enable real‐time tracking of temperature, drug release, and biological response.
- Hybrid regenerative platforms: Combining ferrites with bioactive materials (e.g., LDHs, polymers, or metal ions) to achieve simultaneous therapeutic and regenerative functions.
- Scalable and green synthesis pipelines: Development of reproducible, low‐cost, and environmentally sustainable fabrication strategies compatible with industrial and clinical translation.
- Device–material co‐design: Integration of nanoparticle systems with clinically compatible field generators and biomedical devices to ensure performance within regulatory constraints.

These directions highlight a paradigm shift from isolated material development toward integrated, adaptive, and clinically translatable nanoplatforms, which will define the next generation of ferrite‐based biomedical technologies. The trajectory of ferrite‐based nanomedicine is poised at a critical inflection point, shifting from the exploration of passive capabilities to the rational design of active, smart architectures. Future research must bridge the divide between crystallographic perfection and biological reality, driving innovations that enable clinical translation.

### 10.1 Innovations in Synthesis, Functionalization, and Hybrid Materials

The next decade of material development will likely move beyond simple spherical morphologies toward precise atomic and architectural engineering.

1. From colloidal synthesis to atomic precision: While hydrolytic routes offer scalability, the future lies in non‐hydrolytic thermal decomposition and “green” biogenic methods that yield highly crystalline, anisotropic nanostructures (e.g., nanocubes, octopods, and nanoflowers). These shapes possess superior magnetic anisotropy, which is critical for maximizing Specific Absorption Rates (SAR) in hyperthermia applications. Future synthesis will increasingly focus on defect engineering and atomically precise doping (e.g., with rare‐earth ions or Zn/Co substitution) to modulate electronic band structures and enhance catalytic ROS generation for antimicrobial and anticancer therapies.
2. Viscosity‐independent nanoheaters: A critical emerging direction is the design of materials that retain their heating efficiency within the highly viscous intracellular environment. Research is pivoting toward exchange‐coupled core–shell systems and pre‐assembled magnetic chains that rely on Néel relaxation rather than Brownian motion, ensuring predictable therapeutic performance inside tumors.
3. Next‐generation hybrids and stealth surfaces: The development of hybrid composites such as Ferrite@MOFs (metal–organic frameworks) or Ferrite@Plasmonic (Au/Ag) will expand to create all‐in‐one platforms combining magnetic manipulation with photothermal and catalytic functionalities. Simultaneously, surface functionalization strategies will evolve from simple polymer coating to protein corona engineering. By designing surfaces that selectively recruit specific plasma proteins, researchers aim to create truly stealth nanoparticles that evade immune clearance while maintaining targeting specificity.

### 10.2 Integration With Multifunctional and Smart Biomedical Systems

The ultimate goal is the seamless integration of ferrite nanomaterials into intelligent, stimuli‐responsive systems capable of real‐time diagnosis and adaptive therapy.

1. Closed‐loop magnetotheranostics: Future platforms will move beyond static “diagnose‐and‐treat” models to dynamic, closed‐loop systems. These “smart” agents will utilize environmental cues (such as tumor pH, redox gradients, or enzymatic activity) to trigger drug release or activate catalytic properties only at the disease site. This includes the development of ratiometric imaging probes (like dual‐mode T1/T2 MRI agents) that provide self‐calibrating feedback on therapeutic progress.
2. Magnetic tissue engineering (Mag‐TE) and robotics: A transformative frontier involves utilizing ferrites as active mechano‐transducers in regenerative medicine. By incorporating magnetic nanoparticles into scaffolds, researchers can apply external fields to mechanically stimulate stem cell differentiation, guide axonal growth, or construct complex 3D tissue architectures via magnetic levitation. This aligns with the emerging field of magnetic microrobotics, where ferrite‐based agents are magnetically steered through the vasculature to perform microsurgery or targeted biopsy.
3. AI‐driven material discovery: Finally, the integration of Artificial Intelligence (AI) and machine learning will revolutionize the design process. Computational models will be employed to predict the biological identity of nanoparticles (such as protein corona composition, toxicity profiles) before synthesis, accelerating the screening of safe and effective formulations.

In conclusion, the next generation of ferrite‐based biomedical agents will be defined not merely by their intrinsic magnetic properties, but by their ability to function as dynamic, decision‐making components within complex biological environments. By converging atomic‐level material design with smart responsiveness and artificial intelligence‐driven discovery, these emerging technologies promise to bridge the gap between laboratory innovation and clinical reality. Realizing this vision will require a concerted interdisciplinary effort to master the bio–nano interface, ensuring that these sophisticated nanoplatforms are as safe and reproducible as they are effective.

## 11 Conclusions

This review highlights the transformative trajectory of ferrite‐based nanomaterials, evolving from passive magnetic carriers into sophisticated, active regulators of biological processes. The intrinsic versatility of the spinel and hexagonal lattices allows for precise atomic‐level engineering, where the manipulation of cation distribution, morphology, and surface chemistry dictates functionality. As detailed throughout this work, these structural modifications have unlocked a broad spectrum of biomedical capabilities, ranging from high‐contrast MRI and magnetic particle imaging to viscosity‐independent hyperthermia and targeted antimicrobial action. A defining feature of modern ferrite research is the paradigm shift toward integrated magnetotheranostics. The ability to combine diagnostic precision with therapeutic intervention such as coupling MRI guidance with stimuli‐responsive drug release or localized thermal ablation within a single nanoconstruct represents a significant leap toward personalized medicine. Furthermore, the emergence of ferrite‐based nanozymes and electrochemical biosensors has pushed the limits of detection for cancer biomarkers and pathogens, offering robust alternatives to biological enzymes in point‐of‐care diagnostics.

However, despite these theoretical and preclinical successes, a formidable translational gap remains between laboratory innovation and clinical reality. The discrepancy between laboratory‐measured specific absorption rates (SAR) and in vivo heating efficiency, often diminished by intracellular confinement and viscosity, underscores the need for characterization protocols that accurately mimic physiological environments. Moreover, while high‐performance anisotropic nanostructures like nanocubes and octopods demonstrate superior magnetic properties, their translation is currently hindered by the lack of scalable, Good Manufacturing Practice (GMP)‐compliant synthesis protocols compared to traditional spherical oxides. Future research must focus on developing green, reproducible synthesis routes that maintain the crystallographic integrity of complex nanostructures at an industrial scale. A deeper understanding of the protein corona and its influence on biodistribution, immune recognition, and long‐term clearance is essential to engineer stealth surfaces that ensure safety and targeting efficacy.

In summary, ferrite‐based materials stand at the forefront of nanobiotechnology, offering an unparalleled combination of magnetic tunability and biological adaptability. By converging advances in materials science with rigorous biological validation, the next generation of ferrite nanoplatforms is poised to overcome current limitations, enabling precise, noninvasive, and multi‐modal solutions for the most challenging clinical problems.

## Conflicts of Interest

The authors declare no conflicts of interest.

## Data Availability

The data that support the findings of this study are available from the corresponding author upon reasonable request.
